# A multiple coefficient of determination-based method for parsing SNPs that correlate with mRNA expression

**DOI:** 10.1038/s41598-019-56494-9

**Published:** 2019-12-27

**Authors:** Fan Song, Yu Tao, Yue Sun, David Saffen

**Affiliations:** 1 Department of Cellular and Genetic Medicine, School of Basic Medical Sciences, Fudan University, Shanghai, 200032 China; 2 Institutes of Brain Science, Fudan University, Shanghai, 200032 China; 3 State Key Laboratory for Medical Neurobiology, Fudan University, Shanghai, 200032 China; 4 School of Life Sciences, Fudan University, Shanghai, 200438 China

**Keywords:** Gene expression profiling, Gene expression

## Abstract

In this study, we present a novel, multiple coefficient of determination (R^2^_M_)-based method for parsing SNPs located within the chromosomal neighborhood of a gene into semi-independent families, each of which corresponds to one or more functional variants that regulate transcription of the gene. Specifically, our method utilizes a matrix equation framework to calculate R^2^_M_ values for SNPs within a chromosome region of interest (ROI) based upon the choices of 1-4 “index” SNPs (iSNPs) that serve as proxies for underlying regulatory variants. Exhaustive testing of sets of 1–4 candidate iSNPs identifies iSNP models that best account for estimated R^2^ values derived from single-variable linear regression analysis of correlations between mRNA expression and genotypes of individual SNPs. Subsequent genotype-based estimation of pairwise r^2^ linkage disequilibrium (LD) coefficients between each iSNP and the other ROI SNPs allows the SNPs to be parsed into semi-independent families. Analysis of mRNA expression and genotypes data downloaded from Gene Expression Omnibus (GEO) and database for Genotypes and Phenotypes (dbGAP) demonstrates the usefulness of this method for parsing SNPs based on experimental data. We believe that this method will be widely applicable for the analysis of the genetic basis of mRNA expression and visualizing the contributions of multiple genetic variants to the regulation of individual genes.

## Introduction

Identifying genetic variants that correlate with gene expression and elucidating their underlying molecular mechanisms are major goals in the field of human genetics^1–3^. Information generated from these studies is often useful for the annotation of SNPs that associate with human disorders in genomewide association studies (GWAS) and for identifying causal variants that contribute to human disorders^4^. Understanding how expression of specific genes, especially the high- or low-expression extremes, contribute to the etiology of human disorders is an important step toward developing new methods for diagnosing disorder subtypes and for identifying possible molecular targets for novel drugs to treat, slow or prevent their development^5,6^.

Quantitative genome-wide association studies (GWAS) have identified a large number of genetic variants that correlate with mRNA expression of nearby genes^7^ and these expression “quantitative trait loci” (eQTL) are often useful for annotating individual genetic markers, usually single nucleotide polymorphisms (SNPs), that correlate with disease liability or protection in genome-wide association studies (GWAS). Although the majority of “expression” SNPs (eSNPs) do not directly function as regulatory genetic variants, their correlation with mRNA expression may result from being in LD with one or more regulatory variants. The initial focus on the association of single eSNPs with human disorders, however, may result in the failure to detect associations that depend upon multiple genetic variants that influence the expression of individual genes, for example in cases where disorder liability or protection occurs only at the extremes of mRNA expression that are not captured by a single genetic marker^8,9^. Identifying sets of eSNPs that accurately capture the full range of gene expression for use in genetic association studies therefore remains an important goal. Current methods that assess the contributions of multiple genetic variants to mRNA expression of a single gene include haplotype-, regression- and Bayesian statistics-based approaches^10–13^. While powerful, these methods may still fail to capture the complete landscape of genetic regulation for individual genes.

In this study, we describe a novel method for analyzing the combined contribution of regulatory variants to mRNA expression based on the analysis of coefficients of determination (R^2^) derived from single-variable linear regression analysis of individual SNPs located within defined chromosome regions of interest (ROI). We believe that this method will be useful for both assessing the minimum number of independent regulatory variants that influence the expression of a given gene and identifying families of SNPs that are in LD with these variants. A potential application of our approach is the identification of sets of SNPs that will serve as more effective markers in genetic association studies.

## Results

### Mathematical foundation

We previously described a multiple linear regression-based method for parsing SNPs that correlate with mRNA expression into semi-independent families^8,14^. The underlying assumptions of this method were: (i) non-regulatory SNPs correlate with mRNA expression to the extent to which they are in LD with regulatory variants, and (ii) semi-independent “families” of SNPs that correlate with mRNA expression reflect the underlying contributions of one or more regulatory variants. Although this ‘ad hoc’ method produced useful results for many of the genes we analyzed, mathematical modeling revealed significant inconsistencies, in particular with respect to SNPs with highly correlated genotypes and/or SNPs with minor contributions to the variance of mRNA expression.

During a search for a mathematically sound method for parsing SNPs that correlate with mRNA expression, we learned of a well-known result from multivariate statistical analysis that allows population multiple coefficients of determination (R^2^_M_) to be calculated based on the matrix equation R^2^_M_ = **C**^T^**R**^−1^ **C**, where **C** is a column vector (r_YX1_, r_YX2_, …..r_YXp_), with elements equal to the Pearson correlation coefficients between a dependent variable Y and one or more independent variables, (X_1_, X_2_ …. X_k_), **C**^T^ is row vector equal to the transpose of **C**, and **R**^−1^ is the inverse of the correlation matrix **R** for the independent variables (modified nomenclature based on equations in:^15^). Elements of **R** comprise Pearson correlation coefficients for all pairwise comparisons of the independent variables: r_X1X1_, r_X1X2_ … r_XiXj_.…..r_XpXp_. In the derivations that follow, we found it useful to use the well-known identity equation: **R**^−1^ = adjugate of **R**/determinant of **R = **adj**R** /det**R**.

The first step toward using the above equations to develop a method for parsing SNPs that correlate with mRNA expression was to define r_YG1_, r_YG2_, ….. r_YGp_ as the Pearson correlation coefficients between mRNA expression levels (Y) and genotypes of a small set of regulatory variants (G_1_, G_2_ …. G_p_) and r_G1G1_, r_G1G2_ … r_GiGj_.…..r_GpGp_ as the Pearson correlation coefficients between genotypes for all pairwise combinations of these regulatory variants.

To simplify our notation, we designated individual non-regulatory SNPs as SNP_A_, and bi-allelic regulatory variants as SNP_B_, SNP_C_, SNP_D_, etc. Based on this notation, we also designated r_YG1_ = r_YA_, r_YG2_ = r_YB_, etc., and r_G1G1_ = r_AA_, r_G1G2_ = r_AB_, etc. It should be noted that (r_YA_)^2^ is equal to the single variable linear regression-based coefficient of determination for SNP_A_ = R^2^_A_ and (r_AB_)^2^ is an r^2^ LD estimator for the pair of SNPs, SNP_A_ and SNP_B_. We also used the notation: R^2^_M_ = R^2^_AB_ for systems comprising SNP_A_ and SNP_B_, R^2^_M_ = R^2^_ABC_ for systems comprising SNP_A_, SNP_B_ SNP_C_, etc., where the subscript “M” stands for “multiple.”

**Figure 1:**
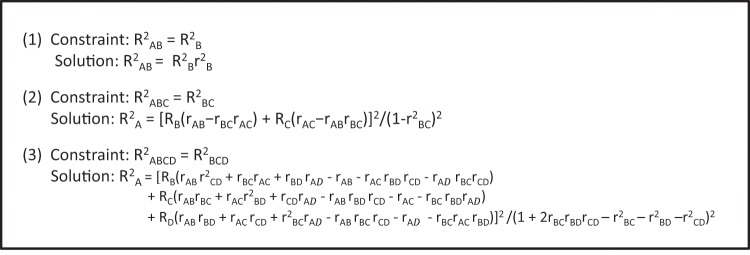
Solutions to polynomial equations derived from “constraint” matrix equations.

The next step in using the matrix equation to calculate coefficients of determination (R^2^_A_) for non-regulatory SNP_A_ in LD with multiple regulatory variants SNP_B_, SNP_C_, etc., was to derive explicit equations for R^2^_A_ that are consistent with the constraints: R^2^_AB_ = R^2^_B_, R^2^_ABC_ = R^2^_BC_, R^2^_ABCD_ = R^2^_BCD_,or R^2^_ABCDE_ = R^2^_BCDE_. The restrictions on the values of R^2^_A_ in these equations reflect the assumption that, within each model, SNP_A_ simply correlates with, but does not directly contribute to mRNA expression, beyond the contributions made by SNP_B_, SNP_C_, etc., which directly contribute to mRNA expression or are in high LD with actual regulatory variants. Manually solving these “constraint” matrix equations for R^2^_A_ in systems comprising one non-regulatory SNP (SNP_A_) and one-, two- or three-regulatory variants (SNP_B_, SNP_C_, SNP_D_) yielded the equations for R^2^_A_ listed in rows 1–3 of Table 1 and revealed a pattern that presumably extends to systems containing higher-numbers of regulatory variants (Table 1, rows 4-N). When expressed in terms of coefficients of determination and Pearson correlation coefficients, the equations defining R^2^_A_ take on the forms for 2-, -3 and 4-SNP systems listed in Fig. 1.

**Table 1:** Solutions to higher-order “constraint” matrix equations.

	Bi-allelic regulatory variants or index SNPs in constraint matrix equation	Values of R^2^_A_ = β^2^/4α^2^ for non-regulatory/non-index SNPs
1	SNP_B_	(R_B_b_12_)^2^/b_11_^2^
2	SNP_B_, SNP_C_	(R_B_b_12_ + R_C_b_13_)^2^/b_11_^2^
3	SNP_B_, SNP_C_, SNP_D_	(R_B_b_12_ + R_C_b_13_ + R_D_b_14_)^2^/b_11_^2^
4	SNP_B_, SNP_C_, SNP_D_, SNP_E_	(R_B_b_12_ + R_C_b_13_ + R_D_b_14_ + R_E_b_15_)^2^/b_11_^2^
5	SNP_B_, SNP_C_, SNP_D_, SNP_E_, SNP_F_	(R_B_b_12_ + R_C_b_13_ + R_D_b_14_ + R_E_b_15_ + R_F_b_16_)^2^/b_11_^2^
N	SNP_B_, SNP_C_, SNP_D_, SNP_E_, SNP_F_,… SNP_N_	(R_B_b_12_ + R_C_b_13_ + R_D_b_14_ + R_E_b_15_ + R_F_b_16_ + … + R_N_b_1N_)^2^/b_11_^2^

b_11_, b_12_, etc. are elements of the adjugate matrix (adj**R**) of the correlation matrix **R**, defined for each set of bi-allelic regulatory variants or index SNPs (SNP_B_, SNP_C_, etc.) and β and α refer to terms in the quadratic equation used to solve the polynomial equations derived from the “constraint” matrix equations for R_A_: R_A_ = ─β ± (β^2^ − 4αγ)^1/2^/2α = −β/2α, since (β^2^ − 4αγ)^1/2^ = 0 under the defined constraints. (See Supplementary Files 1 and 3 for details concerning mathematical notation and equation derivations).

It should be noted that the equation for R^2^_A_ in a two-SNP system describes the expected result that the contribution of a non-regulatory variant (SNP_A_) to mRNA expression (R^2^_A_) is equal to the contribution of a regulatory variant SNP_B_ with which it is in LD (R^2^_B_) multiplied by the r^2^ LD coefficient for the two SNPs (r^2^_AB_). Equations for systems containing increasing numbers of regulatory variants, however, become increasing complex, with the number of additive terms in the polynomial expansions of the matrix equations defining R^2^_A_ for 3- and 4-SNP systems, for example, equal to (4)^2^ = 16 and (18)^2^ = 324, respectively. Based on our inferred pattern for constructing these equations shown in Table 1, however, calculations of values for R^2^_A_ can be easily and rapidly carried out using a computer. (See Supplementary Files 1–3 for a summary of the mathematical notation used in this paper and overviews of the derivations of R^2^_A_ for 2-, 3-, and 4- SNP systems).

### Analysis of simulated mRNA expression/genotype datasets

To determine whether the equations described above can accurately predict values for coefficients of determination obtained from linear regression analysis of mRNA expression vs. SNP genotype in experimental datasets, we carried out a series of calculations using simulated mRNA expression/genotype datasets for one non-regulatory SNP (SNP_A_) and one or more biallelic regulatory variants. The goal of these analyses was to confirm that our method accurately predicts R^2^_A_ values for pre-assigned sets of regulatory- and non-regulatory SNPs, prior to using the method to analyze experimental data, where the identities of regulatory- and non-regulatory SNPs are unknown.

Using custom R-language based programs developed in our laboratory, sets of population genotypes for 2–5 SNPs (SNP_A_, SNP_B_, SNP_C_, SNP_D_, SNP_E_), each exhibiting a wide range of allele frequencies and pairwise r^2^ LD values were constructed from sets of haplotypes with randomly assigned population frequencies that sum to 1. Values for mRNA expression were assigned based on the genotypes of the regulatory variants (SNP_B_, SNP_C_, SNP_D_, SNP_E_) in each model using Fisher’s “genotypic value” framework. (See Supplementary Files 2 and 4 for details.) Genotypes were coded 0, 1, or 2, based on the number of minor alleles within the simulated dataset under investigation. Alleles of regulatory variants were assumed to have additive effects, an assumption often appropriate for eQTLs^16^. Each simulation produced a virtual “spread sheet” with one column of mRNA expression values, one column of genotypes for the non-regulatory variant SNP_A_ and 1–4 columns of genotypes for SNP_B_, SNP_C_, SNP_D_, and SNP_E_, depending upon the number of biallelic regulatory variants included in the model. The population size for each simulation was typically n = 1000 and multiple simulations, typically 1000–3000, were carried out to construct datasets for analysis.

In addition to providing a value for R^2^_A_ (derived from linear regression analysis of mRNA expression levels vs. genotypes of the non-regulatory variant), data in each virtual spreadsheet allowed the calculation of Pearson correlation coefficients for: (i) correlations between mRNA expression and genotypes for individual regulatory variants and (ii) pairwise correlations between regulatory variant genotypes, that are required for matrix-equation-based calculation of R^2^_A_. As shown in Supplementary File 5, Fig. S1, comparisons of “estimated” values for R^2^_A_ and values of R^2^_A_ predicted using our matrix-based equations for two-, three- and four-regulatory variant systems yielded nearly identical results.

In a second round of simulations, we constructed datasets for analysis with genotypes for each individual derived from haplotype frequencies calculated using the polynomial equations listed in Supplementary File 4, Section D, with the values of estimators of minor allele frequencies and pairwise second-order (D_AB_) and third-order (D_ABC_) D linkage disequilibrium coefficients^17^ for non-regulatory and regulatory SNPs used as input variables. (See Supplementary File 2 and 4 for details). This method for constructing datasets was used to more realistically mimic experimentally derived datasets obtained from human mRNA expression/genotype data. As shown in Supplementary File 5, Fig. S2 our matrix equation-based method again produced excellent agreement between estimated and predicted R^2^_A_ values.

The results described above confirm the accuracy of our derived expressions for R^2^_A_ based on: (i) solving complex polynomial equations derived from our “constraint” matrix equations (Fig. 1 and Supplementary File 3) and (ii) our inferred general solutions to these equations (Table 1). Together, these establish a novel approach for analyzing the combined contributions of multiple regulatory variants to mRNA expression.

### Analysis of mRNA/genotype data for *methylene tetrahydrofolate reductase* (*MTHFR*) expression in human brain and lymphoblastoid cell lines

In this section, we provide an example of how our method can be used to analyze experimental mRNA expression/genotype data. Unlike the simulated data sets described above, the regulatory variants that influence mRNA expression for most genes are unknown. For this reason, we used our method to identify individual SNPs or combinations 1, 2, 3 or 4 SNPs (SNP_B_, SNP_C_, SNP_D_, SNP_E_) selected from a set of genotyped or imputed SNPs within a chromosome region of interest (ROI) that best served as proxies for unknown regulatory variants. In the context of our method, candidate proxy SNPs are termed “index SNPs” (iSNPs). Criteria for identifying the best iSNPs that fit simulated and experimental data are described below.

We began by calculating a single-variable linear regression-based, sample coefficient of determination for mRNA expression vs. genotype for each SNP in the ROI (i.e., R^2^_A1_, R^2^_A2_, R^2^_A3_,….., R^2^_An_ values for n SNPs in a chromosome ROI). In our notation, these are the “estimated” sample R^2^_A_ values for these SNPs. The next step was to calculate sample Pearson correlation coefficients for: i) mRNA expression vs. genotype (r_YGi_) and ii) genotype vs. genotype (r_GiGj_) for each SNP in the ROI. In our current study, individual-level mRNA expression and genotype data were obtained from two human brain datasets: “BrainCloud” (BC)^18^ and “4BrainR”^19^ and a human lymphoblastoid cell line (LCL) dataset^20^. See Supplementary File 2 for details, including Gene Expression Omnibus (GEO) and database for Genotypes and Phenotypes (dbGAP) dataset identifiers.

Using our R language-based program, sets of 1, 2, 3 or 4 ROI SNPs were selected to generate 1-, 2-, 3- or 4-iSNP models for calculating R^2^_A_ values based on the equations listed in Fig. 1 and Table 1. An independent calculation of R^2^_A_ was carried out for each of the n SNPs in the chromosome ROI for each possible 1-, 2-, 3- or 4-iSNP model. In our notation, the result of each independent calculation is the “calculated” R^2^_A_ value. The input variables for the equations used to calculate R^2^_A_ values were R_B_ = r_YB_, R_C_ = r_YC_, R_D_ = r_YD_, and R_E_ = r_YE_ and r_AB_, r_AC_, r_AD_, r_AE_, r_BC_, r_BD_, r_BE_, r_CD_, r_CE_, and r_DE_, where Y is an index for vectors of sample mRNA expression levels and A, B, C, D and E are indices for vectors of population genotypes of SNP_A_, SNP_B_, SNP_C_, SNP_D_, and SNP_E_, respectively. The values of these variables differed with each independent selection of SNP_A_, SNP_B_, SNP_C_, SNP_D_, and SNP_E_.

For 1-iSNP models, an independent calculation of R^2^_A_ = R^2^_B_r^2^_AB_ (Fig. 1) was performed for each ROI SNP for each choice of SNP_B_ selected from a list of ROI SNPs. Each choice of SNP_B_ represents a specific 1-iSNP model. To reduce computation burden, candidate iSNPs (SNP_B_) were selected from a shortened list of ROI SNPs from which SNPs with duplicate genotypes and SNPs that fail to meet a specific P-values threshold (e.g., P < 0.05) had been removed. To identify the best 1-iSNP model generated by this process, the normalized root mean-square error (NRMSE) was calculated for each model by comparing the estimated and predicted R^2^_A_ values for all of the SNPs in the ROI, with the models ranked in order of decreasing 1/NRMSE. The quality of fit of each model was also assessed by calculating adjusted R^2^_model_ values based on linear regression analysis of estimated R^2^_A_ vs. predicted R^2^_A_.

Generation of 2-iSNP models based on the equation R^2^_A_ = [R_B_(r_AB_ − r_BC_r_AC_) + R_C_(r_AC_ − r_AB_r_BC_)]^2^/(1 − r_BC_^2^)^2^ (Fig. 1) were carried out as described above, with all possible combinations of two candidate iSNPs (SNP_B_ and SNP_C_) chosen from the reduced list of ROI SNPs. Again, models were ranked in order of decreasing 1/NRMSE and quality assessed by calculating adjusted R^2^_model_. Due to the large number of terms in the polynomial equations defining R^2^_A_ in 3- and 4-iSNP models, R^2^_A_ values in these models were calculated based on our derived equation, R^2^_A_ = −β/2α, using values of the quadratic equation-related terms -β and 2α listed in Table 1. This simple expression for R^2^_A_, which we believe holds for models containing an arbitrary number of iSNPs, is based on our observation that solutions of quadratic equations required to solve constraint matrix equation-derived polynomial equations for R_A_: R_A_ = −β ± (β^2^ − 4αγ)^1/2^/2α, simplify to R_A_ = −β/2α, because the terms under the square-root sign of the equation sum to 0 under the given constraints. (See Supplementary File 3 for details concerning these calculations.) Ranking of models and quality assessment was as described above. To date, a maximum of 3-iSNPs has sufficed for the analysis of most experimental data sets.

**Figure 2:**
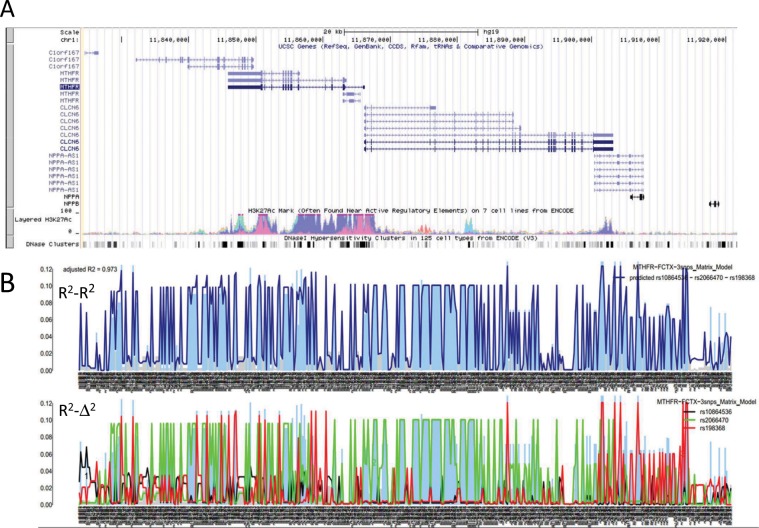
Multiple coefficient of determination-based analysis of human *MTHFR* mRNA expression in frontal temporal cortex (FCTX). (**A**) Screen shot from the USCS Genome Browser (GCH37/hg19 version) showing the chromosome 1 ROI containing *MTHFR* and neighboring genes. The two tracks at the bottom of this panel show: (i) levels of histone H3-lysine 27 acetylation (H3K27Ac), a marker for open, transcriptionally active chromatin detected in multiple cell lines, and (ii) clusters of DNase I-sensitive sites (DNase clusters), which are also markers for open chromatin. (**B**) Upper graph: a “R^2^-R^2^” plot comparing estimated values for coefficients of correlation (R^2^) derived from single-variable linear regression analyses of correlations between mRNA expression levels and genotypes for 100 genotyped and 298 imputed SNPs in the chromosome ROI [blue (nominal P < 0.05) and grey (nominal P ≥ 0.05) bars] and predicted R^2^ values calculated as described in the text. Lower graph: a “R^2^− Δ^2^” plot showing the parsing of ROI SNPs into three semi-independent families, each comprising a subset of ROI SNPs that are in LD with one of three index SNP (iSNP) selected as described in the text. The three iSNPs listed on the upper-right corners of the two plots were selected as the combination of SNPs that produced the closest agreement (smallest NRMSE) between estimated and predicted R^2^ values among thousands of randomly select combinations of three ROI SNPs. The adjusted R^2^_model_ (adjusted R2) listed on the top left of the upper plot, was derived from linear regression analysis of the correlation between estimated and predicted R^2^-values and provides a measure of the “goodness-of-fit” for this combination of iSNPs. (See Supplementary File 2. Online Methods for details concerning imputation of SNP genotypes).

Figure 2 shows the results of analysis of human *MTHFR* mRNA expression in frontal cortex based on mRNA expression and genotype data from the 4BrainR^18^ data set (n = 144 Caucasian brain samples; 398 genotyped or imputed SNPs within an ~100 kb chromosome ROI containing the *MTHFR* gene: Fig. 2A). The results shown are for the 3-iSNP model selected on the basis of lowest NRMSE among the top 2200 models generated in the analysis.

The upper graph in Fig. 2B is a “R^2^-R^2^ plot” comparing: (i) “estimated’ R^2^ values derived from single-variable linear regression analyses of the mRNA expression/genotype data calculated independently for each SNP (blue bars and gray) and (ii) “predicted” values of R^2^ obtained from matrix equation-based calculations (dark blue line). The quality of the fit between the estimated R^2^_A_ values (blue/gray bars) and predicted R^2^_A_ values (dark blue line) is obvious upon inspection of the upper (R^2^-R^2^) plot and quantified by adjusted R^2^_model_ = 0.973. The lower graph in Fig. 2B is a “R^2^-Δ^2^ plot,” where values of pairwise r^2^ (=Δ^2^) LD coefficients calculated for each SNP with respect to SNP_B_ (red line), SNP_C_ (green line), SNP_D_ (black line) are imposed on the estimated R^2^ value for each SNP (blue bars). To facilitate comparisons, the heights of the red, green and black lines were scaled to the heights of the estimated R^2^ value for each iSNP. Together, these lines define three semi-independent families of SNPs linked to the iSNPs: rs198368 (red), rs2066470 (green), and rs10864536 (black). Independent analyses of 4BrainR data for *MTHFR* mRNA expression in fontal cortex (FCTX), temporal cortex (TCTX), cerebellum (CERE) and pons (PONS) show that FCTX, TCTX and CERE share the same three red, green and black iSNP families, while PONS is best modeled by 3 iSNPs drawn from the rs10864536 iSNP family (Supplementary File 6). Thus, although R^2^ values for individual SNPs usually differ between datasets, iSNP families are often conserved.

This was also true for *MTHFR* mRNA expression in independent sets of lymphoblastoid cell lines (LCLs) derived from Japanese (JPT), Chinese (CHB) or Caucasian (CEU) populations (Supplementary File 7). In this case, three iSNP families were identified in the CEU-LCLs. By contrast, analysis of the JPT- and CHB-LCLs parsed the ROI SNPs into two iSNP families, with one of the families comprising two the iSNP families detected in the CEU-LCLs. The different results obtained for JPT/CHB-LCLs compared to CUE-LCLs reflect differences in the LD structure of these populations, and provide a good example of how, on one hand, a single iSNP can harbor more than a single regulatory variant and, on other hand, how analysis of mRNA expression of the same gene in different populations can reveal the presence of “hidden” regulatory variants.

Comparison of the SNP lists obtained for the 3-iSNP families observed in the CEU-LCLs with those obtained for FCTX revealed one of the families (iSNP = rs2274976) to correspond to the FTCX green iSNP family and the other two iSNP families (iSNP = rs4845881 and re1023252) to subsets of the FCTX black IiSNP family of SNPs. Together, these results suggest that regulatory variants associated with the green and black iSNP families contribute to mRNA expression in FCTX, TCTX, CERE and LCLs, while regulatory variants associated with the FCTX red iSNP family are active in FCTX, TCTX and CERE, but not in PONS or LCLs. The observation that FCTX black iSNP family SNPs are parsed into two separate families in LCLs suggest that this family contains at least two distinct regulatory variants. Obviously, more work will be required to elucidate the number and locations of regulatory variants associated with each SNP family, but we believe that the above example illustrates the usefulness of our method for fine-structure analysis of mRNA expression and its power to produce hypotheses for further investigations.

A summary of the above findings is provided in Supplementary File 8, Tables S1 and S2. The analysis of four additional genes: the well-studied gene *CHI3L2* (*Chitinase 3 like 2*), the autism and schizophrenia candidate gene *DGCR8* (*DiGeorge Critical Region-8*) and the Alzheimer candidate genes *GSTM3* and *GSTM5* (*Glutathione S-transferase mu-1/5*) can be found in Supplementary Files 9–11.

## Discussion

The method for analyzing mRNA expression data described in the paper provides a nearly complete accounting for ROI SNPs for genes under investigation and an easy way to visualize iSNP families, facilitating the detection of iSNP families that are conserved between different tissues in the same set of individuals or between individuals in different populations. The method is flexible, allowing the selection of iSNP families based upon: (i) minimum NRMSE, (ii) maximum adjusted R^2^_model_, (iii) maximum R^2^_M_, (iv) maximum sum of estimated R^2^ values for individual iSNPs, (v) minimum Akaike information criterion (AIC) and/or (vi) minimum Bayesian Information criterion (BIC). Minimum NRMSE is set as the default criterion. Importantly, the method also provides a quantitative account of how regulatory variants influence the estimated single-variable linear regression R^2^ values (R^2^_A_) for non-regulatory SNPs in linkage disequilibrium within datasets under investigation. Specifically, it clearly shows how the combined effects of multiple regulatory variants can only be understood through application of the “constraint” matrix equations defined in this study, i.e., R^2^_AB_ = R^2^_B_, R^2^_ABC_ = R^2^_BC_, R^2^_ABCD_ = R^2^_BCD_, etc., for which we derived a general formula (Table 1). These combined effects are often counter-intuitive, dramatically inflating or deflating R^2^_A_ values expected from the simple sum of (r^2^_AX_)(R^2^_X_) terms, where x = B, C, D or E (Supplementary File 3). These “unexpected” results are produced by the many terms within the polynomial solutions of “constraint” matrix equations that contain Pearson correlation coefficients, which can take on positive or negative values. The method also provides estimates of the minimum number of regulatory variants within the chromosome ROI that contribute to mRNA expression and, by defining specific iSNP families, provides hints concerning the locations of those variants.

Limitations of the current method include the current lack of hybridization array-based mRNA expression data sets that produce reproducible results for many genes of interest and the subjective nature of choosing the best iSNP models. Although we have not yet used this method to analyze RNA-sequencing-based mRNA expression data, we are optimistic that these datasets will provide better replication compared to array-based datasets. Likewise, we believe that comparing the results of models selected based on different criteria, for example maximum 1/NRMSE versus maximum R^2^_M_, may produce insights not available from consideration of models based on a single criterion. A discussion of two additional limitations of our method, the assumption of additive effects of SNP alleles and applicability primarily to common, cis-acting, biallelic genetic variants, can be found in Supplementary File 2.

Finally, we want to stress that our method only quantifies associations among SNPs within the datasets under investigation, rather than identifying specific regulatory variants. Thus, caution must be taken when interpreting the results of analyses. The identification of an iSNP family does not necessarily guarantee the existence of underlying regulatory variants: our program will dutifully parse statistical noise as well as true causal relationships SNP. For all of these reasons, we have made no effort to provide P-values for selected models. Rather, we hope that this method will be useful for bringing order to the “forest” of SNPs surrounding genes of interest and for generating hypothesis that can be investigated using additional bioinformatic tools and by experimentation.

## Methods

Briefly, we developed a mathematical method for parsing SNPs that correlate with mRNA expression into SNP families related by LD using a matrix equation from multivariate statistical analysis that allows the calculation of multiple coefficients of determination (R^2^_M_) based on Pearson correlation coefficients (r_YX_) between: 1) a dependent variable, (Y = mRNA expression) and multiple independent variables (X = SNP genotypes) and ii) pairwise comparisons of the independent variables. We subsequently developed a R language-based computer program for carrying out the required calculations and plotting the results of the analysis of simulated and experimental mRNA expression/SNP genotype data sets. A detailed description of the methods developed in this study can be found in Supplementary Files 2–4 available online. Copies of our R-programs for matrix equation calculations and data analysis are available online at: https://github.com/saffenlab/R2-D2-model.

## Supplementary information

Supplementary Information

## Data Availability

The datasets generated during the current study are available from the corresponding author on reasonable request.
