# Supplementary material for: A multiple coefficient of determination-based method for parsing SNPs that correlate with mRNA expression
Source: Sci Rep. 2019 Dec 27;9:20110. doi: 10.1038/s41598-019-56494-9 (PMC6934451; doi:10.1038/s41598-019-56494-9)
Supplement: Supplementary file 1 — Supplementary Information [file 41598_2019_56494_MOESM1_ESM.pdf]

A multiple coefficient of determination-based method for parsing SNPs that correlate with mRNA expression

Fan Song[1], Yu Tao[1], Yue Sun[2,4] and David Saffen[1,2,3]*

1. Department of Cellular and Genetic Medicine, School of Basic Medical Sciences, Fudan University, Shanghai 200032, China.
2. Institutes of Brain Science, Fudan University, Shanghai 200032, China.
3. State Key Laboratory for Medical Neurobiology, Fudan University, Shanghai 200032, China.
4. School of Life Sciences, Fudan University, Shanghai 200438, China.

*Correspondence to: David Saffen, Department of Cellular and Genetic Medicine, School of Basic Medical Sciences, Fudan University, 130 Dong'an Rd, Building 13, Room 229, Shanghai 200032, China. Email: saffen@fudan.edu.cn.

Supplementary Information

Supplementary File 1. Mathematical Notation
Supplementary File 2. Online methods
Supplementary File 3. Derivation of equations
Supplementary File 4. Simulation of mRNA expression/SNP genotype datasets
Supplementary File 5. Figures S1 and S2
Supplementary File 6. *MTHFR* mRNA expression in FCTX, TCTX, CERE and PONS
Supplementary File 7. *MTHFR* mRNA expression in JPT-, CHB- and CEU-LCLs
Supplementary File 8. Tables S1 and S2
Supplementary File 9. *CHI3L2* expression in CEU-LCLs and TCTX
Supplementary File 10. *DGCR8* expression in 4BrainR and BC data sets
Supplementary File 11. *GSTM5-GSTM3* expression in 4BrainR and BC data sets

Supplementary File 1. Mathematical Notation

Symbol/Equation	Roles in simulated (S) and/or experimental (E) mRNA expression/genotype datasets
SNP_A	Non-regulatory genetic variant
SNP_B, SNP_C, SNP_D, SNP_E	Regulatory genetic variants (S) or iSNPs (E); iSNP = "index" SNP
Δ^2_{AB}, Δ^2_{BC}, etc.	"Estimated" pairwise r^2 linkage disequilibrium (LD) coefficients for the indicated SNPs, based on genotypes
R^2_A, R^2_B, R^2_C, R^2_D, R^2_E	Coefficients of determination for individual SNPs from single-variable linear regression analysis of mRNA expression versus SNP genotypes
R^2_{AB}, R^2_{ABC}, R^2_{ABCD}, R^2_{ABCDE}	Multiple coefficients of determination for the indicated combinations of SNPs
$R^2_M = \mathbf{C}^T\mathbf{R}^{-1}\mathbf{C}$	Matrix equation defining sample multiple (M) coefficient of determination, where **C** = vector of Pearson correlation coefficients for mRNA expression versus SNP genotypes; **R** = correlation matrix for SNP genotypes, with elements = pairwise Pearson correlation coefficients; T = transpose; $\mathbf{R}^{-1}$ = inverse of **R**
$\mathbf{R}^{-1} = \mathbf{adR}/\mathbf{detR}$	Equation for calculating inverse **R**, where adj**R** = adjugate of **R** and det**R** = determinant of **R**
$R^2_{AB} = R^2_A$, $R^2_{ABC} = R^2_{BC}$, etc.	"Constrained" matrix equations
**"estimated" R^2_A**	Values of R^2_A calculated from single variable linear regression analysis of mRNA expression versus SNP_A genotypes for the sample under investigation
**"predicted" R^2_A**	Values of R^2_A calculated using "constrained" matrix equations for the sample under investigation
**adj R^2_{model}**	R^2 from linear regression analysis of "estimated" versus "predicted" R^2_A values, adjusted for number of iSNPs in matrix equation model
**"R^2-R^2 plot"**	Graph of "estimated" R^2_A values (vertical bars) versus "predicted" R^2_A values (blue or red line) for SNPs in a chromosome region of interest
**"R^2-D^2 plot"**	Graphs of "estimated" R^2_A values (vertical bars) and pairwise D^2 (= r^2) LD coefficients with respect to one or more iSNPs for SNPs in a chromosome region of interest, scaled to the heights of iSNP "estimated" R^2_A values.

Supplementary File 2. Online Methods

The method that we describe in this study is based on the hypothesis that values of R^2_A are "constrained" by the requirement to satisfy one of the following equalities: ($R^2_{AB} = R^2_B$), ($R^2_{ABC} = R^2_{BC}$), ($R^2_{ABCD} = R^2_{BCD}$) or ($R^2_{ABCDE} = R^2_{BCDE}$), where A is the index for a non-regulatory variant and B,C,D,E are indices for regulatory variants. These equations reflect our hypothesis that genetic variants that do not directly contribute to mRNA expression, but nevertheless correlate with mRNA due to being in LD with one or more regulator variant, do not contribute to the variance of mRNA expression over-and-above the variance explained by the regulatory variants.

The next steps in the development of our method were to: 1) replace the coefficients of determination in the above equalities with their corresponding matrix expressions ($\mathbf{C}^T\mathbf{R}^{-1}\mathbf{C}$), 2) expand the matrix expressions on both sides of the equal sign to obtain their polynomial equivalents, and 3) manually solve (for up to 4-variant systems) the resulting polynomial equation for R_A and square this term to obtain R^2_A. (See **Supplementary File 3** for details.)

When using the above matrix equations to analyze simulated or experimental mRNA expression/ genotype data, each SNP within the chromosome region of interest (ROI) is sequentially considered to be a candidate non-regulatory variant (SNP_A) and its R^2_A value calculated for random combinations of candidate regulatory variants (or "index" SNPs proposed to be in high LD with regulatory variants: $iSNP_B$, $iSNP_C$, $iSNP_D$, $iSNP_E$), depending upon the predetermined number of regulatory variants in the model. Each combination of candidate regulatory variants/iSNPs represents a distinct model. Criteria for selecting the best model among the many thousands generated by these calculations include: i) minimum total normalized root mean squared error (NRMSE) for differences between "estimated" R^2 values derived from single variable linear regression analysis of mRNA expression versus SNP genotypes for each ROI SNP and "predicted" R^2 values derived from matrix equation calculations for each ROI SNP, ii) maximum adjusted R^2_{model} value derived from linear regression analysis of "estimated" versus "predicted" R^2 values for ROI SNPs, iii) maximum R^2_M value calculated using the appropriate $R_M{}^2 = \mathbf{C}^T\mathbf{R}^{-1}\mathbf{C}$ matrix equation, iv) maximum sum of the R^2 value for individual candidate regulatory variant/iSNPs, v) minimum Akaike information criterion (AIC) and/or vi) minimum Bayesian Information criterion (BIC). Minimum NRMSE is set as the default criterion.

Additional details concerning our method and its applications are provided in the following sections.

Coefficients of Determination (R^2) vs. SNP rs number (R^2-SNP) plots

Single-variable linear regression analyses of mRNA expression versus genotypes (coded 0, 1, or 2, based on the number of minor alleles) for SNPs in chromosomal ROI were carried out using the --assoc command in PLINK [1] with mRNA expression and SNP genotype data from simulated or experimental datasets (Ref: "BrainCloud" [2] ,"4BrainR" [3] and lymphoblastoid cell lines [4].) For each SNP, this analysis yielded: i) a regression coefficient (a measure of the allelic effect size), ii) a coefficient of determination [R^2] (a measure of the contribution of each SNP to the variance of mRNA expression) and iii) a nominal P value (a measure of statistical significance) and iv) chromosome location in GRCh37/hg19

coordinates. To better understand positional relationships among SNPs that correlate with mRNA expression for individual genes, we constructed bar graphs with the "estimated" R^2 value represented by the height of each bar (y-axis) positioned over the rs identification number listed on the X-axis, with SNPs listed in their order of occurrence within the chromosome ROI. The color of each bar was assigned based on the nominal (i.e., uncorrected for multiple testing) P-value obtained from the linear regression analysis for the corresponding SNP: blue for $P < 0.05$ and grey for $P \geq 0.05$.

Matrix equation for calculating multiple coefficients of determination (R_M^2)

As explained in detail in the Results section of the main text, the multiple coefficient of determination (R_M^2), representing the combined contributions of two or more biallelic regulatory variants (in simulations) or index SNPs (in the analysis of experimental data), was defined by the matrix equation: $R_M^2 = \mathbf{C}^T\mathbf{R}^{-1}\mathbf{C}$, where **C** is a vector comprising Pearson correlation coefficients (r_{YG}) between mRNA expression (Y) and genotypes (G) for each regulatory variant or regulatory variant proxy SNP included in the analysis, and $\mathbf{C}^T$ is its transposed vector. **R** is the correlation matrix comprising Pearson correlation coefficients for all pairwise combinations of genotypes (r_{GiGj}) for all regulatory variants or proxy SNPs and $\mathbf{R}^{-1}$ is the inverse of **R**. [5]. To simplify our notation, we designated non-regulatory variants as SNP_A and distinct biallelic regulatory variants or proxy SNPs as SNP_B, SNP_C, SNP_D or SNP_E. Each proxy SNP [termed an "index" SNP (iSNP) in our notation] was hypothesized to be a regulatory variant or a SNP in high linkage disequilibrium (LD) with one or more regulatory variants.

Matrix-based method for analyzing the contributions of SNPs to mRNA expression

Our method is based on the hypothesis that the coefficient of determination for the association between a non-index SNP_A and mRNA expression, $R^2_A = (r_{YA})^2$, can be calculated by deriving equations for R^2_A that satisfy the following constraints: $R^2_{AB} = R^2_B$ (for one iSNP = SNP_B), $R^2_{ABC} = R^2_{BC}$, (or two iSNPs = SNP_B and SNP_C), $R^2_{ABCD} = R^2_{BCD}$, (for three iSNPs = SNP_B, SNP_C and SNP_D), $R^2_{ABCDE} = R^2_{BCDE}$, (for four iSNPs = SNP_B, SNP_C, SNP_D and SNP_E), where R^2_{AB}, R^2_{ABC}, R^2_{BC}, R^2_{ABCD}, R^2_{BCD}, R^2_{ABCDE}, and R^2_{BCDE} represent sample multiple coefficients of multiple determination, R^2_M, for the indicted combinations of SNPs. In other words, we derived equations for calculating R^2_A that account for the correlation of SNP_A with mRNA expression exclusively in terms of the: i) contributions of one or more regulatory variants or index SNPs (SNP_B, SNP_C, SNP_D, SNP_E) to mRNA expression of the gene of interest ($R^2_B = r^2_{YB}$, $R^2_C = r^2_{YC}$, $R^2_D = r^2_{YD}$, $R^2_E = r^2_{YE}$) , ii) pairwise r^2 LD coefficients between these variants or iSNPs (r^2_{BC}, r^2_{BD},r^2_{CD}, etc.) , and iii) the r^2 LD coefficient between SNP_A and each of regulatory variant or iSNP (r^2_{AB}, r^2_{AC}, r^2_{AD}, r^2_{AE}). As described in the main text and **Supplementary File 2**, manually solving the relevant matrix equations for systems containing 1,2 or 3 iSNPs under the above constraints and generalizing these results to systems containing higher numbers of iSNPs yielded the expressions for R_A^2 listed in **Table 1** in the main text.

Simulation of mRNA expression/genotype data sets

For each regulatory variant (SNP_B, SNP_C, SNP_D) in a model, mRNA expression values were assigned using Fisher's method of "genetic means" [6]. Error terms were simulated by adding values randomly selected from a normal distribution of values with mean = 0 and variance = fV_A: $N_0[0, fV_A]$ to

the total genotypic value of each individual in the sample (f = 1/20, 1/10, 1/5, etc.; V_A = total additive variance of assigned mRNA expression values in the sample). (See **Supplementary File 3** for details.)

Method 1. Genotypes for small sets of SNPs [usually, one non-regulatory SNP (designated SNP_A), plus one or more biallelic regulatory variants (designated SNP_B, SNP_C, SNP_D, SNP_E) were generated by randomly assigning frequencies to sets of all possible haplotypes (i.e. four haplotypes for 2 SNPs, eight for 3 SNPs, sixteen for 4 SNPs, etc.) in such a way that the frequencies in each set sum to 1. (See **Supplementary File 3** for details.)] Diplotype frequencies were calculated for all pairwise combinations of these haplotypes: 16 for 2 SNPs, 64 for 3 three SNPs, 256 for 4 SNPs, etc. After summing frequencies for duplicate diplotypes (yielding, for example, 1+2+ …+16 = (16)(17)/2 = 136 unique diplotypes in a four-SNP system), diplotype frequencies were multiplied by the number of individuals to be included in the dataset (e.g., n = 1000), yielding the number individuals with each unique diplotype to be represented in the dataset. To facilitate the creation of data sets containing a large number of non-regulatory SNPs, a procedure was developed to round off the number of individuals for each unique diplotype in such a way as to maintain the preset sample size = n. (See **Supplementary File 3** for details.) Finally, diplotypes for each individual in the sample were converted to genotypes, and coded 0, 1, 2 to represent homozygous major allele, heterozygous, homozygous minor allele, respectively.

Method 2. For simulations including one or two regulatory variants, frequencies of haplotypes comprising SNP alleles were calculated using the polynomial equations listed in **Supplementary File 3**, **section D**. SNP genotypes for each individual in the simulated dataset (typically n = 1000) were derived from these haplotype frequencies as described above, using sequentially increasing values of minor allele frequencies and pairwise r^2 LD coefficients as input variables.

Goodness of fit for 1, 2, 3 or 4 regulatory variant models containing increasing levels of simulated experimental error was assessed by: i) calculating the normalized root mean squared error (NRMSE) for differences between “estimated” R_A^2 values and “predicted” R_A^2 values based on the “constraint” matrix equations” described above, and ii) calculating the adjusted R_{model}^2 based on linear regression analysis of correlations between “estimated” R_A^2 values and these “predicted” R_A^2 values.

Analysis of lymphoblastoid cell lines (LCLs) and brain mRNA expression data

1) mRNA expression data for 54 lymphoblastoid cell lines of Caucasian population origin were downloaded from the International HapMap Project database (GEO session number GSE2552) and genotypes for these 54 individuals were obtained from HapMap Phase 3 r2 [ftp://ftp.ncbi.nlm.nih.gov/hapmap/genotypes/2009-01_phaseIII/hapmap_format/consensus/].

2) mRNA expression and genotype data from two Caucasian population-based brain mRNA expression studies: i) “BrainCloud”: Colantuoni, C et al. [2] (DLPFC: dorsolateral prefrontal cortex from 109 independent brains) and ii) “4BrainR”: Gibbs, J et al. [3] (FCTX: frontal cortex, TCTX: temporal cortex, CERE: cerebellum, PONS: pons from 145 independent brains) were downloaded from the Gene Expression Omnibus (GEO; http://www.ncbi.nlm.nih.gov/geo/, accession numbers GSE30272 and GSE15745) and the database of Genotypes and Phenotypes (dbGaP: accession number phs000417.v1.p10. and phs000249.v1.p1).

Genotype imputation

Genome-wide imputation of genotypes for the i) the BrainCloud DLPFC and ii) 4BrainR (FCTX and TCTX) datasets and region-wide imputation for the *CHI3L2* ROI in lymphoblastoid cell line dataset were carried out using SHAPEIT v2.r644 [7] and IMPUTE2 v2.3.0 [8] in combination with a 1000 Genomes Project genotype reference panel (phase1_v3.20101123) [9] downloaded from https://mathgen.stats.ox.ac.uk/impute/impute_v2.html#reference.

Linkage disequilibrium analysis

Pairwise r^2 LD coefficients for SNPs within each gene ROI were calculated and visualized using Haploview (http://www.broadinstitute.org/scientific-community/science/programs/medical-and-population-genetics/haploview/haploview) [10] or calculated by squaring values of Pearson correlations coefficients (r_{GiGj}) obtained from pairwise comparisons of SNP genotypes (G_i and G_j): $\Delta^2_{ij} = r^2_{ij} = (r_{GiGj})^2$. Scripts for calculating correlation coefficient matrices (rGG) and r^2 matrices (r^2GG) for SNPs in chromosome ROI are included in our custom R-language program developed to carry out the matrix equation-based analyses described in this paper.

Identification of sets of index SNPs that best account for estimated R^2_A values for SNPs within a ROI for simulated or experimental mRNA expression/genotype data sets.

1. Data quality control (experimental data sets). Multiple linear regression was used to estimate the contributions of covariates to raw mRNA expression values. The residuals from this procedure were designated "mRNA expression values" and used in association analyses. mRNA expression values in the "BrainCloud" dataset were corrected for five covariates: age, sex, batch number, sample source, RNA integrity number (RIN) and mRNA expression values in the "4BrainR" dataset were corrected for four covariates: age, sex, batch number, post-mortem interval (PMI). Following genotype imputation, SNPs with "info scores" < 0.3 and MAFs < 0.01 (1%) were removed. Individuals with genotype missing rate higher than 0.1 (10%) were also removed for subsequent analysis. Genotypes of retained SNPs were coded 0, 1, or 2, based on the number of minor alleles using the –recodeA command in PLINK.

2. Pearson correlation coefficients (r_{YG}) between mRNA expression levels (Y) and genotypes (G) for each SNP in the ROI were calculated using the R function cor(). For each SNP, the squared value of this correlation coefficient was designated the "estimated" coefficient of determination, i.e., $(r_{YGA})^2 = R^2_A$.

3. Calculation of Pearson Correlation coefficients (r_{GiGj}) between sample genotypes for all pairwise combinations of SNPs (G_i x G_j) in the ROI were carried out in R as described above. Pearson correlation coefficients obtained in steps 2 and 3 are used as inputs for matrix calculations. As mentioned above, the value of the squared Pearson correlation coefficient for two SNPs is an estimator for the r^2 LD coefficient of the two SNPs.

4. Optimal sets of index SNPs for each gene were identified as follows: R^2_A values for 2-, 3-, and 4-SNP systems were calculated using the equations listed in Figure 1 or Table 1 in the main text. Goodness-of-fit was examined by calculating the normalized root mean squared error (NRMSE) for SNP-wise comparisons between: i) "estimated" R^2_A values obtained from single-variable linear regression analysis of mRNA expression versus SNP genotype as described above and ii) "predicted" R^2_A-values calculated using the matrix equation with independent calculation performed for each SNP. When

analyzing simulated or experimental data sets, the usual procedure was to sequentially test 1-, 2- , or 3-index SNP models, proceeding until: i) all of the SNPs in the ROI with $P < 0.05$ were accounted for and/or ii) the values of NRMSE failed to decrease. To facilitate comparisons between analyses of the same gene using independent mRNA expression/genotype datasets or different genes within a single data set, the "goodness-of-fit" for iSNP combinations was also assessed by calculating the adjusted R^2_{model} based on linear regression analysis of "estimated" versus predicted R^2_A values.

5. To visualize the results of the above procedures, we constructed i) R^2-R^2 plots comparing "estimated" and "predicted" R^2_A values and ii) R^2-Δ^2 plots showing semi-independent iSNP families. R^2-R^2 plots are the same as the "estimated" R^2-SNP plots described above, with the addition of a (dark blue) line plotting "predicted" R_A^2 values calculated from the matrix equations super-imposed on the blue and grey bars representing "estimated" R_A^2 values. R^2-Δ^2 plots (aka "iSNP family" plots, with $\Delta^2 = r^2$ LD coefficient) comprise R^2-SNP plots with super-imposed lines representing the pairwise r^2 values between each SNP and one or more index SNPs, with lines of different color representing different iSNP "families." To approximate the contributions to the variance of mRNA expression made by individual SNPs, the height of the lines was scaled to the R^2 values of the index SNP for each SNP family. R^2-R^2 and R^2-Δ^2 plots were constructed using custom R scripts.

6. Refined definition of SNP families: Comparisons of iSNP families identified for the same gene in different data sets, were facilitated by setting minimum r^2 thresholds for inclusion. SNPs were initially assigned to a specific iSNP "family" when the r^2 LD coefficient between the SNP and iSNP was larger than the r^2 LD coefficient for other iSNPs. Depending upon the gene under analysis, lower-end thresholds for inclusion in a specific iSNP family were then typically set at r^2= 0.6 or 0.4.

<u>Limitations of our matrix-equation-based, multiple coefficient of determination method</u>

1. As mentioned in the Discussion section of the main text, the most important limitation that we have encountered is the lack of high-quality mRNA expression/SNP genotype datasets that show reproducible patterns of mRNA expression for most genes. We believe that this lack of reproducibility is related to the relative insensitivity and non-reproducibility of array-based mRNA assays. We hope that the availability of larger, high-quality RNA sequencing (RNA-seq)–based studies with provide more accurate and reproducible mRNA expression data in the future.

2. Our method is based on the assumption that alleles of individual genetic variants, as well as combinations of genetic variants, function independently to produce additive contributions to mRNA expression, an assumption common to most studies of genetic variation of mRNA expression in humans. The validity of this assumption is supported by a study of mRNA expression in human blood cells based upon pedigree and SNP analyses that determined that > 94% of ~ 18 thousand mRNA probes showed additive genetic variation, with dominance and over-dominance effects accounting for a fraction of the non-non additive genetic variation [11]. Nevertheless, the assumption of additive genetic effects for SNP alleles in our models represents a potential limitation of our method, since it does not explicitly address possible recessive, dominant, over-dominant, or co-dominant effects on mRNA expression.

3. Our method is particularly applicable to the analysis of common, cis-acting, bi-allelic regulatory variants. Mathematically, the multiple coefficient of determination (R^2_M) is invariant when calculated using standardized mRNA expression and genotype correlation data. This property suggests that our matrix equation-based method could also be used to analyze the contributions of rare variant to mRNA expression. In the relatively small (54-145 independent samples) experimental data sets that we have analyzed, however, moderately rare variants only infrequently appear and are often not reproducible among independently data sets. Moreover, very rare variants are excluded from consideration, due to the removal of SNPs with frequencies less than 1% as part of our quality control procedures for analyzing experimental data, described above. The above-mentioned property of R^2_M is likely to become more important when and if much larger, high-quality mRNA expression data sets become available for analysis.

References

[1] S. Purcell, B. Neale, K. Todd-Brown, L. Thomas, M.A.R. Ferreira, D. Bender, J. Maller, P. Sklar, P.I.W. de Bakker, M.J. Daly, P.C. Sham, PLINK: a tool set for whole-genome association and population-based linkage analyses., Am. J. Hum. Genet. (2007). doi:10.1086/519795.

[2] C. Colantuoni, B.K. Lipska, T. Ye, T.M. Hyde, R. Tao, J.T. Leek, E.A. Colantuoni, A.G. Elkahloun, M.M. Herman, D.R. Weinberger, J.E. Kleinman, Temporal dynamics and genetic control of transcription in the human prefrontal cortex, Nature. (2011). doi:10.1038/nature10524.

[3] J.R. Gibbs, M.P. van der Brug, D.G. Hernandez, B.J. Traynor, M.A. Nalls, S.L. Lai, S. Arepalli, A. Dillman, I.P. Rafferty, J. Troncoso, R. Johnson, H.R. Zielke, L. Ferrucci, D.L. Longo, M.R. Cookson, A.B. Singleton, Abundant quantitative trait loci exist for DNA methylation and gene expression in Human Brain, PLoS Genet. (2010). doi:10.1371/journal.pgen.1000952.

[4] V.G. Cheung, R.S. Spielman, K.G. Ewens, T.M. Weber, M. Morley, J.T. Burdick, Mapping determinants of human gene expression by regional and genome-wide association, Nature. (2005). doi:10.1038/nature04244.

[5] R.A. Johnson, D.W. Wichern, Applied multivariate statistical analysis (6th Edition). Prentice Hall, Englewood Cliffs, NJ. (1992).

[6] Jeffrey K. Conner and Daniel L. Hartl, A Primer of Ecological Genetics, Sinauer Associates, Sunderland (Massachusetts), (2005).

[7] O. Delaneau, J.-F. Zagury, J. Marchini, Improved whole-chromosome phasing for disease and population genetic studies., Nat. Methods. (2013). doi:10.1038/nmeth.2307.

[8] B. Howie, C. Fuchsberger, M. Stephens, J. Marchini, G.R. Abecasis, Fast and accurate genotype imputation in genome-wide association studies through pre-phasing, Nat. Genet. (2012). doi:10.1038/ng.2354.

[9] 1000 Genomes Project Consortium, An integrated map of genetic variation, Nature. (2012). doi:10.1038/nature11632.

[10] J.C. Barrett, B. Fry, J. Maller, M.J. Daly, Haploview: Analysis and visualization of LD and haplotype maps, Bioinformatics. (2005). doi:10.1093/bioinformatics/bth457.

[11] Powell JE, Henders AK, McRae AF, Kim J, Hemani G, Martin NG, Dermitzakis ET, Gibson G, Montgomery GW and Visscher PM, Congruence of additive and non-additive effects on gene expression estimated from pedigree and SNP data, PLOS Genet. 9(5), (2013) e1003502.

Supplementary File 3.
Derivation of equations used to calculate the contributions of one, two or three regulatory variants to the R^2 of a non-regulatory variant.

The starting point for our derivations was the matrix equation that defines the multiple coefficient of determination: $R^2_M = \mathbf{C}^T\mathbf{R}^{-1}\mathbf{C}$. The components of this equation are explained in the following boxes:

$R^2_M = \mathbf{C}^T\mathbf{R}^{-1}\mathbf{C}$

C = j x 1 vector comprising phenotype/SNP genotype correlations for j SNPs

R = j x j matrix comprising correlations between genotypes for all possible pairs of j SNPs in model

Elements of **C** and **R** are Pearson correlation coefficients

Phenotype (Y) = mRNA expression levels measured using microarrays, PCR-based assays, or RNA-seq

r_{YA} = Pearson correlation coefficient for mRNA expression values versus SNP_A genotypes

r_{AB} = Pearson correlation coefficient for pairwise comparison of the genotypes of SNP_A and SNP_B

Example (four-SNP system):

$\mathbf{C}^T = [r_{YA}, r_{YB}, r_{YC}, r_{YD}] = [A, B, C, D]$

$$\mathbf{R} = \begin{bmatrix} r_{AA} & r_{AB} & r_{AC} & r_{AD} \\ r_{BA} & r_{BB} & r_{BC} & r_{BD} \\ r_{CA} & r_{CB} & r_{CC} & r_{CD} \\ r_{DC} & r_{DB} & r_{DC} & r_{DD} \end{bmatrix} = \begin{bmatrix} 1 & a & c & f \\ a & 1 & b & d \\ c & b & 1 & e \\ f & d & e & 1 \end{bmatrix}$$

$\mathbf{R}^{-1}$ = adjugate **R/**determinant **R** = adj**R/**det**R**

$$\text{adj}\mathbf{R} = \begin{bmatrix} b_{11} & b_{12} & b_{13} & b_{14} \\ b_{21} & b_{22} & b_{23} & b_{24} \\ b_{31} & b_{32} & b_{33} & b_{34} \\ b_{41} & b_{42} & b_{43} & b_{44} \end{bmatrix}; \quad \text{adj}\mathbf{R}(1/\text{det}\mathbf{R})\mathbf{R} = 1$$

$R^2_M = R^2_{AB}$ = the combined contributions of SNP_A and SNP_B to the variance of mRNA expression

$R^2_M = R^2_{ABC}$ = the combined contributions of SNP_A, SNP_B and SNP_B to the variance of mRNA expression

$R^2_M = R^2_{ABCD}$ = the combined contributions of SNP_A, SNP_B, SNP_C SNP_D to the variance of mRNA expression

SNP_A = a bi-allelic non-"regulatory" SNP (non-rSNP) that correlates with mRNA expression only through linkage disequilibrium (LD) with one or more "regulatory" SNPs.

SNP_B, SNP_C and SNP_D = bi-allelic "regulatory" SNPs (rSNPs) that directly contribute to mRNA expression.

Note: when analyzing experimental data, SNP_B, SNP_C and SNP_D will denote biallelic regulatory variants or SNPs that are in high LD with unknown regulatory variants.

The goal of our derivations was to produce mathematical expressions for R^2_A in terms of the Pearson's correlation coefficients for phenotype (mRNA expression) versus genotype comparisons (r_{YG}) and pairwise comparisons of genotypes (r_{G1G2}) for all SNPs within two-, three- and four-SNP systems given the constraints. We hypothesize that this can be accomplished by determining the values of R^2_A that satisfy the following constraints:

$R^2_{AB} = R^2_B$	(one-rSNP systems)
$R^2_{ABC} = R^2_{BC}$	(two-rSNP systems)
$R^2_{ABCD} = R^2_{BCD}$	(three-rSNP systems)
$R^2_{ABCDE} = R^2_{BCDE}$	(four-rSNP systems)

Likewise for systems with larger numbers of rSNPs

In other words, we were looking for equations for R^2_A that allow R^2_M to be determined exclusively by one or more regulatory variants: SNP_B, SNP_C, SNP_D, SNP_E.

The derivation of an equation for R^2_A for a two-SNP system is provided in the following boxes:

One-rSNP systems (based on the constraint: $R^2_{AB} = R^2_B$)

$$R^2_{AB} = [A \quad B]\, \mathbf{R}^{-1} \begin{bmatrix} A \\ B \end{bmatrix} = R^2_B$$

$$\mathbf{R} = \begin{bmatrix} 1 & a \\ a & 1 \end{bmatrix}; \ \mathbf{R}^{-1} = \text{adj}\mathbf{R}/\text{det}\mathbf{R} = \begin{bmatrix} 1 & -a \\ -a & 1 \end{bmatrix} 1/(1- a^2)$$

Adj**R** = adjugate of **R** and det = determinant of **R**

$$R_{AB}{}^2 = [A \quad B] \begin{bmatrix} 1 & -a \\ -a & 1 \end{bmatrix} 1/(1- a^2) = \ [A - Ba \quad -Aa \ + B]\ 1/(1- a^2)$$

$$= (A^2 - 2ABa \ + B^2)/(1-a^2) = B^2$$

$$R_{AB}{}^2 (1-a^2) = (A^2 - 2ABa \ + B^2) = B^2(1-a^2) = B^2 - B^2a^2$$

$$\Rightarrow A^2 - 2ABa \ + B^2 = B^2 - B^2a^2$$

$$A^2 - 2ABa + B^2a^2 = 0, \ \alpha = 1, \beta = -2Ba, \ \gamma = B^2a^2$$

Using the quadratic equation to solve for A:

$$A = \frac{-\beta \pm \sqrt{\beta^2 - 4\alpha\gamma}}{2\alpha} = \frac{2Ba \pm \sqrt{(-2Ba)^2 - 4(B^2a^2)}}{2} = Ba = -\beta/2\alpha = -Bb_{12}/b_{11}$$

$\Rightarrow A^2 = B^2a^2 = \beta^2/4\alpha^2 = (Bb_{12})^2/(b_{11})^2$,
where $b_{11} = 1$ and $b_{12} = -a$ are elements of the adjugate matrix, adj**R**.

Double-checking by substituting $A = Ba$ and $A^2 = B^2a^2$:

$R^2_{AB}(1-a^2) - B^2 + B^2a^2 = B^2a^2 - 2(Ba)Ba + B^2 - B^2 + B^2a^2 = 0$

$R^2_{AB} = B^2 - B^2a^2/(1-a^2) = B^2(1-a^2)/(1-a^2) = B^2$

This implies that $R_{AB}^2 = B^2 = R_B^2$ (our original constraint).

Furthermore, $A^2 = B^2a^2 \Rightarrow (r_{YA})^2 = (r_{YB})^2(r_{AB})^2 \Rightarrow R_A^2 = R_B^2(r_{AB})^2$

$(r_{AB})^2 = r^2_{AB} = \Delta^2_{AB} =$ r^2 LD coefficient for SNP_A and SNP_B.

This yields the equation: $R^2_A = R^2_B\Delta^2_{AB}$

Together, these calculations demonstrate that the constraint $R^2_{AB} = R^2_B$ is satisfied when the contribution of the non-regulatory SNP_A to mRNA expression is proportional to the strength of its LD with the regulatory SNP, SNP_B.

Conversely: $\mathbf{R^2_{AB} = R^2_B \Rightarrow R^2_A = R^2_B\Delta^2_{AB}}$

Confirmation of the matrix equation-derived equation for R^2_A was obtained by analyzing a simulated mRNA expression/genotype data set, where levels of mRNA expression depended only on the alleles of a regulatory variant (SNP_B) and 3000 additional non-regulatory variants (each a SNP_A) were assigned varying degrees of LD with SNP_B. (Note: this figure is identical to the first graph in Supplementary File 5, Figure S1A.

Estimated R^2_A = coefficient of determination derived from linear regression analysis of the simulated mRNA expression versus genotype data for 1000 samples.

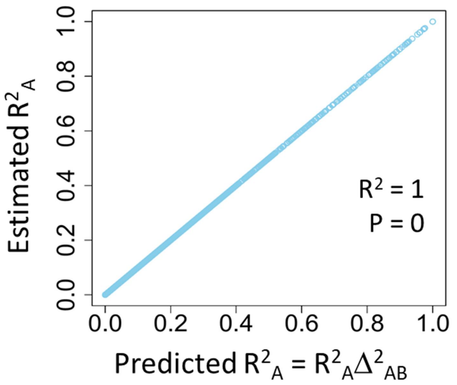

Δ^2_{AB} values were calculated based on haplotype frequencies in the simulated data set.

Two-rSNP systems (based on the constraint: $R^2_{ABC} = R^2_{BC}$)

$$R^2_{ABC} = [A, B, C]\, \mathbf{R}^{-1} \begin{bmatrix} A \\ B \\ C \end{bmatrix} = R^2_{BC}; \quad \mathbf{R} = \begin{bmatrix} 1 & a & c \\ a & 1 & b \\ c & b & 1 \end{bmatrix}$$

$$\mathbf{R}^{-1} = \text{adj}\mathbf{R}/\text{det}\mathbf{R} = \begin{bmatrix} 1-b^2 & bc-a & ab-c \\ bc-a & 1-c^2 & ac-b \\ bc-c & ac-b & 1-a^2 \end{bmatrix} 1/(1+2abc- a^2- b^2- c^2)$$

Solving for A, yields the equations:

$\Rightarrow$ $A = [B(a-bc) + C(c-ab)]/(1-b^2) = -\beta/2\alpha = [B(-b_{12}) + C(-b_{13})]/b_{11}$

$\Rightarrow$ $A^2 = [B(a-bc) + C(c-ab)]^2/(1-b^2)^2 = \beta^2/4\alpha^2 = [B(-b_{12}) + C(-b_{13})]^2/b_{11}^2$

where α and β are elements of the quadratic equation used in the derivation and $\mathbf{b}_{11}$, $\mathbf{b}_{12}$ and $\mathbf{b}_{13}$ are elements of the adjunct matrix, adj**R**. **Replacing, A,B,C, b_{11}, b_{12}, b_{13}:**

$\Rightarrow$ $\mathbf{R^2_A = [R_B(r_{AB} - r_{BC}r_{AC}) + R_C(r_{AC} - r_{AB}r_{BC})]^2/(1-r_{BC}^2)^2}$

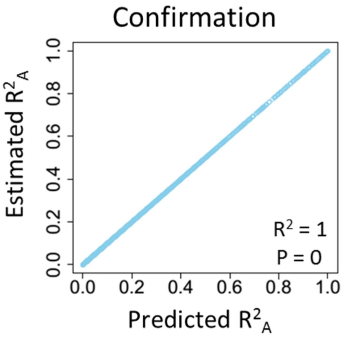

Three-rSNP systems (based on the constraint: $R^2_{ABCD} = R^2_{BCD}$)

$$R^2_{ABCD} = [A, B, C, D]\, \mathbf{R}^{-1} \begin{bmatrix} A \\ B \\ C \\ D \end{bmatrix} = R_{BCD}^2; \quad \mathbf{R} = \begin{bmatrix} 1 & a & c & f \\ a & 1 & b & d \\ c & b & 1 & e \\ f & d & e & 1 \end{bmatrix}; \quad \mathbf{R}^{-1} = \text{adj}\mathbf{R}/\text{det}\mathbf{R} =$$

$$\begin{bmatrix} 1+2bde-b^2-d^2-e^2 & ae^2+bc+df-a-cde-fbe & ab+cd^2+ef-ade-c-bdf & ad+ce+b^2f-abe-f-bcd \\ ae^2+bc+df-a-cde-fbe & 1+2cef-c^2-e^2-f^2 & de+ac+bf^2-b-cdf-aef & be+af+c^2d-d-ace-bcf \\ ab+cd^2+ef-ade-c-bdf & de+ac+bf^2-b-cdf-aef & 1+2adf-a^2-d^2-f^2 & bd+a^2e+cf-e-acd-abf \\ ad+ce+b^2f-abe-f-bcd & be+af+c^2d-d-ace-bcf & bd+a^2e+cf-e-acd-abf & 1+2abc-a^2-b^2-c^2 \end{bmatrix}$$

$$\times\, 1/[1 + a^2e^2 + b^2f^2 + c^2d^2 + 2abc + 2adf + 2bde + 2cef - a^2 - b^2 - c^2 - d^2 - e^2 - f^2 - 2abef - 2acde - 2bcdf]$$

Solving for A, yields the equations:

$\Rightarrow$ $A = [B(-ae^2 - bc - df + a + cde + fbe) + C(-ab - cd^2 - ef + ade + c + bdf) + D(-ad - ce - b^2f + abe + f + bcd)]$
$\times 1/(1 + 2bde - b^2 - d^2 - e^2) = -\beta/2\alpha = [B(-b_{12}) + C(-b_{13}) + D(-b_{14})]/b_{11}$

$\Rightarrow$ $A^2 = \beta^2/4\alpha^2 = [B(-b_{12}) + C(-b_{13}) + D(-b_{14})]^2/b_{11}^2$

where α and β are elements of the quadratic equation used in the derivation and $\mathbf{b}_{12}$, $\mathbf{b}_{13}$, and $\mathbf{b}_{14}$ are elements of the adjugate matrix, adj**R**.

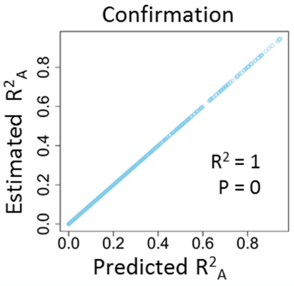

Note: the "Confirmation" data in the previous two boxes are identical to first plots in Supplementary File 5, Figures S1B and C.

By manually solving the matrix equations for R^2_A under the constraints $R^2_{AB} = R^2_B$, $R^2_{ABC} = R^2_{BC}$, or $R^2_{ABCD} = R^2_{BCD}$ (324 terms!), we noticed that in each case the quantity under the square root sign of the quadratic equation ($\beta^2 - 4\alpha\gamma$) equaled 0. Assuming this to be true for increasing numbers of index SNPs, matrix equation-based calculations of R^2_A can be easily carried out based on the formulas listed in the following table:

Where b_{11}, b_{12}, etc. are the indicated elements of the adjunct matrix, adjM, defined for each set of SNPs and $-\beta/2\alpha$ refer to elements of the quadratic equation used to solve for R_A:

$$R_A = \frac{-\beta \pm \sqrt{\beta^2 - 4\alpha\gamma}}{2\alpha}$$

	Index SNPs	Non-index SNP: $R_A = -\beta/2\alpha$; $R_A^2 = \beta^2/4\alpha^2$
1	SNP_B	$(R_B b_{12})^2/b_{11}^2$
2	SNP_B, SNP_C	$(R_B b_{12} + R_C b_{13})^2/b_{11}^2$
3	SNP_B, SNP_C, SNP_D	$(R_B b_{12} + R_C b_{13} + R_D b_{14})^2/b_{11}^2$
4	SNP_B, SNP_C, SNP_D, SNP_E	$(R_B b_{12} + R_C b_{13} + R_D b_{14} + R_E b_{15})^2/b_{11}^2$
5	SNP_B, SNP_C, SNP_D, SNP_E, SNP_F	$(R_B b_{12} + R_C b_{13} + R_D b_{14} + R_E b_{15} + R_E b_{16})^2/b_{11}^2$
N	SNP_B, SNP_C, SNP_D, SNP_E, SNP_F .. SNP_N	$(R_B b_{12} + R_C b_{13} + R_D b_{14} + R_E b_{15} + R_E b_{16} + \ldots + R_N b_{1N})^2/b_{11}^2$

(Note: This table is identical to Table 1 in the main text.)

As described in the boxes above, the following equations were obtained for R^2_A for systems containing one non-regulatory SNP_A and one, two or three regulatory variants:

$R^2_A = R^2_B \Delta^2_{AB}$ [1 term]

$R^2_A = [R_B(r_{AB} - r_{AC} r_{BC}) + R_C(r_{AC} - r_{AB} r_{BC})]^2/(1-r_{BC}^2)^2$ [16 terms]

$R^2_A = [R_B(r_{AB} r_{CD}^2 + r_{BC} r_{AC} + r_{BD} r_{AD} - r_{AB} - r_{AC} r_{BD} r_{CD} - r_{AD} r_{BC} r_{CD})$

$+ R_C(r_{AC} r_{BD}^2 + r_{AB} r_{BC} + r_{CD} r_{AD} - r_{AC} - r_{AB} r_{BD} r_{CD} - r_{AD} r_{BC} r_{BD})$

$+ R_D(r_{BC}^2 r_{AD} + r_{AB} r_{BD} + r_{AC} r_{CD} - r_{AD} - r_{AB} r_{BC} r_{CD} - r_{AC} r_{BC} r_{BD})]^2/(1 + 2r_{BC} r_{BD} r_{CD} - r_{BC}^2 - r_{BD}^2 - r_{CD}^2)^2$

[18 x 18 = 324 terms]

An interesting aspect of the above equations is that the multiple positive or negative terms in the equation can combine to (in some cases dramatically!) to increase or decrease the values of "predicted" R^2_A compared to the value one would naively expect based on the sums: $R_B^2 r_{AB}^2 + R_C^2 r_{AC}^2$ for two regulatory variants (in this case designated SNP_B and SNP_C) or

$R_B{}^2 r_{AB}{}^2 + R_C{}^2 r_{AC}{}^2 + R_D{}^2 r_{AD}{}^2$ for three regulatory variants (in this case designated SNP_B, SNP_C and SNP_D).

This apparently paradoxical result is illustrated in the following two figures. For simplicity, the contribution of the "extra" term in the equation $R^2{}_A$ for a non-regulatory variant, SNP_A, that is in linkage disequilibrium with two regulatory variants (SNP_B and SNP_C), which are in linkage equilibrium with each other (i.e., $r_{CB}{}^2 = r_{BC}{}^2 = 0$).

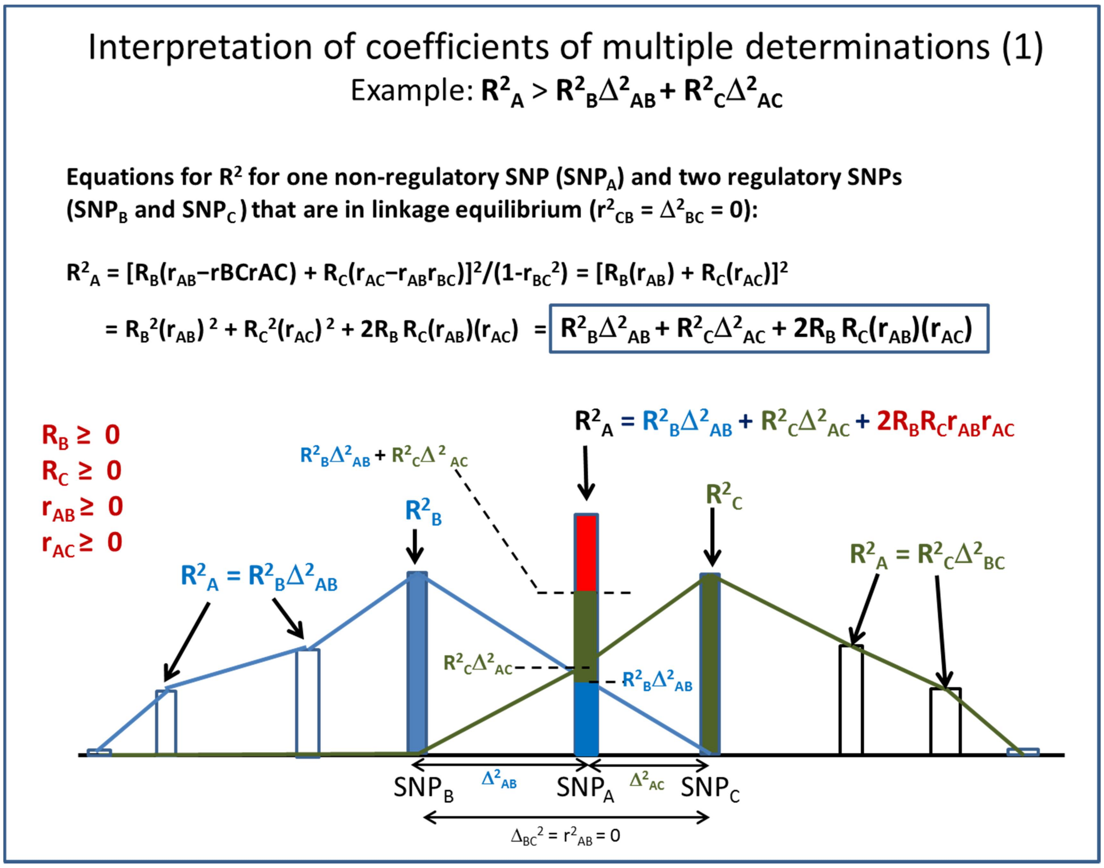

Interpretation of coefficients of multiple determinations (2)

Example: $\mathbf{R^2_A < R^2_B\Delta^2_{AB} + R^2_C\Delta^2_{AC}}$

Equations for R^2 for one non-regulatory SNP (SNP_A) and two regulatory SNPs (SNP_B and SNP_C) that are in linkage equilibrium ($r^2_{BC} = \Delta^2_{BC} = 0$):

$$R_A^2 = [R_B(r_{AB} - r_{BC}r_{AC}) + R_C(r_{AC} - r_{AB}r_{BC})]^2/(1-r^2_{BC}) = [R_B(r_{AB}) + R_C(r_{AC})]^2$$

$$= R^2_B(r_{AB})^2 + R^2_C(r_{AC})^2 + 2R_B R_C(r_{AB})(r_{AC}) = \boxed{R^2_B\Delta^2_{AB} + R^2_C\Delta^2_{AC} + 2R_B R_C(r_{AB})(r_{AC})}$$

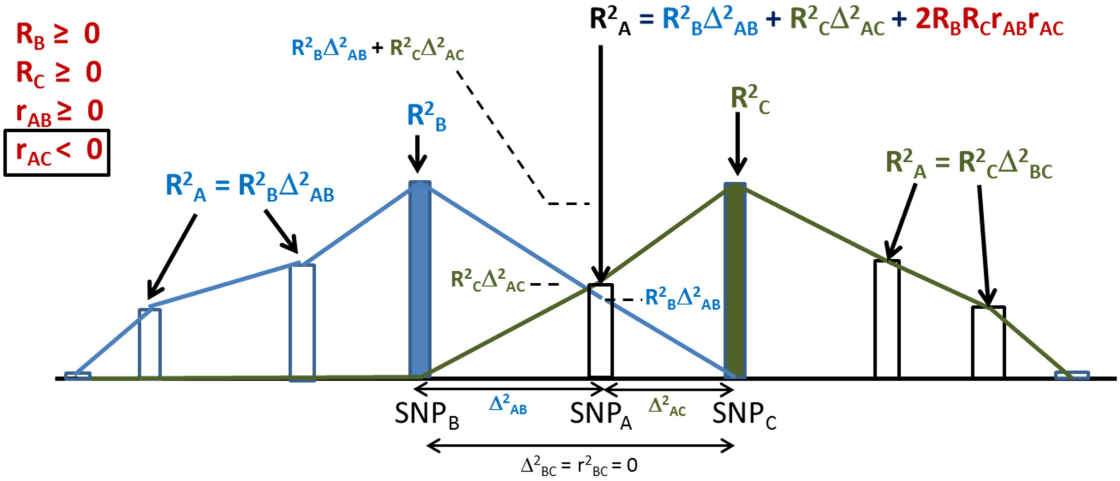

The resolution of this apparent paradox comes with the understanding that the bars in the diagram representing R^2 are mathematically representing a squared, not a linear, quantity. The positive and negative terms in the equations for R^2_A thus sum to an "area" rather than a length. The individual terms that contribute to R^2_A individually do not have an easily physical interpretation, but rather simply represent a tiling of the R^2_A "area" with positive and negative fragments of (abstract) area.

To determine the size and frequency at which positive and negative (rAB)(rAC) are encountered in representative sets of 8 three-allele haplotypes, we carried out the simulation described in the following box.

Calculated R^2_A values with terms containing $r_{AB}r_{AC} < 0$ are common:

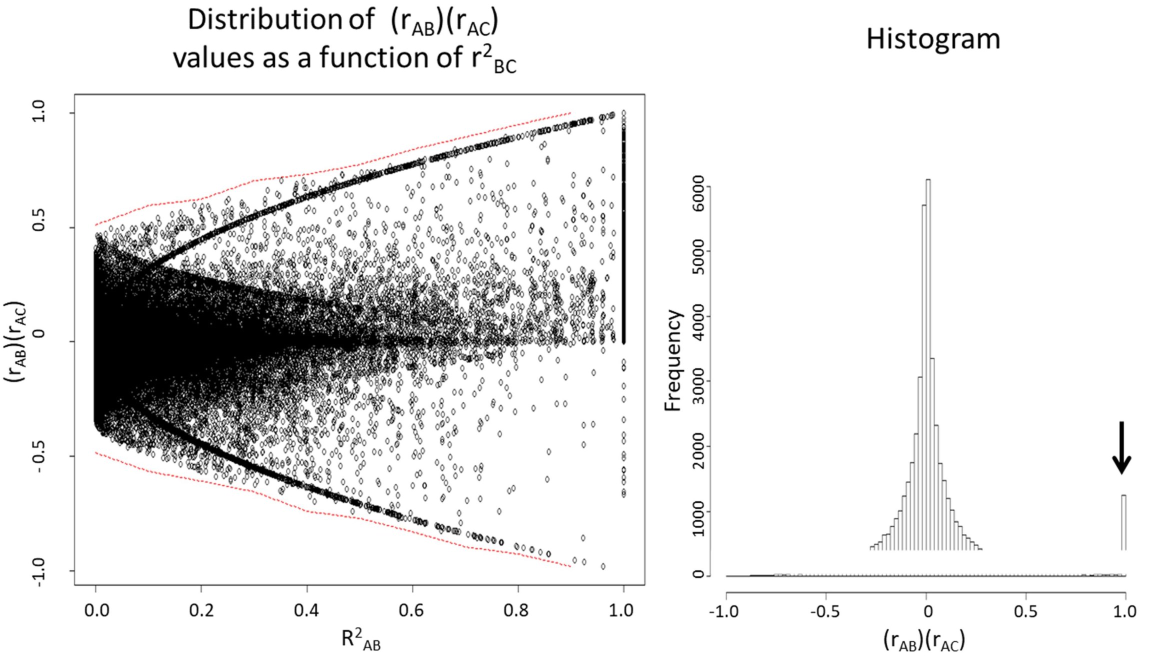

(50,000 random assignment of haplotype frequencies for SNP_A, SNP_B and SNP_C alleles, where the sum of the frequencies = 1 (1000 individuals/sample)

Extending this analysis to a four variant system comprising one non-regulatory variant (SNP_A) and three regulatory variants (SNP_B, SNP_C and SNP_D), we noticed that among the 324 terms the equation defining R^2_A, only terms containing $a = r_{AB}$, $c = r_{AC}$ and/or $f = r_{AD}$ are free to vary once the three regulatory variants have been fixed: i.e., the values of $b = r_{BC}$, $d = r_{BD}$ and $e = r_{CD}$ are constant over multiple simulations involving different choices of SNP_A. Likewise, values for R_A, R_B, R_C, and R_D can be held constant. Under these assumptions, the 324 terms in the above equation can be reduced to six terms: a^2k_1, ack_2, afk_3, c^2 k_4 cfk_5 or f^2k_6, Where k_1 – k_6 represent constant quantities with different values (calculations not shown).

The following graph plots the values of these six terms for 100 SNPs + 3 regulatory variants

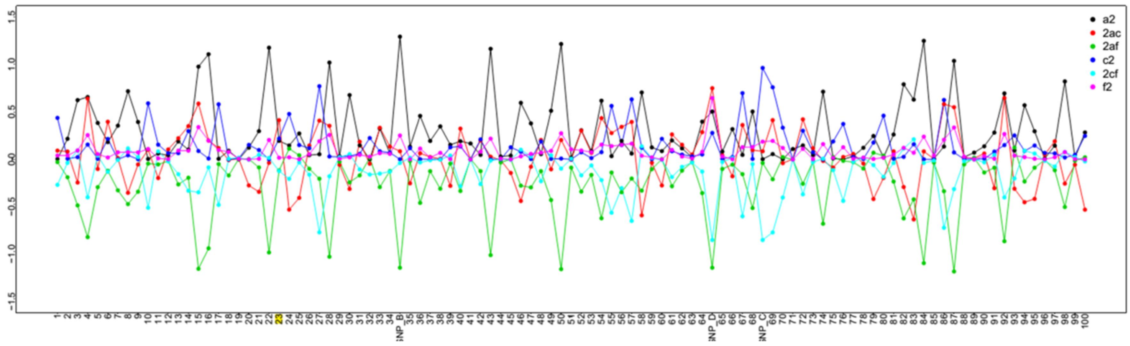

The following graph and table show how the six terms sum to values that match the "estimated" R^2_A values for the dataset for selected SNPs with R^2_A values that differ significantly from the sum of contributions of the regulatory variants expected from LD alone (column 10 in the table). The red arrows identify selected SNPs for which the "estimated" R^2_A (blue bars in upper and lower plots) is dramatically larger than the expected sum of the contribution of regulatory variants expected from LD alone. The black arrows identify selected SNPs for which the "estimated" R^2_A is dramatically smaller than expected from this sum. For all of the SNPs in this simulated data set, however, these is excellent agreement between "estimated" R^2_A values and "predicted" R^2_A values (red line in upper plot) calculated as described above.

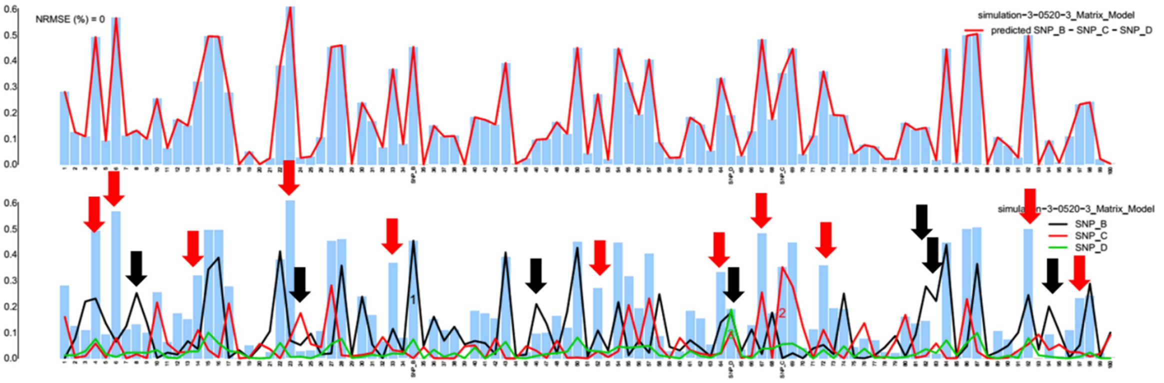

SNP #	"Measured" R2A	"Calculated" R2A	a2	2ac	2af	c2	2cf	f2	Sum of thr
4	0.4915	0.491466211	0.65815	0.6421	-0.82094	0.15661	-0.40046	0.256	0.36222
6	0.565	0.564989268	0.18261	0.39738	-0.1202	0.21619	-0.13078	0.01978	0.14853
14	0.319	0.318990043	0.10252	0.35051	-0.19387	0.29961	-0.33143	0.09166	0.17184
23	0.6071	0.607142209	0.19546	0.41413	-0.11608	0.21936	-0.12297	0.01723	0.15343
33	0.3676	0.367626753	0.32501	0.33394	-0.29264	0.08578	-0.15034	0.06587	0.1643
52	0.2719	0.271874875	0.30716	0.304	-0.34037	0.07522	-0.16843	0.09429	0.16248
64	0.3328	0.332809643	0.39811	0.28763	-0.35586	0.05195	-0.12855	0.07952	0.18154
67	0.4817	0.481714938	0.04702	0.36274	-0.15577	0.69954	-0.60082	0.12901	0.30908
72	0.3591	0.359115301	0.14751	0.42261	-0.25731	0.30268	-0.36858	0.11221	0.1947
92	0.4967	0.496738118	0.69647	0.64727	-0.86384	0.15039	-0.40141	0.26786	0.37682
97	0.2318	0.231824733	0.14465	0.1913	-0.11436	0.06325	-0.07562	0.0226	0.08031
8	0.1311	0.131094264	0.71959	-0.35285	-0.47205	0.04325	0.11573	0.07742	0.29035
24	0.0259	0.025945519	0.14666	-0.53028	0.11358	0.47932	-0.20533	0.02199	0.2325
46	0.0958	0.0958	0.59736	-0.43893	-0.27734	0.08063	0.10189	0.03219	0.24798
58	0.0859	0.085944719	0.70671	-0.59066	-0.32986	0.12342	0.13785	0.03849	0.30371
82	0.142	0.14204715	0.793	-0.29179	-0.62296	0.02684	0.11461	0.12235	0.32317
83	0.0169	0.016891041	0.63091	-0.6336	-0.42175	0.15908	0.21178	0.07048	0.29949
94	0.0918	0.091813779	0.57171	-0.45194	-0.23326	0.08932	0.0922	0.02379	0.23972

Note: in this table "Measured" R2A = "Estimated" R^2_A and "Calculated" R2A = "Predicted" R^2_A as defined in the main text, Box 1.

Supplementary File 4.
Simulation of mRNA expression/genotype data sets

A. Construction of genotype data sets based on the random assignment of haplotype frequencies.

Genotype datasets based on haplotype frequencies calculated from preassigned minor allele frequencies and LD coefficient values can be easily constructed for two- and three-allele haplotype systems using equations described in Robinson, Asmussen and Thomson [1] and listed in section D, below. This approach becomes exceeding cumbersome, however, for haplotypes containing four or more alleles. For example, 16 equations, each containing 12 terms comprising major and minor allele frequencies, second-order, third-order and fourth-order LD coefficients, are required to calculate the frequencies of the 16 possible four-allele haplotypes. In addition, inequalities containing: i) 8 lower and 8 upper bounds, each comprising 11 terms, are required to define the constraints on the fourth-order LD coefficients, ii) 16 lower and 16 upper bounds, each comprising 4 or 13 terms, are required to define the constraints on the third-order LD coefficients, and iii) 6 lower and 6 upper bounds, each comprising 4 terms, are required to define the constraints on the second-order LD coefficients [1] and section D, below).

To avoid these complexities, we used a simpler approach for constructing genotype data sets based on the random assignment of haplotype frequencies, subject to the constraint that the sum of the frequencies for each set of haplotypes equal 1. Sets of haplotype frequencies that sum to 1 were produced by: i) using the sample() command in R to select (with replacement) a set of j numbers from a large set of contiguous integers (for example 0-100), where j = the number of haplotypes in a complete set of haplotypes: j = 4 for two-SNP systems, j = 8 for three-SNP systems, j = 16 for four SNP systems, etc., and ii) dividing each of the these numbers by the sum of all j numbers selected in the set.

Diplotype frequencies were generated by pairwise multiplication of all possible combinations of haplotype frequencies: or example, 16 x 16 = 256 diplotype frequencies for all possible pairwise combinations of four-allele haplotypes. For each individual in the sample, genotypes are extracted from the diplotypes, without considerations of the physical linkage (LD) of alleles. For a typical simulation, 1000 – 3000 independent genotype data sets were constructed from 1000 - 3000 sets of randomly assigned haplotype frequencies.

Because the products of sample size x diplotype frequencies usually do not yield integer values, small adjustments are required to assign integer values for the number of individuals for each of the possible diplotypes in manner that does not change the original sample size, n. This can be accomplished by the following procedure:

1) The values of n x diplotype frequencies (column 5 in the table in section D.1, below) are ordered according to the size of their <u>decimal portions</u>: (i.e., xxx.987, xxx.750, xxx.334, etc.).

2) The decimal portion of these numbers is summed, yielding a number close in value to n minus the sum of the integer portion of the numbers in column 5 = k. For The number k ranges in value from 0 to 16 for 4 x 4 = 16 diplotypes generated from two-allele haplotypes and represents the number of individuals that must be restored to the data set to maintain the original dataset size.

3) Restoration of the original sample size is accomplished by adding 1 to the integer portion of the values in ordered list described in step I above, beginning with the number with the largest decimal portion and proceeding one-by-one down the list until k individuals have been added.

Allele frequencies and the values of LD coefficients calculated using the resulting dataset differ only slightly from their original values and do not significantly change the results of subsequent analyses. The above procedure is useful because it facilitates the construction of data sets containing many SNPs, while maintaining a constant sample size

It can be shown that sets of haplotype frequencies that sum to 1 are self-consistent by carrying out all possible: i) pairwise addition of the polynomial equations defining the frequency of each haplotype [1]. For example, equations for four-allele haplotype frequencies can be summed to produce equations for three-allele haplotype frequencies, ii) pairwise addition of these equations yield equations for the frequencies of two-allele haplotypes and iii) pairwise addition of these equations yield allele-frequencies (calculations not shown).

To determine whether this procedure yields representative sets of minor allele frequencies and LD coefficients (which are used as the input variables in Method 1, above), we generated 3000 independent data sets for systems with one non-regulatory variant and one, two or three regulatory variants and

examined the ranges and distributions of minor allele frequencies and LD coefficients. These results confirm that the minor allele frequencies and LD coefficients can take the full spectrum of permitted values and confirm that representative genotype datasets can be generated starting from sets of haplotypes with randomly assign population frequencies. (**See Figures S3.1 – S3.5, below.**)

To construct sets of population genotypes for variable choices of non-regulatory SNP_A and fixed regulatory variants SNP_B, SNP_C and SNP_D, we first constructed sets of two- or three-allele haplotypes for the SNP combinations SNP_B and SNP_C or SNP_B, SNP_C and SNP_D, respectively, ether by random assignment as described above, or based on the equations for haplotype frequencies described in section D, below, in cases where models containing specific values for R_B^2, R_C^2, R_D^2 and/or pairwise Δ^2 LD coefficients were required. In the latter case, careful attention was paid to constraints on the values of second- and third-order LD coefficients for these SNPs. Frequencies for sets of augmented haplotypes containing either the major (A) or minor (a) allele of SNP were then calculated by multiplying the frequency of the A or a allele times the frequencies of the original set of haplotypes. The frequencies of the resulting three- or four-allele haplotypes again sum to 1, and therefore can be used for the construction of sets of population genotypes as described above.

B. Construction of mRNA expression data sets using Fisher's "genotypic values" [2] **to assign mRNA expression levels**

In general, phenotypic variance (V_P) can be partitioned into additive genetic (V_G), environmental (V_E) and G x E (i.e., interactive) (V_{GE}) components:

$$V_P = V_G + V_E + V_{GE}$$

Genetic variance can be further partitioned into additive (V_A), dominant (V_D) and interactive (i.e., epistatic) (V_I) components:

$$V_G = V_A + V_D + V_I$$

In our model, we assume that there are no environmental, interactive or epistatic contributions to the variance of mRNA expression. The phenotypic (i.e., mRNA expression) variance is therefore expressed as the sum of the additive and dominant genetic contributions:

$V_P = V_A + V_D$

Following Fisher's method, we assign "genotypic values" for mRNA expression for the homozygote genotypes, AA and aa, as –a and +a, respectively, and a value of d for heterozygote genotype, Aa, of a biallelic regulatory variant (rVar). [A little confusingly, in Fisher's nomenclature, "genotype values" refer to the values of mRNA expression (i.e., "phenotype values") contributed by specific genotypes.] It can be shown that the additive and dominant genetic variance can be expressed in terms of allele frequencies and genotypic values by the following equations [ref: Falconer & Mackay, page 126]:

$V_A = 2pq[a + d(q-p)]^2$

$V_D = (2pqd)^2$

Where p = population frequency of the (major) A-allele = P_A and q = population frequency of the (minor) a-allele = P_a.
The total phenotypic variance can therefore be written:

$V_P = 2pq[a + d(q-p)]^2 + (2pqd)^2$

In the absence of dominant genetic effects, d is equal to zero, yielding:

$V_P = 2pqa^2 = V_A$.

The genotypic value, a, can therefore be expressed in terms of the total phenotypic variance and allele frequencies as:

$a = sqrt(V_A/2pq)$.

The population "mean genotypic value (M_G)" is the sum of the contributions to mRNA expression of all heterozygotes and homozygotes in the population:

$M_G = p^2a + 2pqd + q^2(-a) = a(p-q) + 2pqd$ (Falconer & Mackay, page 110)

Where p^2 and q^2 are the frequencies of homozygotes for the major and minor alleles, respectively, and 2pq the frequency of heterozygotes of populations in Hardy-Weinberg equilibrium.

The genotypic value for each genotype can be defined with respect to the population mean genotypic value by the following equations:

$g_{aa} = a – M_G = a – (a(p-q) + 2pqd)$
$g_{Aa} = d – M_G = d – (a(p-q) + 2pqd)$
$g_{AA} = -a – M_G = -a – (a(p-q) + 2pqd)$

In the absence of dominance, these equations become:

$g_{aa} = a – M_G = a – a(p-q) = a[1 - (p-q)] = sqrt(V_A/2pq) [1 - (p-q)]$
$g_{Aa} = d – M_G = – a(p-q) = -a(p-q) = - sqrt(V_A/2pq)(p-q)]$
$g_{AA} = -a – M_G = -a – a(p-q) = -a[1 - (p-q)] = - sqrt(V_A/2pq)[1 - (p-q)]$

In the case of multiple regulatory variants, the genotypic values for each variant can be calculated independently as a function of its contribution to the variance of mRNA expression variant and its allele frequencies:

$g_{aa(rvar1)} = sqrt(V_{rVar1}/2p_{rVar1}q_{rvar1}) [1 - (p_{rVar1}-q_{rVar1})]$
$g_{Aa(rvar1)} = - sqrt(V_{rVar1}/2p_{rVar1}q_{rvar1})(p_{rVar}-q_{rVar1})]$
$g_{AA(rSNP1)} = - sqrt(V_{rVar1}/2p_{rVar1}q_{rvar1})[1 - (p_{rVar1}-q_{rVar1})]$

and likewise, for rVar2, rVar3, etc.

Using these equations, the range of genotypic values for genotypes of each rVarj can assigned by varying the parameters, V_{rVarj} and q_{rVarj}.

For each individual in the population, the total genotypic value for mRNA expression is calculated as the sum of the contributions of the genotypic values for each SNP. For example, two regulatory variants:

g_{total} = [$g_{AA(rVar1)}$ or $g_{Aa(rVar1)}$ or $g_{aa(rVar1)}$] + [$g_{AA(rVar2)}$ or $g_{Aa(rVar2)}$ or $g_{aa(rVar2)}$] = $g_{rVar1} + g_{rVar2}$

Likewise, for systems with three or more regulatory variants.

In the case of multiple rVars, the total additive variance (V_A) can be partitioned into the contributions of each variant and their covariance. Using this notation for a system containing two rVar:

$$V_A = V_{gtotal} = V_{grVar1} + V_{grVar2} + 2cov(g_{rVar1}, g_{rVar2}),$$

where V_A = total phenotypic variance, V_{rVar1} = variance of genotypic values of rVar1; V_{rVar2} = variance of genotypic values of rVar2; cov(grVar1, grVar2) = covariance of genotypic values of rVar1 and rVar2. For increasing numbers of rVars, V_A can be similarly expressed as a linear combination of multiple variance and covariance terms. The validity of this equation was tested using a simulated mRNA expression/genotype dataset. See **Figure S3.6** in section C, below.

Error terms are introduced into the simulation by adding to g_{total} for each individual values randomly selected from normal distributions of error terms with mean = 0 and standard deviation = f = 1/20, 1/10 or 1/5 of the genetic value of the total additive variance of mRNA expression (VA). The results of adding error-terms into simulated mRNA expression/genotype datasets are shown in **Figure S3.7**, below.

C. Example simulations

Figures S3.1 – **Figure S3.5** show plots of the distributions of three- and two-allele haplotype frequencies and values of LD coefficients obtained from 3000 sets randomly assignments frequencies for three-allele haplotypes. Protocol: i) Eight numbers were randomly selected, with replacement, from a set of integers ranging from 0 to 100 using the sample() function in R. ii) Each of the 8 numbers was then divided by the

sum of the 8 numbers, thereby yielding a set of 8 decimals that sum to 1. iii) Step i) and step ii) were repeated 3000 times and the results stored in a spreadsheet.

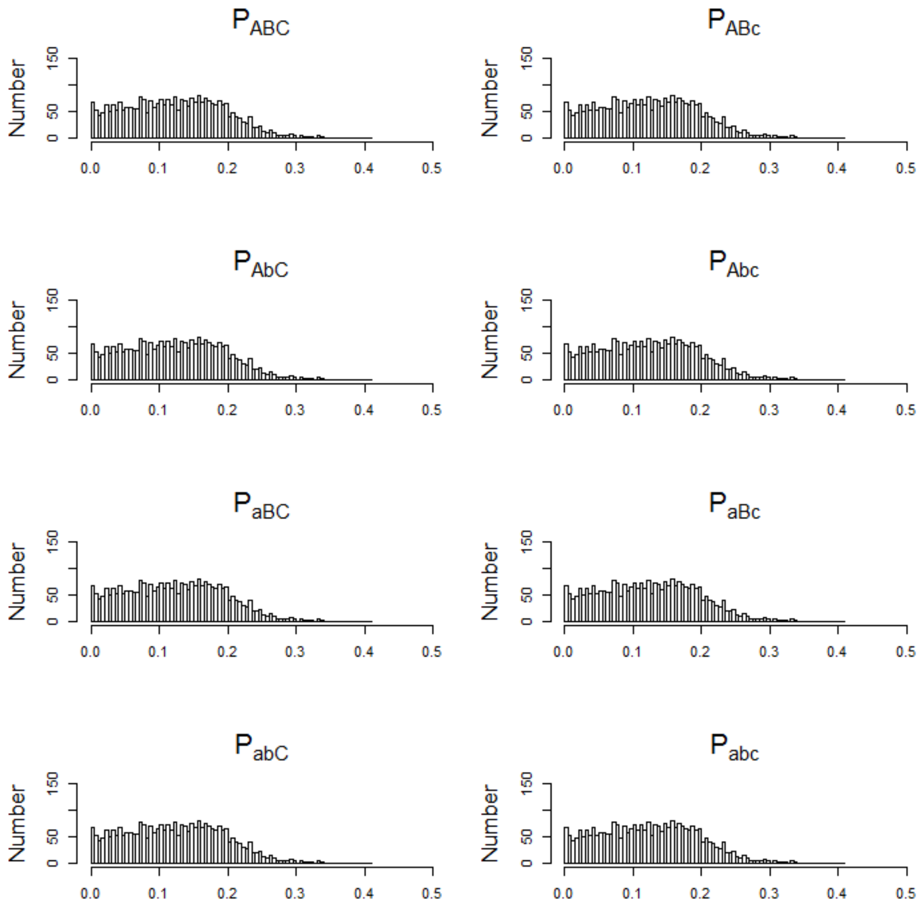

Figure S3.1 Distributions of three-allele haplotype frequencies. X-axis: haplotype frequencies (0 – 0.5) sorted into 100 "bins." Y-axis = number of simulations that produced frequencies falling within the corresponding bin for each of 8 possible haplotypes. In this simulation, the A, a, B, b, C, and c serve only as labels for the alleles of SNP_A, SNP_B and SNP_C, with each letter free to represent a major or minor allele depending on the simulation.

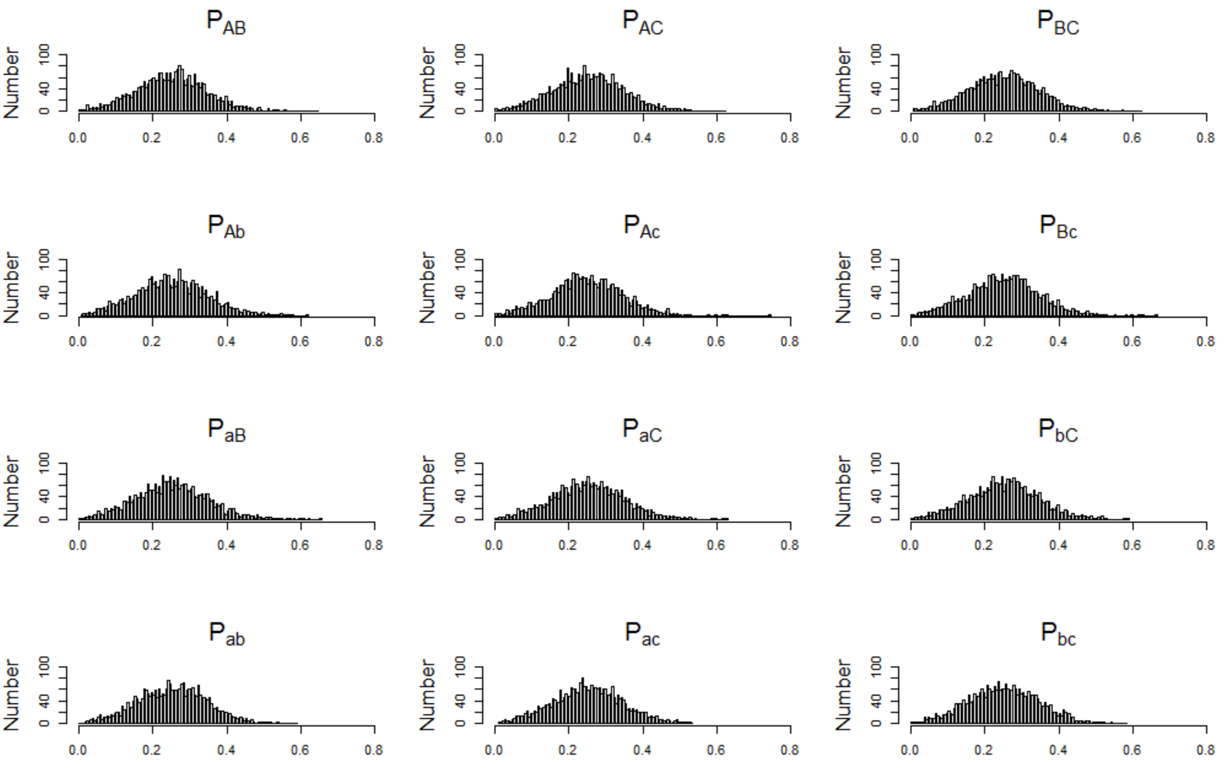

Figure S3.2 Distributions of two-allele haplotypes frequencies, plotted as described in the previous figure.

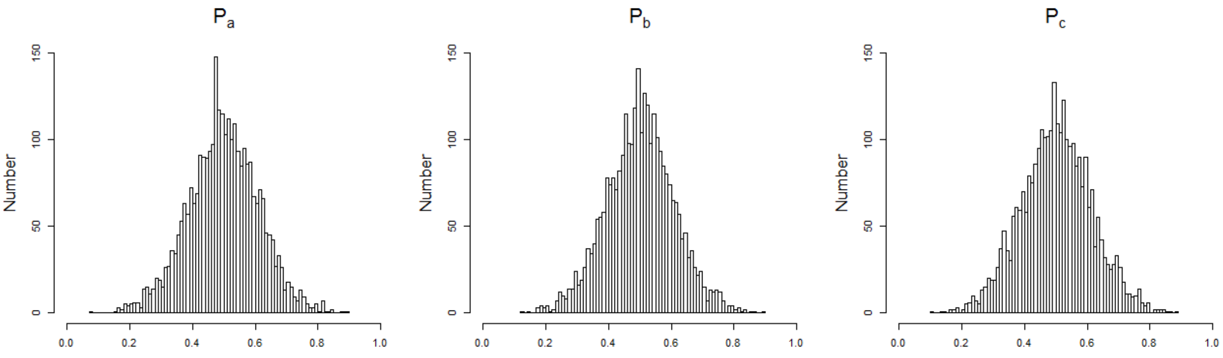

Figure S3.3 Distributions of allele frequencies for SNP_A, SNP_B and SNP_C, plotted as described above.

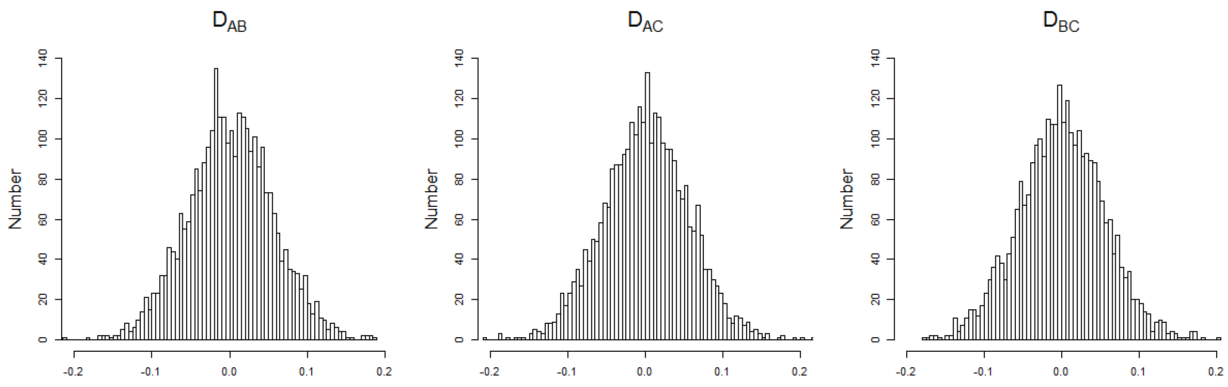

Figure S3.4 Distribution of values of second-order LD coefficients, plotted as described above.

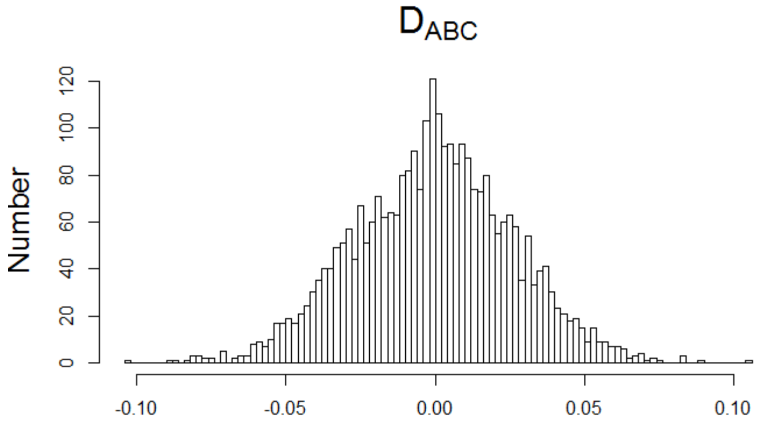

Figure S3.5 Distribution of values for the third-order LD coefficients, plotted as described above.

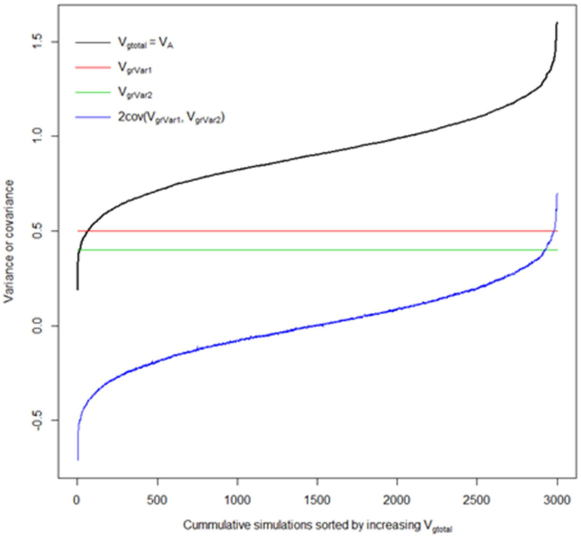

Figure S3.6. Demonstration that $V_A = V_{gtotal} = V_{grVar1} + V_{grVar2} + 2cov(g_{rVar1}, g_{rVar2})$ for a system with two regulatory variants (rVar1 and Var2). Graph shows values for variance or covariance (y-axis) plotted against values of V_{gtotal} sorted in order of increasing size (x-axis) obtained from the analysis of 3000 simulated datasets.

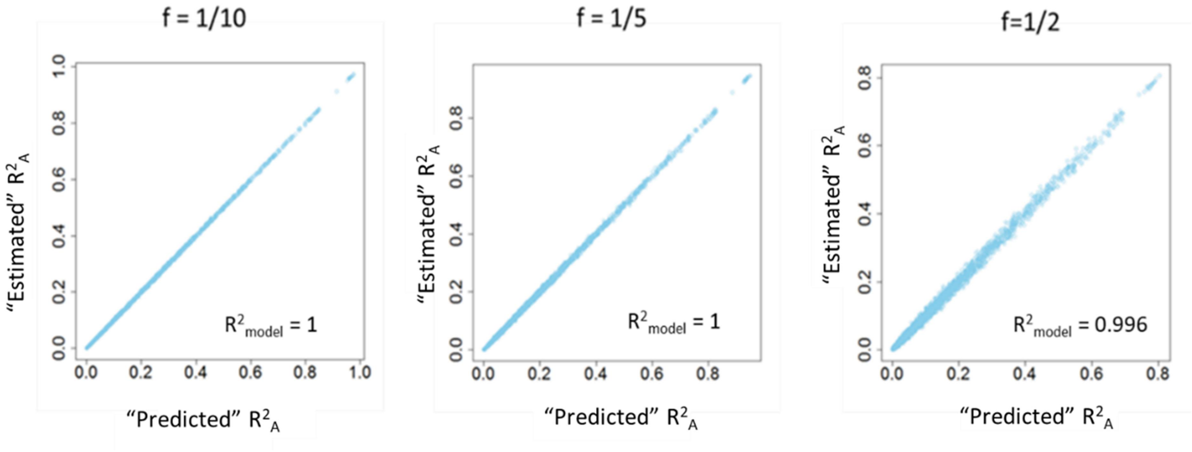

Figure S3.7. Graphs show plots of "estimated" R^2_A values obtained by linear regression analysis of simulated datasets comprising 3000 SNPs [two rVars (SNP_B and SNP_C) + 3000 non-regulatory SNPs, each designated as a SNP_A vs. "predicted" R^2_A values obtained from the matrix equation under the constraint $R^2_{ABC} = R^2_{BC}$. Each point in the graph represents the results of one of 3,000 simulations. Increasing levels of experimental error were simulated by adding values randomly selected from a normal distribution

with mean = zero and standard deviation equal to fV_A to g_{total} for each individual. R^2_{Model} = the coefficient of determination obtained from linear regression analysis of the plotted data.

D. Polynomial equations for haplotype frequencies and constraints on LD coefficients in two-, three- and four-SNP systems

1. Frequencies for two-allele haplotypes:

$P_{AB} = P_A P_B + D_{AB}$
$P_{Ab} = P_A P_b - D_{AB}$
$P_{aB} = P_a P_B - D_{AB}$
$P_{ab} = P_a P_b + D_{AB},$

where the second-order LD coefficient D_{AB} is subject to the constraint:

$\max(-P_A P_B, -P_a P_b) \leq D_{AB} \leq \min(P_A P_b, P_a P_B)$

Input values: minor-allele frequencies of SNP_A and SNP_B and D_{AB} linkage disequilibrium coefficient. [Note: D_{AB} coefficients are often calculated from r^2 LD coefficients: $r^2 = D_{AB}^2/(P_A P_a P_B P_b)$; $D_{AB} = \mathrm{sqrt}(P_A P_a P_B P_b)r$.]

These haplotype frequencies are then used to calculate the frequencies of all possible diplotypes:

	P_{AB}	P_{Ab}	P_{aB}	P_{ab}
P_{AB}	P_{AB}/P_{AB}	P_{AB}/P_{Ab}	P_{AB}/P_{aB}	P_{AB}/P_{ab}
P_{Ab}	P_{Ab}/P_{AB}	P_{Ab}/P_{Ab}	P_{Ab}/P_{aB}	P_{Ab}/P_{ab}
P_{aB}	P_{aB}/P_{AB}	P_{aB}/P_{Ab}	P_{aB}/P_{aB}	P_{aB}/P_{ab}
P_{ab}	P_{ab}/P_{AB}	P_{ab}/P_{Ab}	P_{ab}/P_{aB}	P_{ab}/P_{ab}

Diplotype frequencies are then used to calculate the number of individuals within the sample that harbor specific combinations of genotypes

Diplotype	SNP_A genotype	SNP_B genotype	Frequency in sample	Number of individuals with the indicated combination of genotypes within sample size = n before rounding to nearest integer	Integer portion	Decimal portion
AB/AB	AA	BB	$P_{AB}P_{AB}$	$nP_{AB}P_{AB}$		
Ab/AB	AA	Bb	$P_{AB}P_{Ab}$	$nP_{AB}P_{Ab}$		
AB/Ab	AA	Bb	$P_{AB}P_{Ab}$	$nP_{AB}P_{Ab}$		
aB/AB	Aa	BB	$P_{AB}P_{aB}$	$nP_{AB}P_{aB}$		
AB/aB	Aa	BB	$P_{AB}P_{aB}$	$nP_{AB}P_{aB}$		
Ab/Ab	AA	bb	$P_{Ab}P_{Ab}$	$nP_{Ab}P_{Ab}$		

ab/AB	Aa	Bb	$P_{ab}P_{AB}$	$nP_{ab}P_{AB}$		
AB/ab	Aa	Bb	$P_{ab}P_{AB}$	$nP_{ab}P_{AB}$		
aB/Ab	Aa	Bb	$P_{aB}P_{Ab}$	$nP_{aB}P_{Ab}$		
Ab/aB	Aa	Bb	$P_{aB}P_{Ab}$	$nP_{aB}P_{Ab}$		
aB/aB	aa	BB	$P_{aB}P_{aB}$	$nP_{aB}P_{aB}$		
Ab/ab	Aa	bb	$P_{ab}P_{Ab}$	$nP_{ab}P_{Ab}$		
ab/Ab	Aa	bb	$P_{ab}P_{Ab}$	$nP_{ab}P_{Ab}$		
ab/aB	aa	Bb	$P_{ab}P_{aB}$	$nP_{ab}P_{aB}$		
aB/ab	aa	Bb	$P_{ab}P_{aB}$	$nP_{ab}P_{aB}$		
ab/ab	aa	bb	$P_{ab}\,P_{ab}$	$nP_{ab}\,P_{ab}$		
		Sum =	1.0	n	N - j	j

2. Frequencies of three-allele haplotypes:

01) $P_{ABC} = P_AP_BP_C + P_AD_{BC} + P_BD_{AC} + P_CD_{AB} + D_{ABC}$
02) $P_{aBC} = P_aP_BP_C + P_aD_{BC} - P_BD_{AC} - P_CD_{AB} - D_{ABC}$
03) $P_{AbC} = P_AP_bP_C - P_AD_{BC} + P_bD_{AC} - P_CD_{AB} - D_{ABC}$
04) $P_{abC} = P_aP_bP_C - P_aD_{BC} - P_bD_{AC} + P_CD_{AB} + D_{ABC}$
05) $P_{ABc} = P_AP_BP_c - P_AD_{BC} - P_BD_{AC} + P_cD_{AB} - D_{ABC}$
06) $P_{aBc} = P_aP_BP_c - P_aD_{BC} + P_BD_{AC} - P_cD_{AB} + D_{ABC}$
07) $P_{Abc} = P_AP_bP_c + P_AD_{BC} - P_bD_{AC} - P_cD_{AB} + D_{ABC}$
08) $P_{abc} = P_aP_bP_c + P_aD_{BC} + P_bD_{AC} + P_cD_{AB} - D_{ABC}$

i) Constraint on the third-order LD coefficient:

$\max(-P'_{ABC}, -P'_{abC}, -P'_{aBc}, -P'_{Abc}) \leq D_{ABC} \leq \min(P'_{aBC}, P'_{AbC}, P'_{ABc}, P'_{abc})$, where

$P'_{ABC} = P_AP_BP_C + P_AD_{BC} + P_BD_{AC} + P_CD_{AB}$
$P'_{aBC} = P_aP_BP_C + P_aD_{BC} - P_BD_{AC} - P_CD_{AB}$
$P'_{AbC} = P_AP_bP_C - P_AD_{BC} + P_bD_{AC} - P_CD_{AB}$
$P'_{abC} = P_aP_bP_C - P_aD_{BC} - P_bD_{AC} + P_CD_{AB}$
$P'_{ABc} = P_AP_BP_c - P_AD_{BC} - P_BD_{AC} + P_cD_{AB}$
$P'_{aBc} = P_aP_BP_c - P_aD_{BC} + P_BD_{AC} - P_cD_{AB}$
$P'_{Abc} = P_AP_bP_c + P_AD_{BC} - P_bD_{AC} - P_cD_{AB}$
$P'_{abc} = P_aP_bP_c + P_aD_{BC} + P_bD_{AC} + P_cD_{AB}$

ii) Constraints on second-order LD coefficients:

$\max(-P_AP_B, -P_aP_b) \leq D_{AB} \leq \min(P_AP_b, P_aP_B)$
$\max(-P_AP_C, -P_aP_c) \leq D_{AC} \leq \min(P_AP_c, P_aP_C)$
$\max(-P_BP_C, -P_bP_c) \leq D_{BC} \leq \min(P_BP_c, P_bP_C)$

and

$-P_AP_BP_C - P_aP_bP_c \leq D_{AB} + D_{AC} + D_{BC}$
$-P_AP_BP_c - P_aP_bP_C \leq D_{AB} - D_{AC} - D_{BC}$
$-P_AP_bP_C - P_aP_BP_c \leq -D_{AB} + D_{AC} - D_{BC}$
$-P_AP_bP_c - P_aP_BP_C \leq -D_{AB} - D_{AC} + D_{BC}$

3. Frequencies of four-allele haplotypes:

01) $P(ABCD) = P_AP_BP_CP_D + P_AD_{BCD} + P_BD_{ACD} + P_CD_{ABD} + P_DD_{ABC} + P_AP_BD_{CD} + P_AP_CD_{BD} + P_AP_DD_{BC} + P_BP_CD_{AD} + P_BP_DD_{AC} + P_CP_DD_{AB} + D_{ABCD}$
02) $P(ABCd) = P_AP_BP_CP_d - P_AD_{BCD} - P_BD_{ACD} - P_CD_{ABD} + P_dD_{ABC} - P_AP_BD_{CD} - P_AP_CD_{BD} + P_AP_dD_{BC} - P_BP_CD_{AD} + P_BP_dD_{AC} + P_CP_dD_{AB} - D_{ABCD}$

03) $P(aBCD) = P_aP_BP_CP_D + P_aD_{BCD} - P_BD_{ACD} - P_CD_{ABD} - P_DD_{AB\,C} + P_aP_BD_{CD} + P_aP_CD_{BD} + P_aP_DD_{BC} - P_BP_CD_{AD} - P_BP_DD_{AC} - P_CP_DD_{AB} - D_{ABCD}$
04) $P(aBCd) = P_aP_BP_CP_d - P_aD_{BCD} + P_BD_{ACD} + P_CD_{ABD} - P_dD_{ABC} - P_aP_BD_{CD} - P_aP_CD_{BD} + P_aP_dD_{BC} + P_BP_CD_{AD} - P_BP_dD_{AC} - P_CP_dD_{AB} + D_{ABCD}$
05) $P(AbCD) = P_AP_bP_CP_D - P_AD_{BCD} + P_bD_{ACD} - P_CD_{ABD} - P_DD_{ABC} + P_AP_bD_{CD} - P_AP_CD_{BD} - P_AP_DD_{BC} + P_bP_CD_{AD} + P_bP_DD_{AC} - P_CP_DD_{AB} - D_{ABCD}$
06) $P(AbCd) = P_AP_bP_CP_d + P_AD_{BCD} - P_bD_{ACD} + P_CD_{ABD} - P_dD_{ABC} - P_AP_bD_{CD} + P_AP_CD_{BD} - P_AP_dD_{BC} - P_bP_CD_{AD} + P_bP_dD_{AC} - P_CP_dD_{AB} + D_{ABCD}$
07) $P(abCD) = P_aP_bP_CP_D - P_aD_{BCD} - P_bD_{ACD} + P_CD_{ABD} + P_DD_{ABC} + P_aP_bD_{CD} - P_aP_CD_{BD} - P_aP_DD_{BC} - P_bP_CD_{AD} - P_bP_DD_{AC} + P_CP_DD_{AB} + D_{ABCD}$
08) $P(abCd) = P_aP_bP_CP_d + P_aD_{BCD} + P_bD_{ACD} - P_CD_{ABD} + P_dD_{ABC} - P_aP_bD_{CD} + P_aP_CD_{BD} - P_aP_dD_{BC} + P_bP_CD_{AD} - P_bP_dD_{AC} + P_CP_dD_{AB} - D_{ABCD}$
09) $P(ABcD) = P_AP_BP_cP_D - P_AD_{BCD} - P_BD_{ACD} + P_cD_{ABD} - P_DD_{ABC} - P_AP_BD_{CD} + P_AP_cD_{BD} - P_AP_DD_{BC} + P_BP_cD_{AD} - P_BP_DD_{AC} + P_cP_DD_{AB} - D_{ABCD}$
10) $P(ABcd) = P_AP_BP_cP_d + P_AD_{BCD} + P_BD_{ACD} - P_cD_{ABD} - P_dD_{ABC} + P_AP_BD_{CD} - P_AP_cD_{BD} - P_AP_dD_{BC} - P_BP_cD_{AD} - P_BP_dD_{AC} + P_cP_dD_{AB} + D_{ABCD}$
11) $P(aBcD) = P_aP_BP_cP_D - P_aD_{BCD} + P_BD_{ACD} - P_cD_{ABD} + P_DD_{ABC} - P_aP_BD_{CD} + P_aP_cD_{BD} - P_aP_DD_{BC} - P_BP_cD_{AD} + P_BP_DD_{AC} - P_cP_DD_{AB} + D_{ABCD}$
12) $P(aBcd) = P_aP_BP_cP_d + P_aD_{BCD} - P_BD_{ACD} + P_cD_{ABD} + P_dD_{ABC} + P_aP_BD_{CD} - P_aP_cD_{BD} - P_aP_dD_{BC} + P_BP_cD_{AD} + P_BP_dD_{AC} - P_cP_dD_{AB} - D_{ABCD}$
13) $P(AbcD) = P_AP_bP_cP_D + P_AD_{BCD} - P_bD_{ACD} - P_cD_{ABD} + P_DD_{ABC} - P_AP_bD_{CD} - P_AP_cD_{BD} + P_AP_DD_{BC} + P_bP_cD_{AD} - P_bP_DD_{AC} - P_cP_DD_{AB} + D_{ABCD}$
14) $P(Abcd) = P_AP_bP_cP_d - P_AD_{BCD} + P_bD_{ACD} + P_cD_{ABD} + P_dD_{ABC} + P_AP_bD_{CD} + P_AP_cD_{BD} + P_AP_dD_{BC} - P_bP_cD_{AD} - P_bP_dD_{AC} - P_cP_dD_{AB} - D_{ABCD}$
15) $P(abcD) = P_aP_bP_cP_D + P_aD_{BCD} + P_bD_{ACD} + P_cD_{ABD} - P_DD_{ABC} - P_aP_bD_{CD} - P_aP_cD_{BD} + P_aP_DD_{BC} - P_bP_cD_{AD} + P_bP_DD_{AC} + P_cP_DD_{AB} - D_{ABCD}$
16) $P(abcd) = P_aP_bP_cP_d - P_aD_{BCD} - P_bD_{ACD} - P_cD_{ABD} - P_dD_{ABC} + P_aP_bD_{CD} + P_aP_cD_{BD} + P_aP_dD_{BC} + P_bP_cD_{AD} + P_bP_dD_{AC} + P_cP_dD_{AB} + D_{ABCD}$

i) Constraints on the fourth-order LD coefficient:

$minD_{ABCD} \leq D_{ABCD} \leq maxD_{ABCD}$, where:

$minD_{ABCD} = \max[-P_0(ABCD), -P_0(aBCd), -P_0(AbCd), -P_0(abCD), -P_0(ABcd), -P_0(aBcD), -P_0(AbcD), -P_0(abcd)]$
and
$maxD_{ABCD} = \min[P_0(ABCd), P_0(aBCD), P_0(AbCD), P_0(abCd), P_0(ABcD), P_0(aBcd), P_0(Abcd), P_0(abcD)]$, where:

01) $P_0(ABCD) = P_AP_BP_CP_D + P_AD_{BCD} + P_BD_{ACD} + P_CD_{ABD} + P_DD_{ABC} + P_AP_BD_{CD} + P_AP_CD_{BD} + P_AP_DD_{BC} + P_BP_CD_{AD} + P_BP_DD_{AC} + P_CP_DD_{AB}$
02) $P_0(ABCd) = P_AP_BP_CP_d - P_AD_{BCD} - P_BD_{ACD} - P_CD_{ABD} + P_dD_{ABC} - P_AP_BD_{CD} - P_AP_CD_{BD} + P_AP_dD_{BC} - P_BP_CD_{AD} + P_BP_dD_{AC} + P_CP_dD_{AB}$
03) $P_0(aBCD) = P_aP_BP_CP_D + P_aD_{BCD} - P_BD_{ACD} - P_CD_{ABD} - P_DD_{AB\,C} + P_aP_BD_{CD} + P_aP_CD_{BD} + P_aP_DD_{BC} - P_BP_CD_{AD} - P_BP_DD_{AC} - P_CP_DD_{AB}$
04) $P_0(aBCd) = P_aP_BP_CP_d - P_aD_{BCD} + P_BD_{ACD} + P_CD_{ABD} - P_dD_{ABC} - P_aP_BD_{CD} - P_aP_CD_{BD} + P_aP_dD_{BC} + P_BP_CD_{AD} - P_BP_dD_{AC} - P_CP_dD_{AB}$
05) $P_0(AbCD) = P_AP_bP_CP_D - P_AD_{BCD} + P_bD_{ACD} - P_CD_{ABD} - P_DD_{ABC} + P_AP_bD_{CD} - P_AP_CD_{BD} - P_AP_DD_{BC} + P_bP_CD_{AD} + P_bP_DD_{AC} - P_CP_DD_{AB}$
06) $P_0(AbCd) = P_AP_bP_CP_d + P_AD_{BCD} - P_bD_{ACD} + P_CD_{ABD} - P_dD_{ABC} - P_AP_bD_{CD} + P_AP_CD_{BD} - P_AP_dD_{BC} - P_bP_CD_{AD} + P_bP_dD_{AC} - P_CP_dD_{AB}$
07) $P_0(abCD) = P_aP_bP_CP_D - P_aD_{BCD} - P_bD_{ACD} + P_CD_{ABD} + P_DD_{ABC} + P_aP_bD_{CD} - P_aP_CD_{BD} - P_aP_DD_{BC} - P_bP_CD_{AD} - P_bP_DD_{AC} + P_CP_DD_{AB}$
08) $P_0(abCd) = P_aP_bP_CP_d + P_aD_{BCD} + P_bD_{ACD} - P_CD_{ABD} + P_dD_{ABC} - P_aP_bD_{CD} + P_aP_CD_{BD} - P_aP_dD_{BC} + P_bP_CD_{AD} - P_bP_dD_{AC} + P_CP_dD_{AB}$
09) $P_0(ABcD) = P_AP_BP_cP_D - P_AD_{BCD} - P_BD_{ACD} + P_cD_{ABD} - P_DD_{ABC} - P_AP_BD_{CD} + P_AP_cD_{BD} - P_AP_DD_{BC} + P_BP_cD_{AD} - P_BP_DD_{AC} + P_cP_DD_{AB}$
10) $P_0(ABcd) = P_AP_BP_cP_d + P_AD_{BCD} + P_BD_{ACD} - P_cD_{ABD} - P_dD_{ABC} + P_AP_BD_{CD} - P_AP_cD_{BD} - P_AP_dD_{BC} - P_BP_cD_{AD} - P_BP_dD_{AC} + P_cP_dD_{AB}$
11) $P_0(aBcD) = P_aP_BP_cP_D - P_aD_{BCD} + P_BD_{ACD} - P_cD_{ABD} + P_DD_{ABC} - P_aP_BD_{CD} + P_aP_cD_{BD} - P_aP_DD_{BC} - P_BP_cD_{AD} + P_BP_DD_{AC} - P_cP_DD_{AB}$
12) $P_0(aBcd) = P_aP_BP_cP_d + P_aD_{BCD} - P_BD_{ACD} + P_cD_{ABD} + P_dD_{ABC} + P_aP_BD_{CD} - P_aP_cD_{BD} - P_aP_dD_{BC} + P_BP_cD_{AD} + P_BP_dD_{AC} - P_cP_dD_{AB}$
13) $P_0(AbcD) = P_AP_bP_cP_D + P_AD_{BCD} - P_bD_{ACD} - P_cD_{ABD} + P_DD_{ABC} - P_AP_bD_{CD} - P_AP_cD_{BD} + P_AP_DD_{BC} + P_bP_cD_{AD} - P_bP_DD_{AC} - P_cP_DD_{AB}$
14) $P_0(Abcd) = P_AP_bP_cP_d - P_AD_{BCD} + P_bD_{ACD} + P_cD_{ABD} + P_dD_{ABC} + P_AP_bD_{CD} + P_AP_cD_{BD} + P_AP_dD_{BC} - P_bP_cD_{AD} - P_bP_dD_{AC} - P_cP_dD_{AB}$
15) $P_0(abcD) = P_aP_bP_cP_D + P_aD_{BCD} + P_bD_{ACD} + P_cD_{ABD} - P_DD_{ABC} - P_aP_bD_{CD} - P_aP_cD_{BD} + P_aP_DD_{BC} - P_bP_cD_{AD} + P_bP_DD_{AC} + P_cP_DD_{AB}$
16) $P_0(abcd) = P_aP_bP_cP_d - P_aD_{BCD} - P_bD_{ACD} - P_cD_{ABD} - P_dD_{ABC} + P_aP_bD_{CD} + P_aP_cD_{BD} + P_aP_dD_{BC} + P_bP_cD_{AD} + P_bP_dD_{AC} + P_cP_dD_{AB}$

ii. Constraints on third-order LD coefficients:

$minD_{ABC} \leq D_{ABC} \leq maxD_{ABC}$, where:

$minD_{ABC} = \max[-P_0(ABC), -P_0(abC), -P_0(aBc), -P_0(Abc), m_1, m_2 \ldots\ldots\ldots, m_{12}]$ and
$maxD_{ABC} = \min[P_0(aBC), P_0(AbC), P_0(ABc), P_0(abc), M_1, M_2 \ldots\ldots\ldots, M_{12}]$, where:

$P_0(ABC) = P_AP_BP_C + P_AD_{BC} + P_BD_{AC} + P_CD_{AB}$
$P_0(aBC) = P_aP_BP_C + P_aD_{BC} - P_BD_{AC} - P_CD_{AB}$
$P_0(AbC) = P_AP_bP_C - P_AD_{BC} + P_bD_{AC} - P_CD_{AB}$
$P_0(abC) = P_aP_bP_C - P_aD_{BC} - P_bD_{AC} + P_CD_{AB}$
$P_0(ABc) = P_AP_BP_c - P_AD_{BC} - P_BD_{AC} + P_cD_{AB}$
$P_0(aBc) = P_aP_BP_c - P_aD_{BC} + P_BD_{AC} - P_cD_{AB}$
$P_0(Abc) = P_AP_bP_c + P_AD_{BC} - P_bD_{AC} - P_cD_{AB}$
$P_0(abc) = P_aP_bP_c + P_aD_{BC} + P_bD_{AC} + P_cD_{AB}$

and

Inequalities defining additional <u>lower bounds</u> for $D_{AB\,C}$ within the context of 4-allele haplotypes (total =12):
$m_1 = -D_{ABC} \leq P_AP_BP_CP_D + P_AP_bP_cP_d + (P_CP_D - P_cP_d)D_{AB} + (P_BP_D - P_bP_d)D_{AC} + (P_BP_C - P_bP_c)D_{AD} + P_A(D_{BD} + D_{BC} + D_{CD}) + D_{ABD} + D_{ACD}$
$m_2 = -D_{ABC} \leq P_AP_BP_CP_D + P_aP_BP_cP_d + (P_CP_D - P_cP_d)D_{AB} + (P_AP_D - P_aP_d)D_{BC} + (P_AP_C - P_aP_c)D_{BD} + P_B(D_{AC} + D_{AD} + D_{CD}) + D_{ABD} + D_{BCD}$
$m_3 = -D_{ABC} \leq P_AP_BP_CP_D + P_aP_bP_CP_d + (P_BP_D - P_bP_d)D_{AC} + (P_AP_D - P_aP_d)D_{BC} + (P_AP_B - P_aP_b)D_{CD} + P_C(D_{AB} + D_{AD} + D_{BD}) + D_{ACD} + D_{BCD}$
$m_4 = -D_{ABC} \leq P_AP_BP_CP_d + P_aP_bP_CP_D + (P_BP_d - P_bP_D)D_{AC} + (P_AP_d - P_aP_D)D_{BC} + (P_aP_b - P_AP_B)D_{CD} + P_C(D_{AB} - D_{AD} - D_{BD}) - D_{ACD} - D_{BCD}$

$m_5 = -D_{ABC} \le P_aP_BP_cP_d + P_aP_bP_CP_D + (P_BP_c - P_bP_C)D_{AD} + (P_BP_d - P_bP_D)D_{AC} + (P_CP_D - P_cP_d)D_{AB} + P_a(-D_{BC} - D_{BD} + D_{CD}) + D_{ABD} - D_{ACD}$
$m_6 = -D_{ABC} \le P_AP_bP_cP_d + P_aP_bP_CP_D + (P_CP_D - P_cP_d)D_{AB} + (P_AP_d - P_aP_D)D_{BC} + (P_AP_c - P_aP_C)D_{BD} + P_b(-D_{AC} - D_{AD} + D_{CD}) + D_{ABD} - D_{BCD}$
$m_7 = -D_{ABC} \le P_AP_BP_cP_d + P_aP_BP_cP_D + (P_CP_d - P_cP_D)D_{AB} + (P_AP_d - P_aP_D)D_{BC} + (P_aP_c - P_AP_C)D_{BD} + P_B(D_{AC} - D_{AD} - D_{CD}) - D_{ABD} - D_{BCD}$
$m_8 = -D_{ABC} \le P_aP_bP_CP_d + P_aP_BP_cP_D + (P_CP_d - P_cP_D)D_{AB} + (P_BP_D - P_bP_d)D_{AC} + (P_bP_C - P_BP_c)D_{AD} + P_a(-D_{BC} + D_{BD} - D_{CD}) - D_{ABD} + D_{ACD}$
$m_9 = -D_{ABC} \le P_AP_bP_cP_d + P_aP_BP_cP_D + (P_BP_D - P_bP_d)D_{AC} + (P_AP_d - P_aP_D)D_{BC} + (P_AP_b - P_aP_B)D_{CD} + P_c(-D_{AB} - D_{AD} + D_{BD}) - D_{ACD} + D_{BCD}$
$m_{10} = -D_{ABC} \le P_AP_BP_CP_d + P_AP_bP_cP_D + (P_CP_d - P_cP_D)D_{AB} + (P_BP_d - P_bP_D)D_{AC} + (P_bP_c - P_BP_C)D_{AD} + P_A(+D_{BC} - D_{BD} - D_{CD}) + D_{ABD} + D_{ACD}$
$m_{11} = -D_{ABC} \le P_aP_bP_CP_d + P_AP_bP_cP_D + (P_CP_d - P_cP_D)D_{AB} + (P_AP_D - P_aP_d)D_{BC} + (P_aP_C - P_AP_c)D_{BD} + P_b(-D_{AC} + D_{AD} - D_{CD}) - D_{ABD} + D_{BCD}$
$m_{12} = -D_{ABC} \le P_aP_BP_cP_d + P_AP_bP_cP_D + (P_aP_B - P_AP_b)D_{CD} + (P_AP_D - P_aP_d)D_{BC} + (P_BP_d - P_bP_D)D_{AC} + P_c(-D_{AB} + D_{AD} - D_{BD}) + D_{BCD} - D_{ACD}$

Inequalities defining additional upper bounds for D_{ABC} within the context of 4-allele haplotypes (total =12):
$M_1 = P_AP_bP_CP_D + P_aP_BP_CP_d + (P_bP_D - P_BP_d)D_{AC} + (P_aP_d - P_AP_D)D_{BC} + (P_AP_b - P_aP_B)D_{CD} + P_C(-D_{AB} + D_{AD} - D_{BD}) + D_{ACD} - D_{BCD}$
$M_2 = P_AP_BP_cP_D + P_aP_BP_CP_d + (P_cP_D - P_CP_d)D_{AB} + (P_aP_d - P_AP_D)D_{BC} + (P_AP_c - P_aP_C)D_{BD} + P_B(D_{AD} - D_{AC} - D_{CD}) + D_{ABD} - D_{BCD}$
$M_3 = P_aP_bP_cP_D + P_aP_BP_CP_d + (P_cP_D - P_CP_d)D_{AB} + (P_bP_D - P_BP_d)D_{AC} + (P_BP_C - P_bP_c)D_{AD} + P_a(-D_{CD} - D_{BD} + D_{BC}) + D_{ABD} + D_{ACD}$
$M_4 = P_aP_BP_CP_D + P_AP_bP_cP_d + (P_bP_d - P_BP_D)D_{AC} + (P_aP_D - P_AP_d)D_{BC} + (P_aP_B - P_AP_b)D_{CD} + P_C(-D_{AB} - D_{AD} + D_{BD}) - D_{ACD} + D_{BCD}$
$M_5 = P_AP_BP_cP_D + P_AP_bP_cP_d + (P_cP_D - P_CP_d)D_{AB} + (P_bP_d - P_BP_D)D_{AC} + (P_BP_c - P_bP_C)D_{AD} + P_A(-D_{BC} + D_{BD} - D_{CD}) + D_{ABD} - D_{ACD}$
$M_6 = P_aP_bP_cP_D + P_AP_bP_cP_d + (P_cP_D - P_CP_d)D_{AB} + (P_aP_D - P_AP_d)D_{BC} + (P_AP_C - P_aP_c)D_{BD} + P_b(-D_{AD} + D_{AC} - D_{CD}) + D_{ABD} + D_{BCD}$
$M_7 = P_aP_BP_CP_D + P_AP_BP_cP_d + (P_cP_d - P_CP_D)D_{AB} + (P_aP_D - P_AP_d)D_{BC} + (P_aP_C - P_AP_c)D_{BD} + P_B(-D_{AC} - D_{AD} + D_{CD}) - D_{ABD} + D_{BCD}$
$M_8 = P_AP_bP_CP_D + P_AP_BP_cP_d + (P_cP_d - P_CP_D)D_{AB} + (P_bP_D - P_BP_d)D_{AC} + (P_bP_C - P_BP_c)D_{AD} + P_A(-D_{BC} - D_{BD} + D_{CD}) - D_{ABD} + D_{ACD}$
$M_9 = P_aP_bP_cP_D + P_AP_BP_cP_d + (P_bP_D - P_BP_d)D_{AC} + (P_aP_D - P_AP_d)D_{BC} + (P_AP_B - P_aP_b)D_{CD} + P_c(D_{AB} - D_{AD} - D_{BD}) + D_{ACD} + D_{BCD}$
$M_{10} = P_aP_BP_CP_D + P_aP_bP_cP_d + (P_cP_d - P_CP_D)D_{AB} + (P_bP_d - P_BP_D)D_{AC} + (P_bP_c - P_BP_C)D_{AD} + P_a(D_{BC} + D_{BD} + D_{CD}) - D_{ACD} - D_{ABD}$
$M_{11} = P_AP_bP_CP_D + P_aP_bP_cP_d + (P_cP_d - P_CP_D)D_{AB} + (P_aP_d - P_AP_D)D_{BC} + (P_aP_c - P_AP_C)D_{BD} + P_b(D_{AC} + D_{AD} + D_{CD}) - D_{ABD} - D_{BCD}$
$M_{12} = P_AP_BP_cP_D + P_aP_bP_cP_d + (P_bP_d - P_BP_D)D_{AC} + (P_aP_d - P_AP_D)D_{BC} + (P_aP_b - P_AP_B)D_{CD} + P_c(D_{AB} + D_{AD} + D_{BD}) - D_{ACD} - D_{BCD}$

likewise for D_{ABD}. D_{ACD} and D_{BCD}.

iii. Constraints on second-order LD coefficients:

(1) $\min D_{AB} \le D_{AB} \le \max D_{AB}$, where:

$\min D_{AB} = \max[-P_AP_B, -P_aP_b, -m_1, -m_2, -m'_1, -m'_2]$ and
$\max D_{AB} = \min[P_AP_b, P_aP_B, M_1, M_2, M'_1, M'_2]$, with:

$m_1 = P_AP_BP_C + P_aP_bP_c + D_{AC} + D_{BC}$
$m_2 = P_AP_BP_c + P_aP_bP_C - D_{AC} - D_{BC}$
$M_1 = P_AP_bP_C + P_aP_BP_c + D_{AC} - D_{BC}$
$M_2 = P_AP_bP_c + P_aP_BP_C - D_{AC} + D_{BC}$

$m'_1 = P_AP_BP_D + P_aP_bP_d + D_{AD} + D_{BD}$
$m'_2 = P_AP_BP_d + P_aP_bP_D - D_{AB} - D_{BD}$
$M'_1 = P_AP_bP_D + P_aP_BP_d + D_{AD} - D_{BD}$
$M'_2 = P_AP_bP_d + P_aP_BP_D - D_{AB} + D_{BD}$

Likewise, for (2) D_{AC}, (3) D_{AD}, (4) D_{BC}, (5) D_{BD}, and (6) D_{CD} using the values of m'_1, listed in the following table:

Summary of constraints on second-order LD coefficients within the context of 4-allele (A, B, C, D) haplotypes

	Hap	LD	m_1/m'_1	m_2/m'_2	M_1/M'_1	M_2/M'_2
(1)	ABC	AB	$P_AP_BP_C + P_aP_bP_c + D_{AC} + D_{BC}$	$P_AP_BP_c + P_aP_bP_C - D_{AC} - D_{BC}$	$P_AP_bP_C + P_aP_BP_c + D_{AC} - D_{BC}$	$P_AP_bP_c + P_aP_BP_C - D_{AC} + D_{BC}$
(2)		AC	$P_AP_BP_C + P_aP_bP_c + D_{AB} + D_{BC}$	$P_AP_bP_C + P_aP_BP_c - D_{AB} - D_{BC}$	$P_AP_BP_c + P_aP_bP_C + D_{AB} - D_{BC}$	$P_AP_bP_c + P_aP_BP_C - D_{AB} + D_{BC}$
(3)		BC	$P_AP_BP_C + P_aP_bP_c + D_{AB} + D_{AC}$	$P_AP_bP_c + P_aP_BP_C - D_{AB} - D_{AC}$	$P_AP_BP_c + P_aP_bP_C + D_{AB} - D_{AC}$	$P_AP_bP_C + P_aP_BP_c - D_{AB} + D_{AC}$
(4)	ABD	AB	$P_AP_BP_D + P_aP_bP_d + D_{AD} + D_{BD}$	$P_AP_BP_d + P_aP_bP_D - D_{AD} - D_{BD}$	$P_AP_bP_D + P_aP_BP_d + D_{AD} - D_{BD}$	$P_AP_bP_d + P_aP_BP_D - D_{AD} + D_{BD}$
(5)		AD	$P_AP_BP_D + P_aP_bP_d + D_{AB} + D_{BD}$	$P_AP_bP_D + P_aP_BP_d - D_{AB} - D_{BD}$	$P_AP_BP_d + P_aP_bP_D + D_{AB} - D_{BD}$	$P_AP_bP_d + P_aP_BP_D - D_{AB} + D_{BD}$
(6)		BD	$P_AP_BP_D + P_aP_bP_d + D_{AB} + D_{AD}$	$P_AP_bP_d + P_aP_BP_D - D_{AB} - D_{AD}$	$P_AP_BP_d + P_aP_bP_D + D_{AB} - D_{AD}$	$P_AP_bP_D + P_aP_BP_d - D_{AB} + D_{AD}$
(7)	ACD	AC	$P_AP_CP_D + P_aP_cP_d + D_{AD} + D_{CD}$	$P_AP_CP_d + P_aP_cP_D - D_{AD} - D_{CD}$	$P_AP_cP_D + P_aP_CP_d + D_{AD} - D_{CD}$	$P_AP_cP_d + P_aP_CP_D - D_{AD} + D_{CD}$
(8)		AD	$P_AP_CP_D + P_aP_cP_d + D_{AC} + D_{CD}$	$P_AP_cP_D + P_aP_CP_d - D_{AC} - D_{CD}$	$P_AP_CP_d + P_aP_cP_D + D_{AC} - D_{CD}$	$P_AP_cP_d + P_aP_CP_D - D_{AC} + D_{CD}$
(9)		CD	$P_AP_CP_D + P_aP_cP_d + D_{AC} + D_{AD}$	$P_AP_cP_d + P_aP_CP_D - D_{AC} - D_{AD}$	$P_AP_CP_d + P_aP_cP_D + D_{AC} - D_{AD}$	$P_AP_cP_D + P_aP_CP_d - D_{AC} + D_{AD}$
(10)	BCD	BC	$P_BP_CP_D + P_bP_cP_d + D_{BD} + D_{CD}$	$P_BP_CP_d + P_bP_cP_D - D_{BD} - D_{CD}$	$P_BP_cP_D + P_bP_CP_d + D_{BD} - D_{CD}$	$P_BP_cP_d + P_bP_CP_D - D_{BD} + D_{CD}$
(11)		BD	$P_BP_CP_D + P_bP_cP_d + D_{BC} + D_{CD}$	$P_BP_cP_D + P_bP_CP_d - D_{BC} - D_{CD}$	$P_BP_CP_d + P_bP_cP_D + D_{BC} - D_{CD}$	$P_BP_cP_d + P_bP_CP_D - D_{BC} + D_{CD}$
(12)		CD	$P_BP_CP_D + P_bP_cP_d + D_{BC} + D_{BD}$	$P_BP_cP_d + P_bP_CP_D - D_{BC} - D_{BD}$	$P_BP_CP_d + P_bP_cP_D + D_{BC} - D_{BD}$	$P_BP_cP_D + P_bP_CP_d - D_{BC} + D_{BD}$

Note: within the context of 4-allele haplotypes, each second-order LD coefficient (AB, AC, BC, AD, BD, CD) is subject to two sets of constraints: one from each of two 3-allele haplotype contexts

References

[1] W.P. Robinson, M.A. Asmussen, G. Thomson, Three-locus systems impose additional constraints on pairwise disequilibria, Genetics. (1991).

[2] Jeffrey K. Conner and Daniel L. Hartl, A Primer of Ecological Genetics, Sinauer Associates, Sunderland (Massachusetts), (2005).

Supplementary File 5
Figures S1 and S2

Supplementary Figure S1.
Analysis of simulated mRNA expression/SNP genotype data sets (1)

A. Without addition of random error terms

One regulatory variant | Two regulatory variants

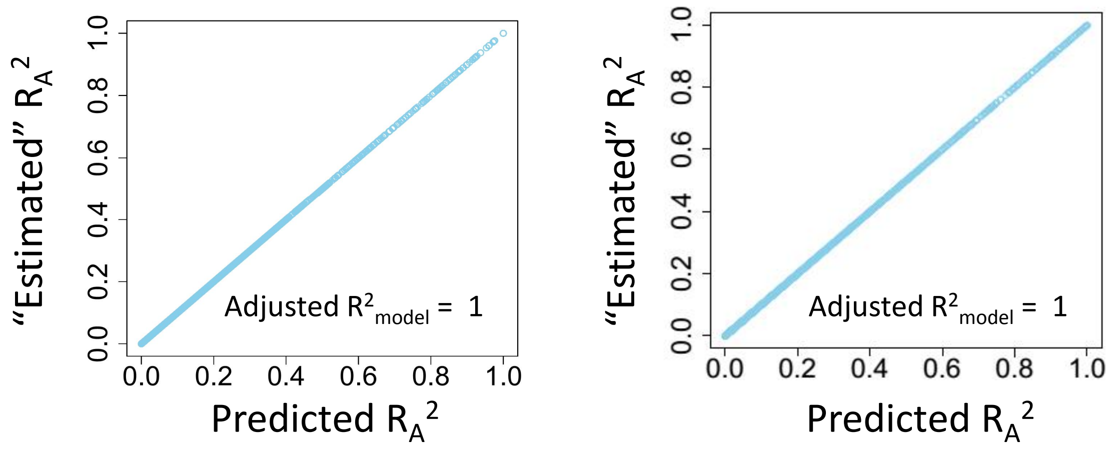

B. With addition of random error terms ∈ N[0, (1/5) (V_{total})]

One regulatory variant | Two regulatory variants

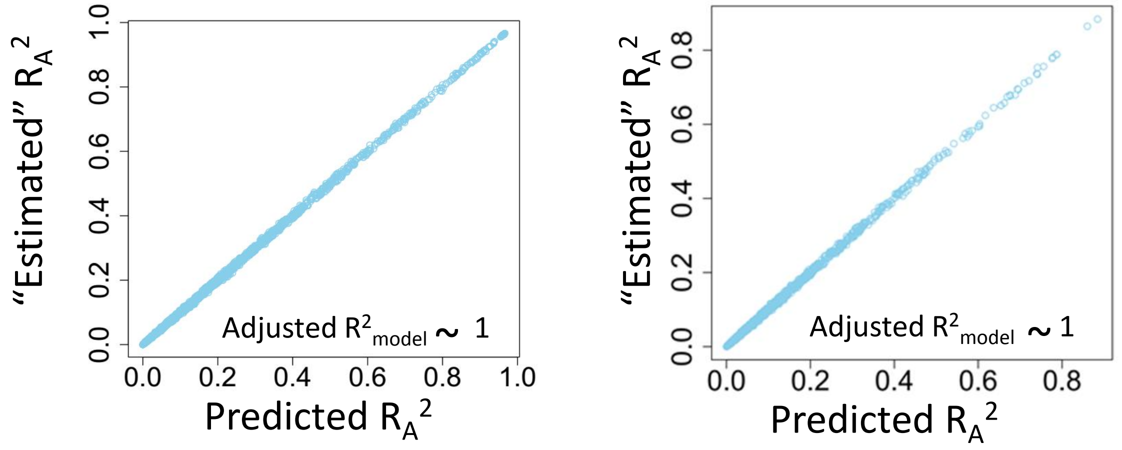

C. Without addition of random error terms

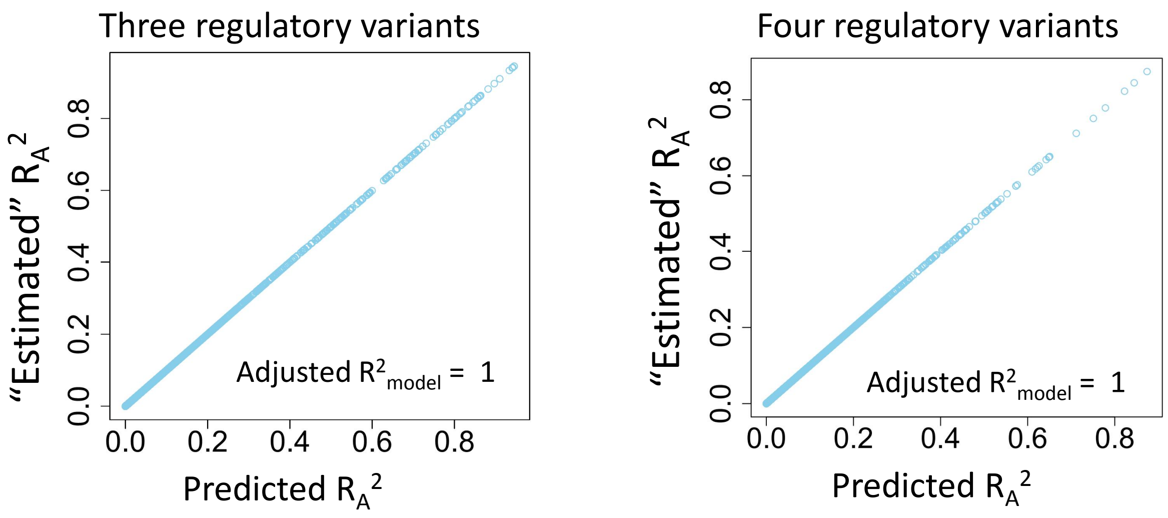

D. With addition of random error terms ∈ N[0, (1/5) (V_{total})]

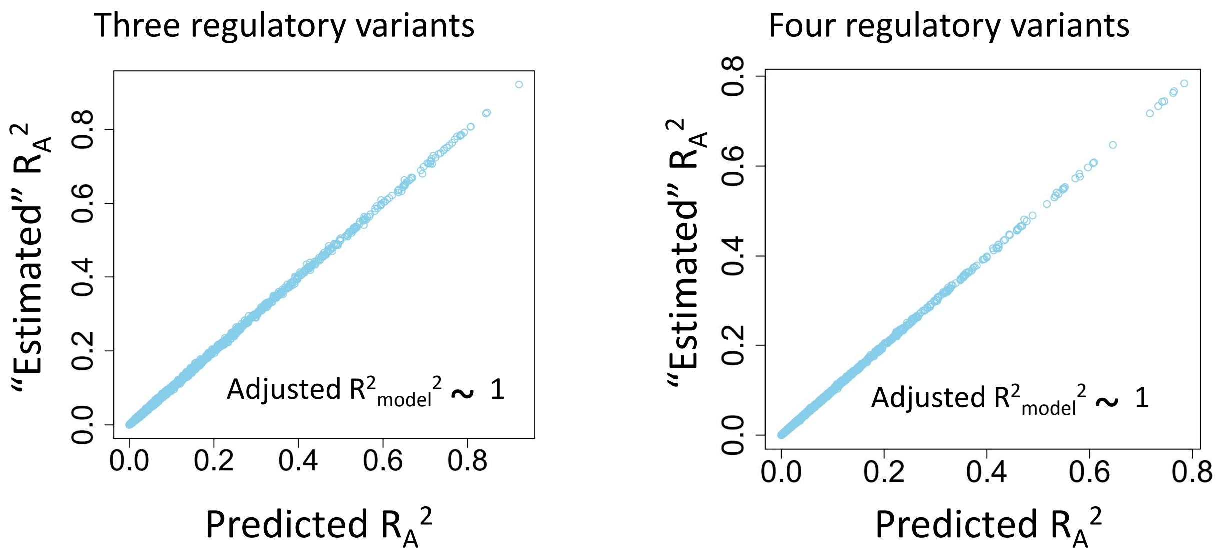

Legend

Comparisons of “estimated” and “predicted” coefficients of determination (R^2_A) based on simulated mRNA expression/genotype datasets comprising one non-regulatory SNP (designated SNP_A) and one, two, three or four bi-allelic regulatory variants (rVars: designated SNP_B, SNPc, SNP_D and SNP_E). A and C. Simulations without added error terms. B and D. Simulations with added random error terms. (See Main Text and **Supplementary File 3** for details.)

Supplementary Figure S2.
Analysis of simulated mRNA expression/SNP genotype data sets (2)

A

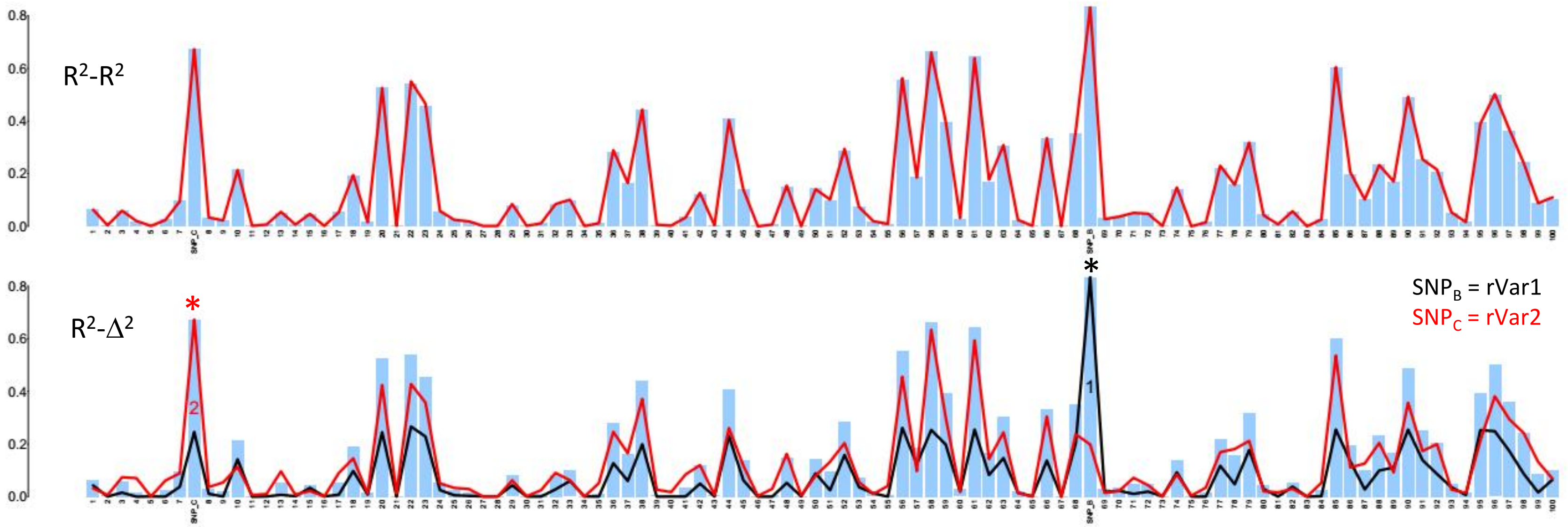

B

	Input values for construction of simulated mRNA expression/genotype data set						Input values for matrix equation calculations	
	Genotypic values			Range: mafs and LD coefficients			Pearson correlation coefficients	
	MM	Mm	mm	maf	Second-order D LD coefficients*	Third-order LD coefficients (D_{ABC})*	mRNA expression vs. genotype (R)	genotype vs. genotype (r)
SNP_A = nrVar	NA	0	NA	(0.05, 0.5)	D_{AB} = (-0.139, 0.136) D_{AC} = (-0.197, 0.238)	(-0.0294, 0.0288)	R_A = (0, 0.662)	r_{ab} = (0, 0.35) r_{ac} = (0, 0.62)
SNP_B = rVar1	-0.897	0	+0.897	P_b = 0.46	D_{BC} = 0.135		R_B = 0.844	r_{bc} = 0.3
SNP_C = rVar2	-0.639	0	+0.639	P_c = 0.43			R_C = 0.689	"

M = major allele; m = minor allele; maf = minor allele frequency; nrVar = non-regulatory variant; rVar = regulatory variant;
*Second- and third-order D LD coefficients are subject to the constraints defined in **Supplementary File 2, Section D.**

Legend

Analysis of R^2_A vs SNP and "SNP family" plots for a simulated mRNA expression/genotype data sets with two bi-allelic regulatory variants and 98 non-regulatory variants. **A.** Simulated mRNA expression/genotype data sets for 1000 individuals were constructed from a linear combination of the genotypes of the two regulatory variants (rVars). In each simulation, "estimated" single variable linear regression R^2 values based on the simulated data were plotted as bars above numbers enumerating the 98 non-regulatory SNPs (nrVar1-98: collectively designated SNP_A) and 2 regulatory variants (rVar1 =SNP_B and rVar2 = SNPc), with R^2 values for SNPs that attained nominally significant (i.e., $P < 0.05$) association with mRNA expression represented by blue bars. The order of the SNPs along the X-axes was arbitrarily assigned. For each simulation, the upper graph contains a red line plotting the values for "predicted" R^2_A, which were independently determined for each SNP by solving the matrix equation under the constraints described in the legend to Figure 1 for R^2_A. The lower graph plots the values of Δ^2 [= r^2 linkage disequilibrium (LD) coefficient] between the indicated non-regulatory SNP_A and each regulatory variant in the model, with $\Delta^2 = r^2 = 1$ for the regulatory variants scaled to the estimated R^2 value for that variant (marked with asterisks color-coded for each regulatory variant). Lines of different color delineate SNP "families" based on varying degrees of LD with each regulatory variant. **B.** Table listing input parameters and intermediate results of the simulation. The input parameters include genotypic values, minor allele frequencies (mafs), and second- and third-order LD coefficients for all simulated SNPs. Each regulatory variant was simulated from a combination of input parameters within permitted ranges defined by in **Supplementary File 3, Section D**. The input parameters for the two regulatory variants were fixed.

Supplementary File 6.
Analysis of *MTHFR* mRNA expression in 4BrainR-FCTX, -TCTX, -CERE and –PONS data sets

A. The *MTHFR* region of interest (ROI) on chromosome 1. The accompanying graphs show the results of analysis of MTHFR mRNA expression versus SNP genotypes for 450 genotyped or imputed SNPs located within in the indicated region of chromosome 1. (USCS genome browser image: GRCh37/hg19 version)

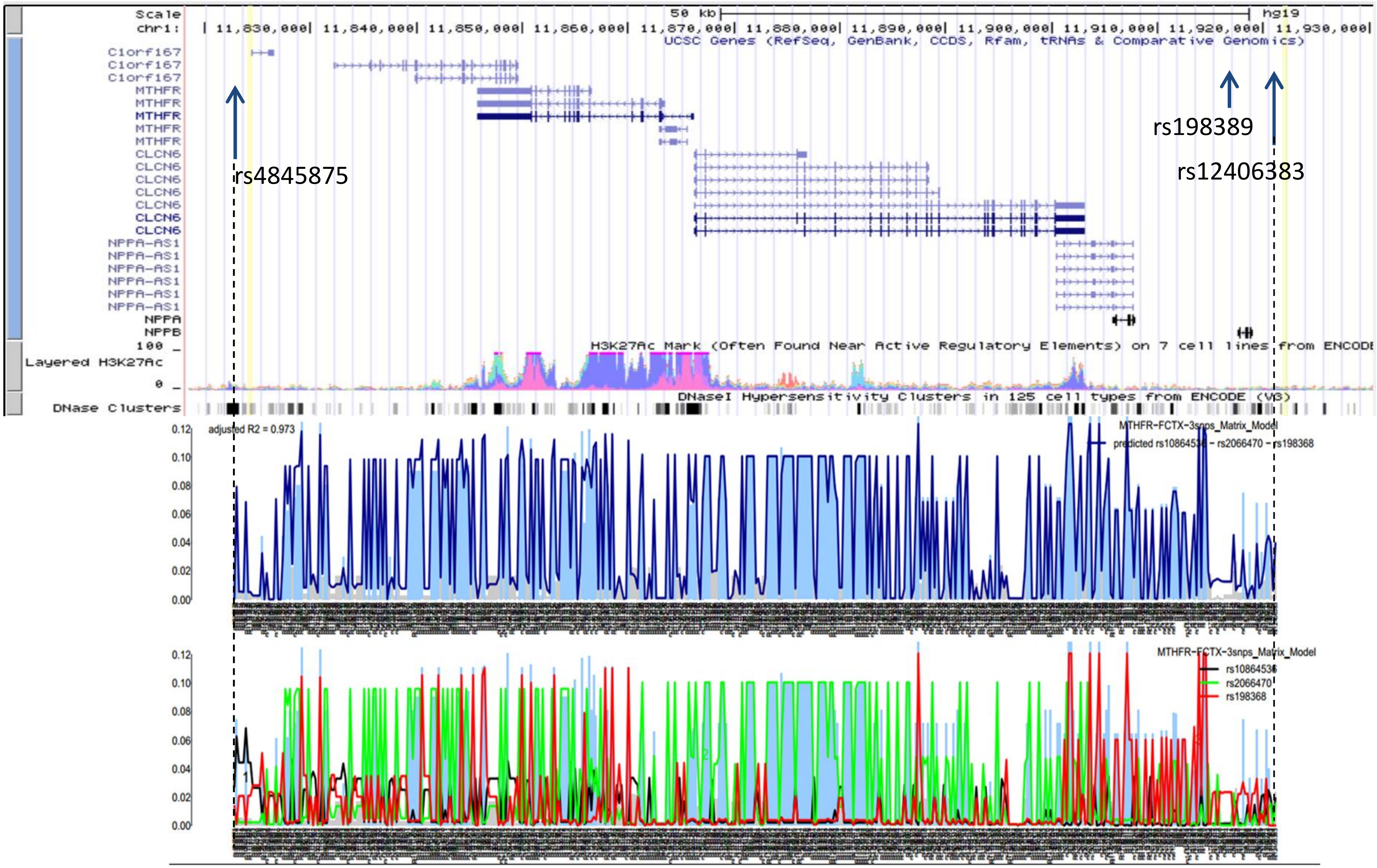

B. Top-ten 3-iSNP models selected by our matrix equation-based analysis of MTHFR mRNA expression in 4BrainR-450-FCTX

iSNP1	iSNP2	iSNP3	NRMSE	R2.adj	R2.T	R2B	R2C	R2D
rs10864536	rs2066470	rs198368	9.478352	0.973063	0.226887	0.06841	0.1006	0.1209
rs10864536	rs2066470	rs5064	9.603962	0.972068	0.229202	0.06841	0.1006	0.1177
rs10864536	rs2066470	rs198394	9.648272	0.973393	0.231698	0.06841	0.1006	0.129
rs10864536	rs114083374	rs198368	10.189346	0.973771	0.223078	0.06841	0.09437	0.1209
rs10864536	rs114083374	rs5064	10.239154	0.971277	0.225426	0.06841	0.09437	0.1177
rs10864536	rs17421560	rs198368	10.33589	0.970585	0.221238	0.06841	0.09384	0.1209
rs10864536	rs114083374	rs198394	10.341	0.970689	0.227871	0.06841	0.09437	0.129
rs4845876	rs2066470	rs198394	10.537001	0.961735	0.225484	0.07486	0.1006	0.129
rs10864536	rs17421560	rs5064	10.546302	0.968193	0.223473	0.06841	0.09384	0.1177
rs10864536	rs17421560	rs198394	10.706988	0.967635	0.22617	0.06841	0.09384	0.129

C. Differential ranking of the top-100 3-iSNP models based on normalized: i) [1/NRMSE], ii) adjusted R^2_{model} (R2.adj), iii) $[R^2_B + R^2_C + R^2_D]$ (sumR2BCD) , iv) R_M^2(R2.T) and v) sum of the 4 previous terms (sum-norms).
Arrows indicate the positions within these ranks of the top iSNP model (highlighted in yellow above).

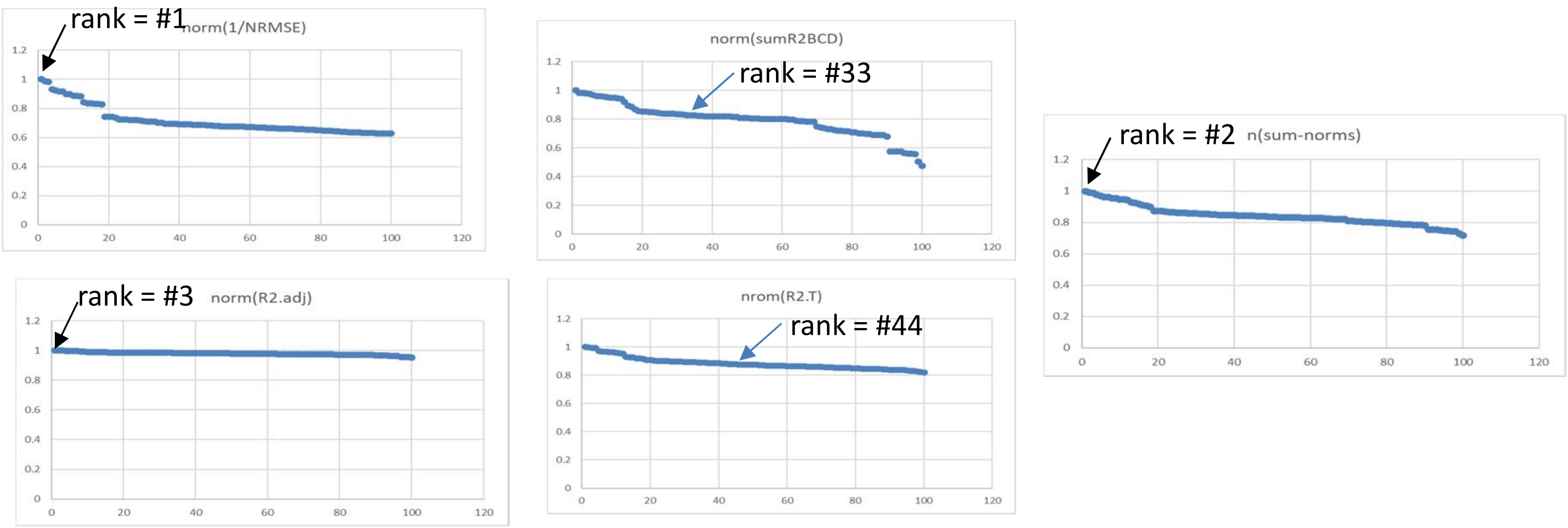

D. List of SNP family members for the top 3-iSNP model
(threshold for inclusion: r^2 LD coefficient with respect to iSNP > 0.6)

rs198368	rs2066470			rs10864536
rs198394	rs17421560	rs17037435	rs41307759	rs10864536
rs198403	rs3766747	rs149393118	rs12566905	rs4845876
rs198404	rs13306553	rs144795462	rs112862994	rs59911871
rs198407	rs35464336	rs143553702	rs139348312	rs9727993
rs171983	rs17037396	rs142218582	rs6665802	rs4845877
rs198412	rs2066470	rs34377596	rs6703535	rs4845878
rs198368	rs3753588	rs72640257	rs55890341	rs4845879
rs198370	rs7553194	rs72640258	rs55867221	rs11121825
rs5064	rs17367629	rs12564593	rs2151654	rs11121826
rs1106409	rs3753582	rs2076001	rs2184227	rs11559040
rs1572151	rs56221660	rs2076002	rs3820192	rs12076233
rs45495691	rs55814225	rs55994631	rs1537516	
rs17421462	rs6697335	rs2076004	rs1537515	
rs78329225	rs6691440	rs41275478	rs1537514	
rs80001635	rs6699827	rs41275488	rs72640221	
rs41301989	rs114540413	rs2236797	rs13306556	
rs41275460	rs145641960	rs72640262	rs2066462	
rs1889294	rs72640243	rs2075538	rs72638682	
rs75559897	rs72640244	rs72640267	rs55728339	
rs2184226	rs55851065	rs55788159	rs72638683	
rs55990971	rs12564002	rs114083374	rs72638684	
rs77157935	rs12565990	rs72638693	rs55929441	
rs41275464	rs55800247	rs72638696	rs36029635	
rs55686944	rs55738737	rs113980419	rs55990055	
	rs139569786	rs72638698	rs114951726	
	rs140586031	rs41275456	rs12131667	
	rs116422234	rs72638700		
	rs72640247	rs141567582		
	rs6699270	rs41275458		
	rs6701960	rs6541001		
	rs6687229	rs6688187		
	rs79448237	rs112131579		
	rs72640249	rs72640206		
	rs72640251	rs72640208		
	rs72640252	rs56313628		
	rs72640253	rs55738118		
	rs145628269	rs56260590		
	rs138252948	rs72640210		
	rs17376286	rs56001051		
	rs17037432	rs55685198		
	rs17037434	rs55967531		

E. List of SNP family members of RegulomeDB scores ≥ 3
(http://www.regulomedb.org/)

rs198368	r2	R2	regulome score
rs198404	1	0.129	1f (eQTL: MTHFR; monocytes)
rs198403	1	0.129	3a
rs1572151	0.91565761	0.1114	2b (footprinting:SATB1)2
rs17421462	0.91317136	0.112	3a
rs80001635	0.87366409	0.1112	2b (footprining: ZNF515)
rs1889294	0.87366409	0.1112	2b (footprinting: Spz1; PWM: Zfp281)

rs2066470	r2	R2	regulome score
rs72640252	1	0.1006	2b (footprinting: Freac-3)
rs72640253	1	0.1006	2b (footprinting: Freac-3)
rs2075538	1	0.1006	1d (eQTL: MTHFR, CLCN6; monocytes; PWM: NRSF)
rs72640210	0.95472	0.0905	2b (footprinting: FXRinvertedrepeat1)
rs55867221	0.95472	0.0905	2a (footprinting:NRSF)
rs72640221	0.95472	0.0905	3a
rs55929441	0.95336	0.08038	3a

rs10864536	r2	R2	regulome score
rs11559040	0.63872064	0.03607	3a

r2 = r^2 LD coefficient with respect to family iSNP
R2= R^2 = coefficient of determination for individual SNPs

F. Top 3-iSNP models for *MTHFR*: 4BrainR-450-FCTX and -TCTX

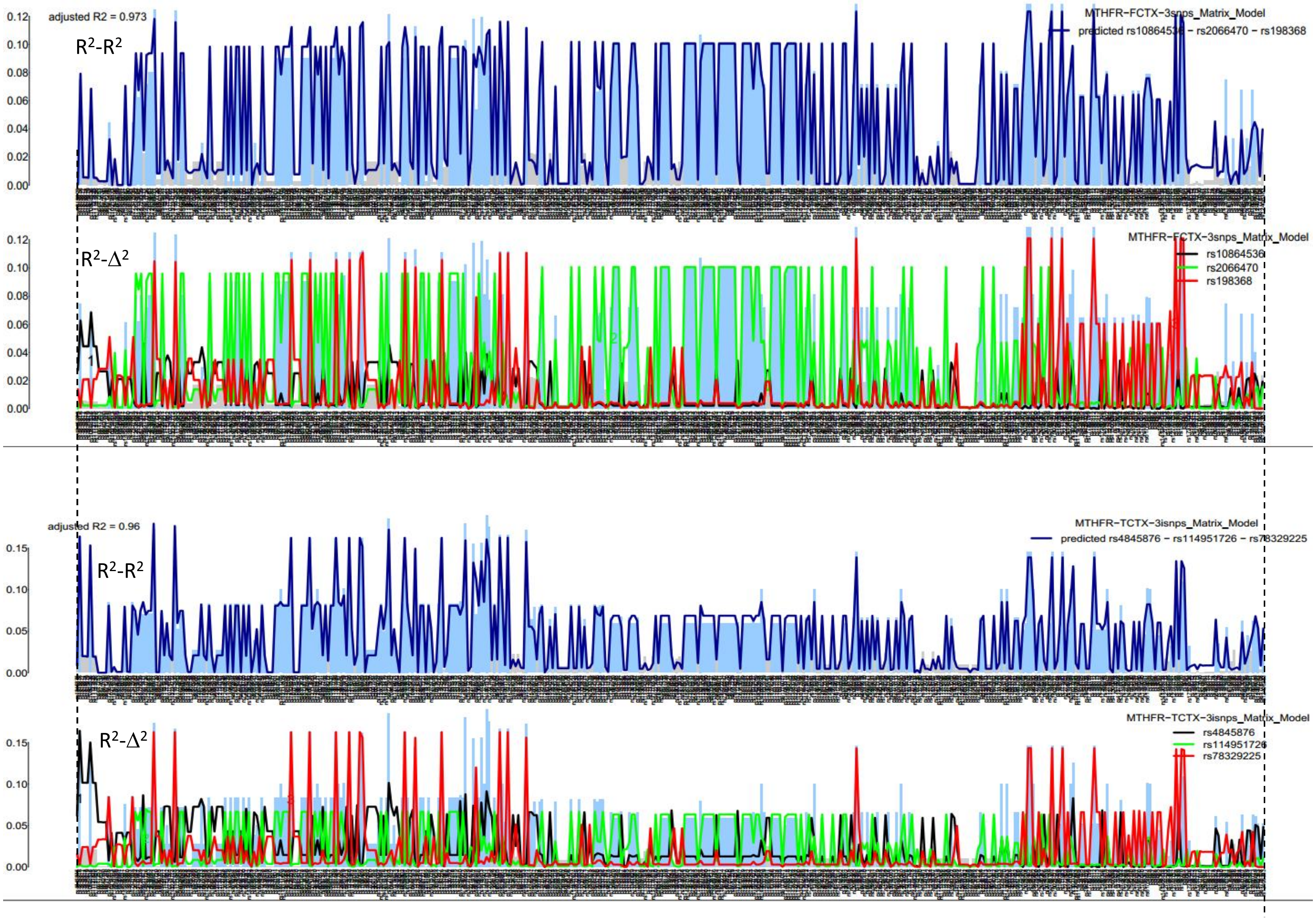

G. Top 3-iSNP models for MTHFR in 4BrainR-450-CERE and -PONS

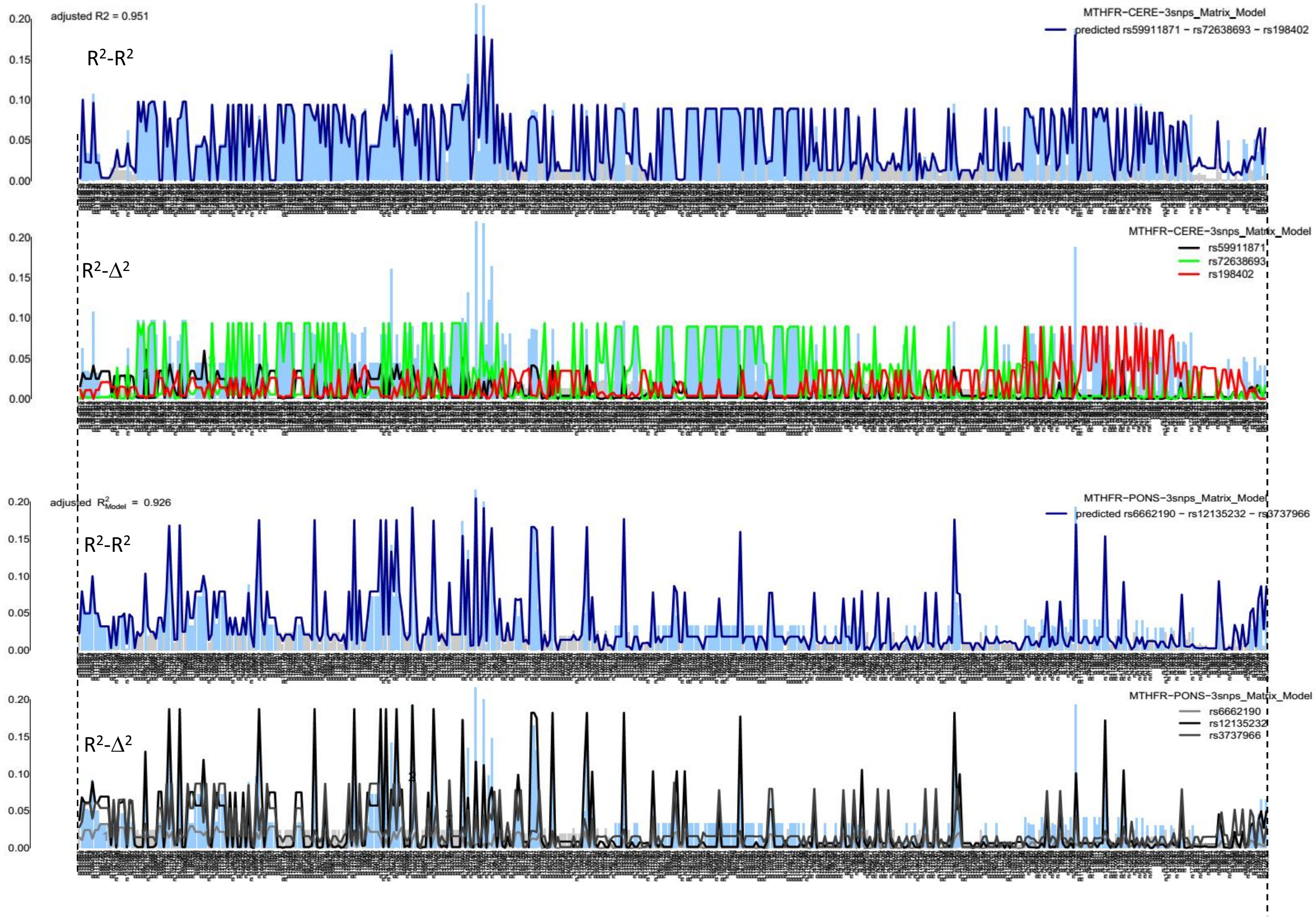

H. FCTX iSNP Family 1 SNPs (threshold for inclusion: $r^2 > 0.4$ with respect to iSNP rs198368)

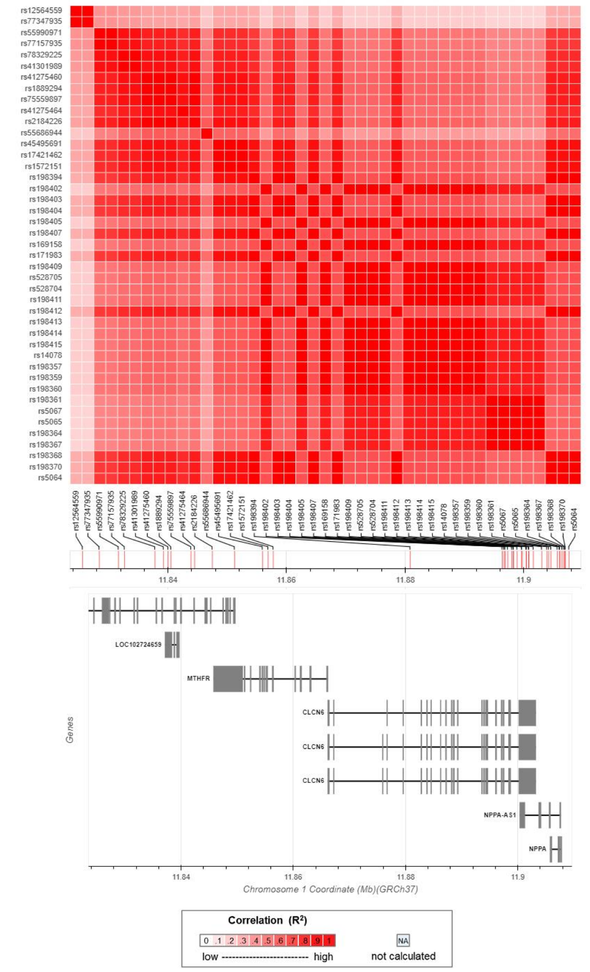

LD Structure/locations of family 1 SNPs
In CEU dataset (https:// ldlink.nci.nih.gov/)

rs198368	vlookup TCTX (0.6)	vlookup CERE(0.6)	vlookup CERE (0.4<CERE<0.6)
rs12564559			
rs77347935			
rs55990971	O		
rs77157935	O		
rs78329225	O		O
rs41301989	O		O
rs41275460	O		O
rs1889294	O		O
rs75559897	O		O
rs41275464	O		
rs2184226	O		O
rs55686944	O		
rs45495691	O		O
rs17421462	O		O
rs1572151	O		O
rs198394	O		O
rs198402		O	
rs198403	O		O
rs198404	O		O
rs198405		O	
rs198407	O		O
rs169158		O	
rs171983	O		O
rs198409		O	
rs528705		O	
rs528704		O	
rs198411		O	
rs198412	O		O
rs198413		O	
rs198414		O	
rs198415		O	
rs14078		O	
rs198357		O	
rs198359		O	
rs198360		O	
rs198361		O	
rs5067		O	
rs5065		O	
rs198364		O	
rs198367		O	
rs198368	O		O
rs198370	O		O
rs5064	O		O

The observed distributions of common SNPs among iSNP families in FCTX, TCT and CERE suggest the existence of two distinct regulatory variants within the FCTX iSNP 3 family

I. FCTX iSNP Family 2 SNPs (threshold for inclusion: $r^2 > 0.4$ with respect to iSNP rs2066470)

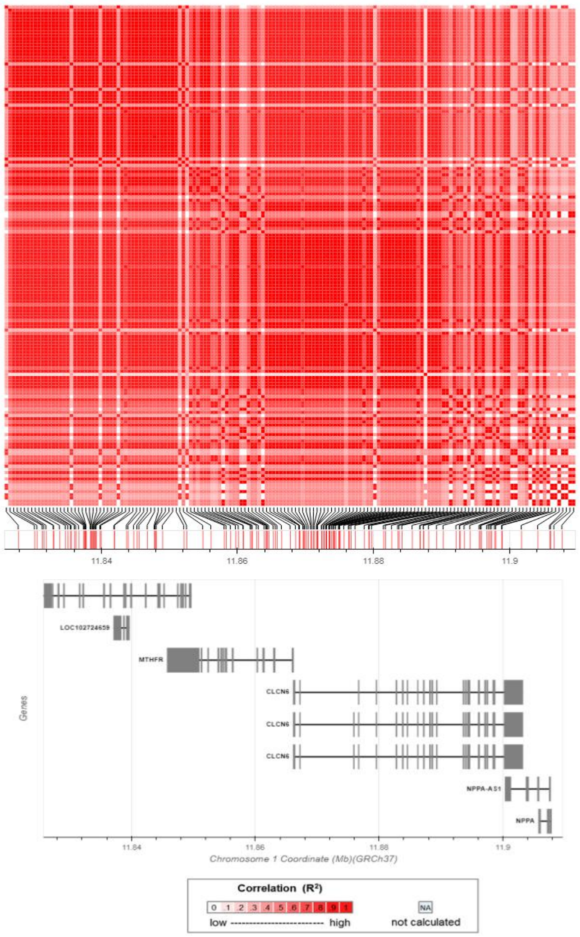

LD Structure/locations of family 2 SNPs
In CEU dataset (https:// ldlink.nci.nih.gov/)

	rs2066470	TCTX rs114951726	CERE rs72638693
FCTX	rs17421560	O	O
iSNP	rs3766747	O	O
family 2	rs13306553	O	O
	rs35464336	O	O
	rs17037396	O	O
	rs2066470	O	O
	rs3753588	O	O
	rs7553194	O	O
	rs17367629	O	O
	rs3753582	O	O
	rs56221660	O	O
	rs55814225	O	O
	rs6697335	O	O
	rs6691440	O	O
	rs6699827	O	O
	rs114540413	O	O
	rs145641960	O	O
	rs72640243	O	O
	rs72640244	O	O
	rs55851065	O	O
	rs12564002	O	O
	rs12565990	O	O
	rs55800247	O	O
	rs55738737	O	O
	rs139569786	O	O
	rs140586031	O	O
	rs116422234	O	O
	rs72640247	O	O
	rs6699270	O	O
	rs6701960	O	O
	rs6687229	O	O
	rs79448237	O	O
	rs72640249	O	O
	rs72640251	O	O
	rs72640252	O	O
	rs72640253	O	O
	rs145628269	O	O
	rs138252948	O	O
	rs17376286	O	O
	rs17037432	O	O
	rs17037434	O	O
	rs17037435	O	O
	rs149393118	O	O
	rs144795462	O	O
	rs143553702	O	O
	rs142218582	O	O
	rs34377596	O	O
	rs72640257	O	O
	rs72640258	O	O
	rs12564593	O	O
	rs2076001	O	O
	rs2076002	O	O
	rs55994631	O	O
	rs2076004	O	O

rs41275478	O	O
rs41275488	O	O
rs2236797	O	O
rs72640262	O	O
rs2075538	O	O
rs72640267	O	O
rs55788159	O	O
rs114083374	O	O
rs72638693	O	O
rs72638696	O	O
rs113980419	O	O
rs72638698	O	O
rs41275456	O	O
rs72638700	O	O
rs141567582	O	O
rs41275458	O	O
rs6541001	O	O
rs6688187	O	O
rs112131579	O	O
rs72640206	O	O
rs72640208	O	O
rs56313628	O	O
rs55738118	O	O
rs56260590	O	O
rs72640210	O	O
rs56001051	O	O
rs55685198	O	O
rs55967531	O	O
rs41307759	O	O
rs12566905	O	O
rs112862994	O	O
rs139348312	O	O
rs6665802	O	O
rs6703535	O	O
rs55890341	O	O
rs55867221	O	O
rs2151654	O	O
rs2184227	O	O
rs3820192	O	O
rs1537516	O	O
rs1537515	O	O
rs1537514	O	O
rs72640221	O	O
rs13306556	O	O
rs2066462	O	O
rs72638682	O	O
rs55728339	O	O
rs72638683	O	O
rs72638684	O	O
rs55929441	O	O
rs36029635	O	O
rs55990055	O	O
rs114951726	O	O
rs12131667	O	O

rs17037425	O	O
rs12561919	O	O
rs17037388	O	O
rs45553335	O	O
rs17037390	O	O
rs17367504	O	O
rs3753584	O	O
rs13306561	O	O
rs17037427	O	O
rs17037429	O	O
rs41275472	O	O
rs2050265	O	O
rs6699618	O	O
rs6669371	O	O
rs12567119	O	O
rs12567136	O	O
rs56153133	O	O
rs7537765	O	O
rs2236796	O	O
rs17037452	O	O
rs55857306	O	O
rs45603435	O	O
rs148411205	O	O
rs72640211	O	O
rs41307757	O	O
rs17375901	O	O
rs45439601	O	O
rs142005893	O	O
rs17376328	O	O
rs72640260	O	O
rs41275494	O	O
rs143800963	O	O
rs55892892	O	O
rs55741089	O	O
rs41275502	O	O
rs61764044	O	O
rs5068	O	O
rs3753586		
rs3753585		
rs3737965		
rs75747410		
rs2076003		
rs77072136		
rs41275484		
rs79811212		
rs41275500		
rs2075539		
rs2272803		
rs7552330		
rs141308438		
rs5063		
rs17037397		O
rs145488887	O	O
rs114941496	O	O
rs3737967		O
rs2274976		O
rs61757273		
rs112521149		

The lists of iSNP family 2 SNPs in FCTX, TCTX and CERE are nearly identical, suggesting that one or more underlying regulatory variants are active in these brain regions. By contrast, 3 iSNP families identified in the top model for PONS showed no SNPs in common with FCTX iSNP family 2 (at the 0.4 inclusion threshold).

J. FCTX iSNP family 3 SNPs (inclusion range: 0. 439 < $r^2 \leq 1$ with respect to rs10864536)

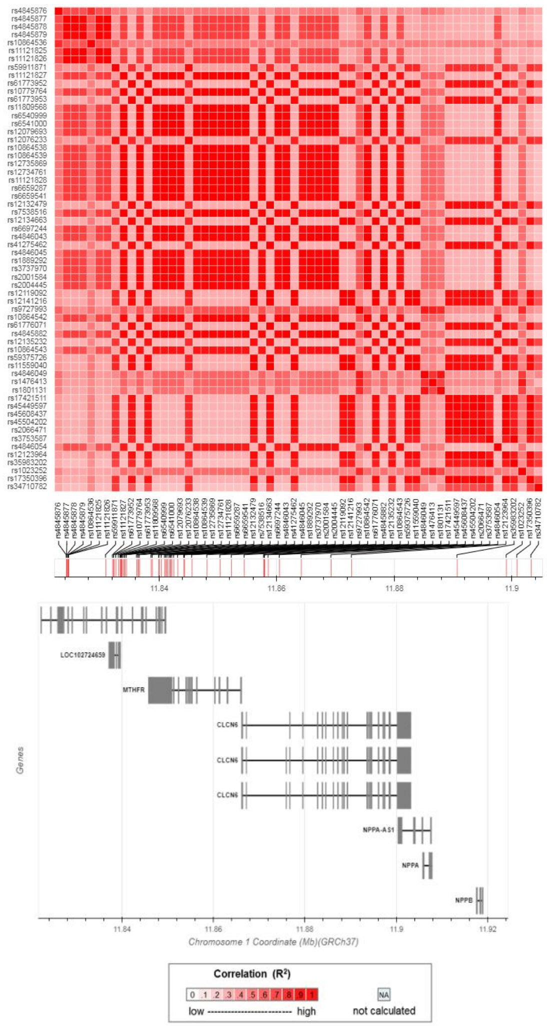

LD structure/locations of family 3 SNPs in CEU dataset ([https:// ldlink.nci.nih.gov/](https://ldlink.nci.nih.gov/))

SNP	SNP	0.2 < TCTX rs4845876	0.6 < TCTX rs4845876	0.6 < CERE rs59911871	0.6 < PONS1 rs12135232	0.6 < PONS2 rs3737966	0.6 < PONS3 rs6662190
rs4845876	rs4845876	o	o				
rs4845877	rs4845877	o	o				o
rs4845878	rs4845878	o	o				o
rs4845879	rs4845879	o	o				o
rs10864536	rs10864536	o	o	o			
rs11121825	rs11121825	o	o				o
rs11121826	rs11121826	o	o				o
rs59911871	rs59911871	o		o	o		
rs11121827	rs11121827	o				o	
rs61773952	rs61773952	o		o	o		
rs10779764	rs10779764	o				o	
rs61773953	rs61773953	o		o	o		
rs11809568	rs11809568	o				o	
rs6540999	rs6540999	o				o	
rs6541000	rs6541000	o				o	
rs12079693	rs12079693	o				o	
rs12076233	rs12076233	o		o	o		
rs10864538	rs10864538	o				o	
rs10864539	rs10864539	o				o	
rs12735869	rs12735869	o				o	
rs12734761	rs12734761	o				o	
rs11121828	rs11121828	o				o	
rs6659287	rs6659287	o				o	
rs6659541	rs6659541	o				o	
rs12132479	rs12132479	o		o	o		
rs7538516	rs7538516	o				o	
rs12134663	rs12134663	o		o	o		
rs6697244	rs6697244	o				o	
rs4846043	rs4846043	o				o	
rs41275462	rs41275462	o		o	o		
rs4846045	rs4846045	o				o	
rs1889292	rs1889292	o				o	
rs3737970	rs3737970	o				o	
rs2001584	rs2001584	o				o	
rs2004445	rs2004445	o				o	
rs12119092	rs12119092	o		o	o		
rs12141216	rs12141216	o		o	o		
rs9727993	rs9727993	o	o	o		o	
rs10864542	rs10864542	o				o	
rs61776071	rs61776071	o		o	o		
rs4845882	rs4845882	o				o	
rs12135232	rs12135232	o		o	o		
rs10864543	rs10864543	o				o	
rs59375726	rs59375726	o		o	o		
rs11559040	rs11559040	o		o	o		
rs4846049	rs4846049	o					
rs1476413	rs1476413	o					
rs1801131	rs1801131	o					
rs17421511	rs17421511	o		o	o		
rs45449597	rs45449597	o		o	o		
rs45608437	rs45608437	o		o	o		
rs45504202	rs45504202	o		o	o		
rs2066471	rs2066471	o		o	o		
rs3753587	rs3753587	o		o	o		
rs4846054	rs4846054	o				o	
rs12123964	rs12123964	o		o	o		
rs35983202	rs35983202	o		o	o		
rs1023252	rs1023252	o					
rs17350396	rs17350396	o		o	o		
rs34710782	rs34710782	o		o			

Common Family 3 SNPs in FCTX,TCTX, CERE and PONS iSNP families
(Threshold for inclusion: r2 > 0.6 with respect to family iSNPs)
Color code for FCTX family 3 SNP r2 > 0.9 r2 > 0.6 r2>0.4 r2>0.2
Figure continues on next slide

J. FCTX iSNP family 3 SNPs (inclusion range: 0. 2 < r2 ≤ 0.439 with respect to rs10864536)

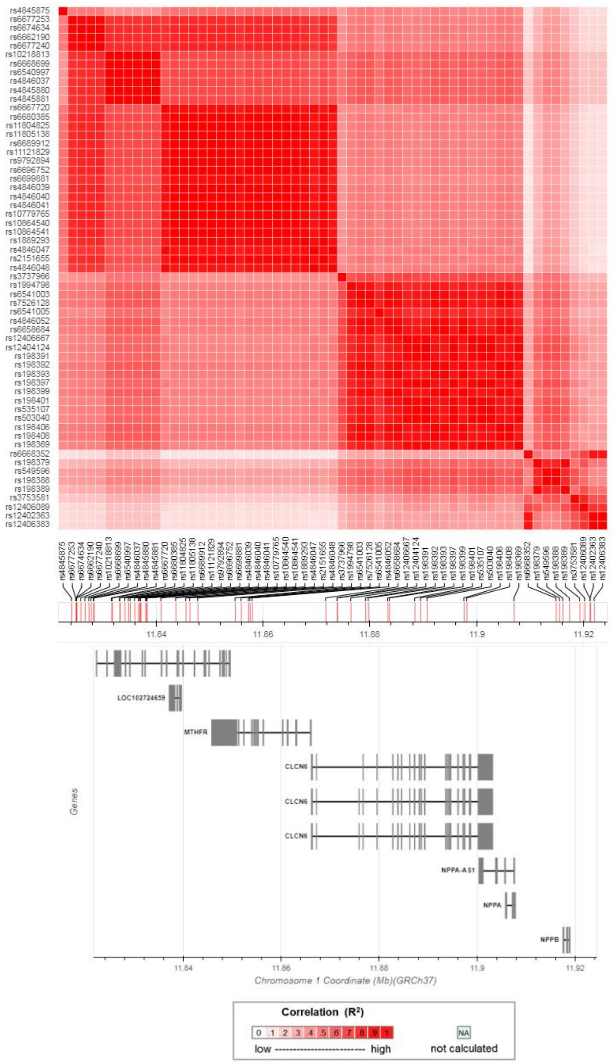

SNP	SNP	0.2 < TCTX rs4845876	0.6 < TCTX rs4845876	0.6 < CERE rs59911871	0.6 < PONS1 rs12135232	0.6 < PONS2 rs3737966	0.6 < PONS3 rs6662190
rs4845875	rs4845875	O					
rs6677253	rs6677253	O					O
rs6674634	rs6674634	O					O
rs6662190	rs6662190	O					O
rs6677240	rs6677240	O					O
rs10218813	rs10218813	O					O
rs6668699	rs6668699	O					O
rs6540997	rs6540997	O					O
rs4846037	rs4846037	O					O
rs4845880	rs4845880	O					O
rs4845881	rs4845881	O					O
rs6667720	rs6667720	O					O
rs6680385	rs6680385	O					O
rs11804825	rs11804825	O					O
rs11805138	rs11805138	O					O
rs6689912	rs6689912	O					O
rs11121829	rs11121829	O					O
rs9792894	rs9792894	O					O
rs6696752	rs6696752	O					O
rs6699881	rs6699881	O					O
rs4846039	rs4846039	O					O
rs4846040	rs4846040	O					O
rs4846041	rs4846041	O					O
rs10779765	rs10779765	O					O
rs10864540	rs10864540	O					O
rs10864541	rs10864541	O					O
rs1889293	rs1889293	O					O
rs4846047	rs4846047	O					O
rs2151655	rs2151655	O					O
rs4846048	rs4846048	O					O
rs3737966	rs3737966	O				O	
rs1994798	rs1994798	O				O	
rs6541003	rs6541003	O				O	
rs7526128	rs7526128	O				O	
rs6541005	rs6541005	O				O	
rs4846052	rs4846052	O				O	
rs6658684	rs6658684	O				O	
rs12406667	rs12406667	O				O	
rs12404124	rs12404124	O				O	
rs198391	rs198391	O				O	
rs198392	rs198392	O				O	
rs198393	rs198393	O				O	
rs198397	rs198397	O				O	
rs198399	rs198399	O				O	
rs198401	rs198401	O				O	
rs535107	rs535107	O				O	
rs503040	rs503040	O				O	
rs198406	rs198406	O				O	
rs198408	rs198408	O				O	
rs198369	rs198369	O				O	
rs6668352	rs6668352	O					
rs198379	rs198379	O					
rs549596	rs549596						
rs198388	rs198388	O					
rs198389	rs198389	O					
rs3753581	rs3753581	O					
rs12406089	rs12406089	O					
rs12402363	rs12402363	O					
rs12406383	rs12406383	O					

The observed distributions of SNPs among iSNP families in FCTX,TCTX and CERE (**O**,**O**,**O**,**O**) suggest the existence of <u>four</u> distinct regulatory variants within the FCTX iSNP 3 family.

K. Analysis of a subset of 139 non-redundant SNPs from the same chromosome ROI yielded nearly identical results as the full 450 SNP data set with respect to the number and characteristics of identified iSNP families (here, colored-coded as in preceding figures).

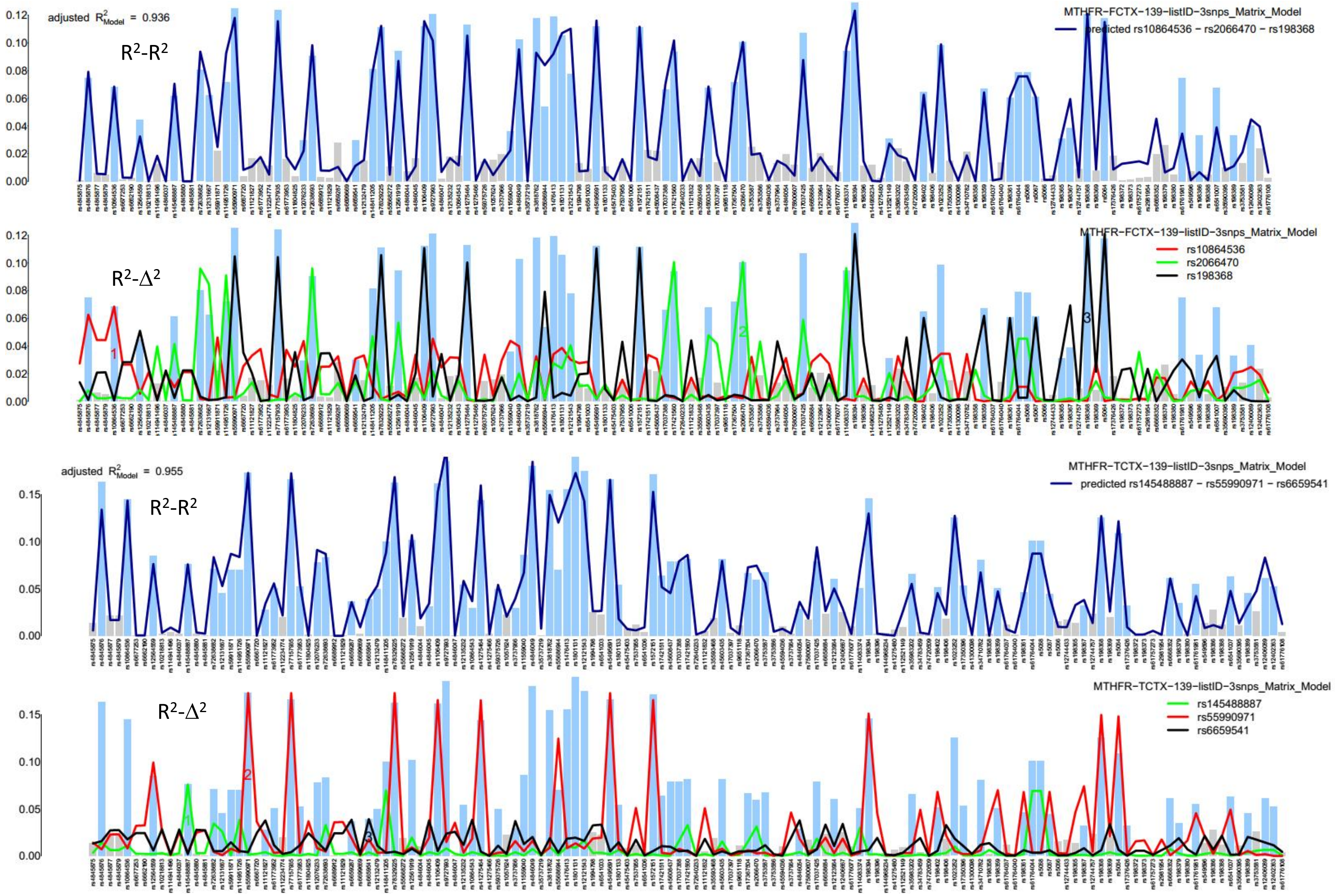

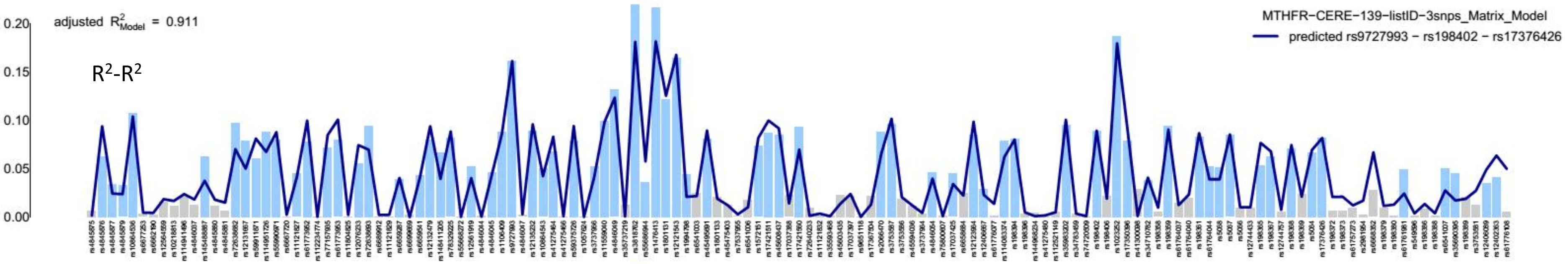
adjusted R^2_{Model} = 0.911
R^2-R^2
MTHFR-CERE-139-listID-3snps_Matrix_Model
predicted rs9727993 - rs198402 - rs17376426

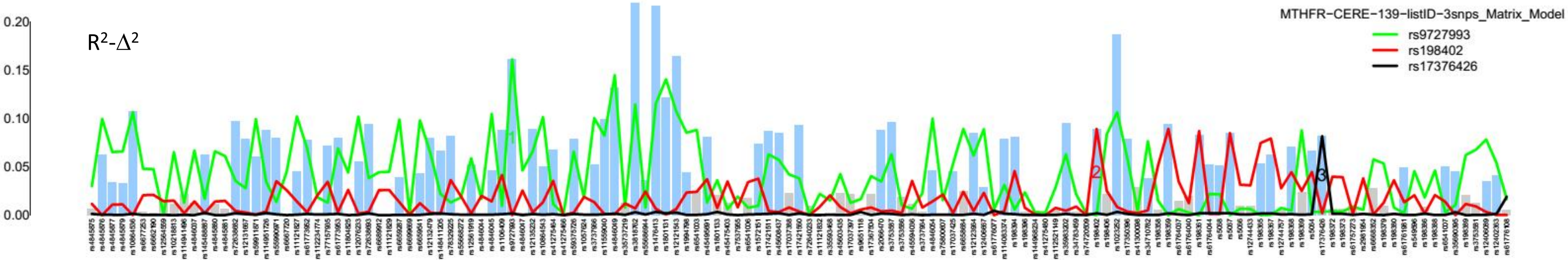
R^2-Δ^2
MTHFR-CERE-139-listID-3snps_Matrix_Model
rs9727993
rs198402
rs17376426

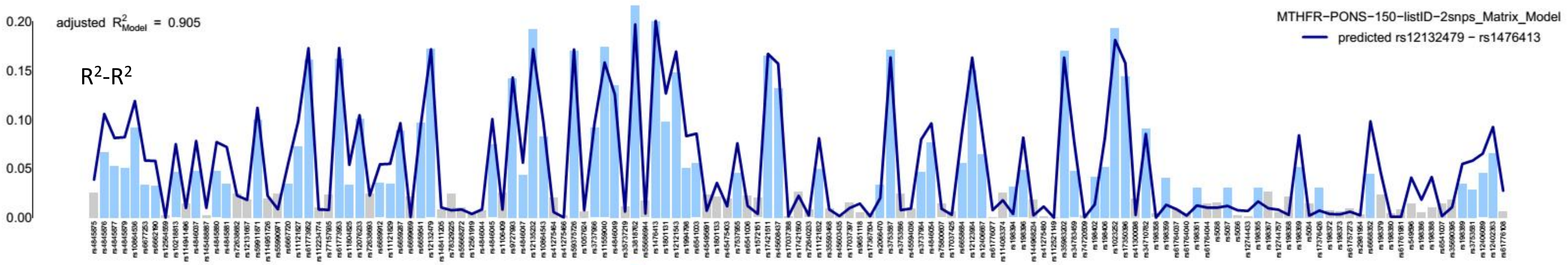
adjusted R^2_{Model} = 0.905
R^2-R^2
MTHFR-PONS-150-listID-2snps_Matrix_Model
predicted rs12132479 - rs1476413

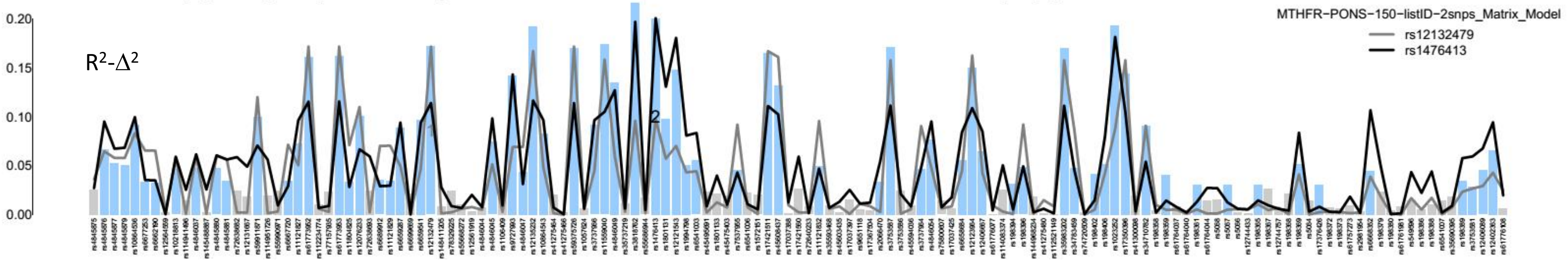
R^2-Δ^2
MTHFR-PONS-150-listID-2snps_Matrix_Model
rs12132479
rs1476413

Supplementary File 7.
Analysis of *MTHF* mRNA expression in lymphoblastoma cell lines (LCLs)

A. The *MTHF* region of interest (ROI): Chr1:11824133 - 11922298 (GRChR37/hg19 coordinates)

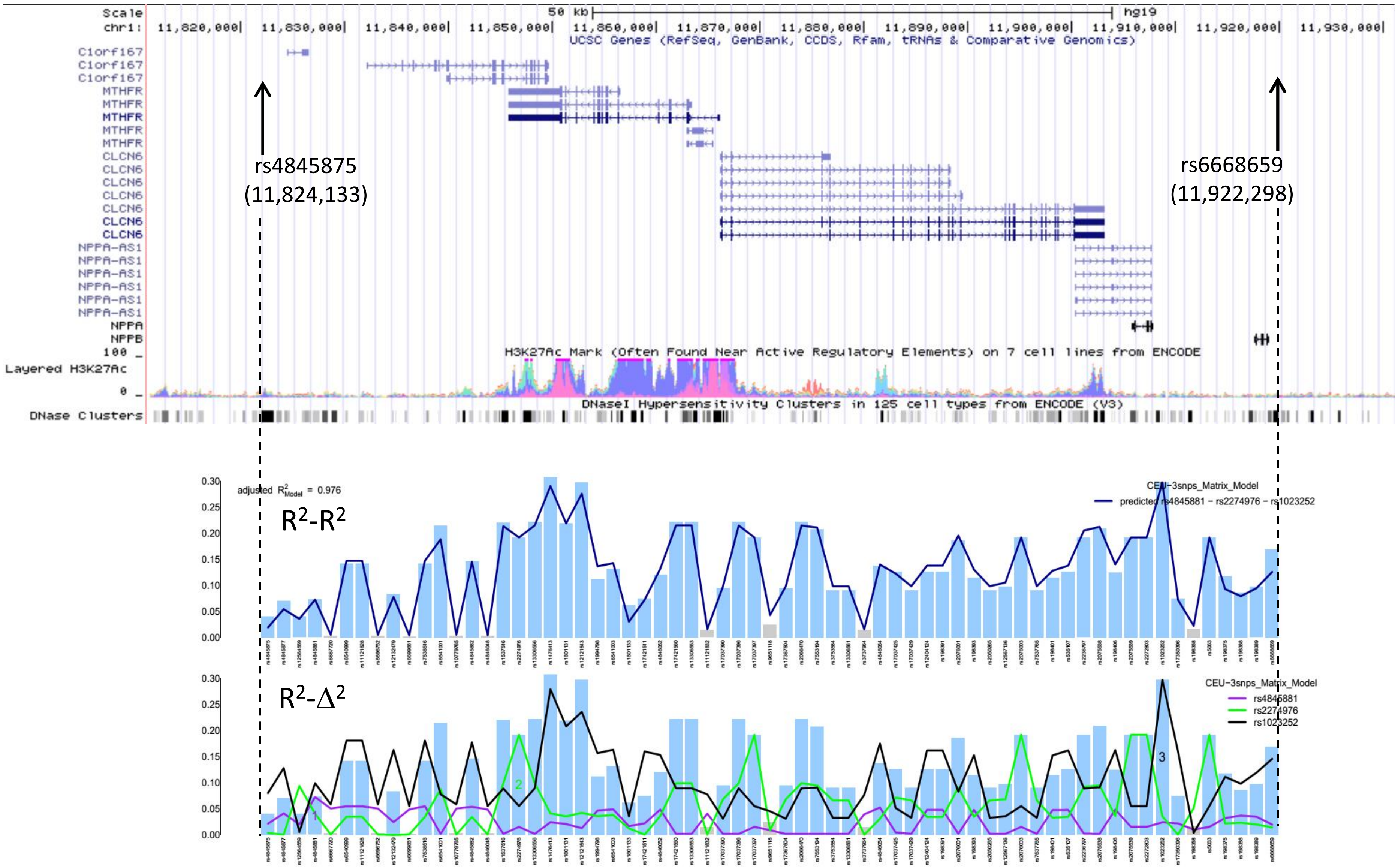

B. JPT-LCLs

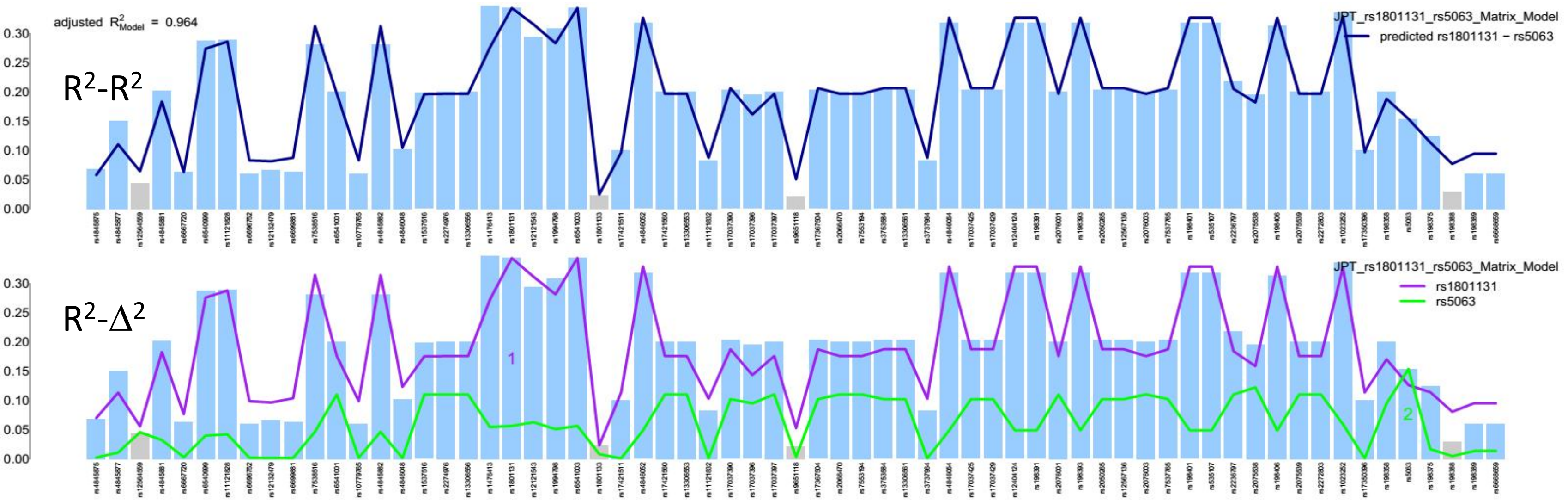

C. CHB-LCLs

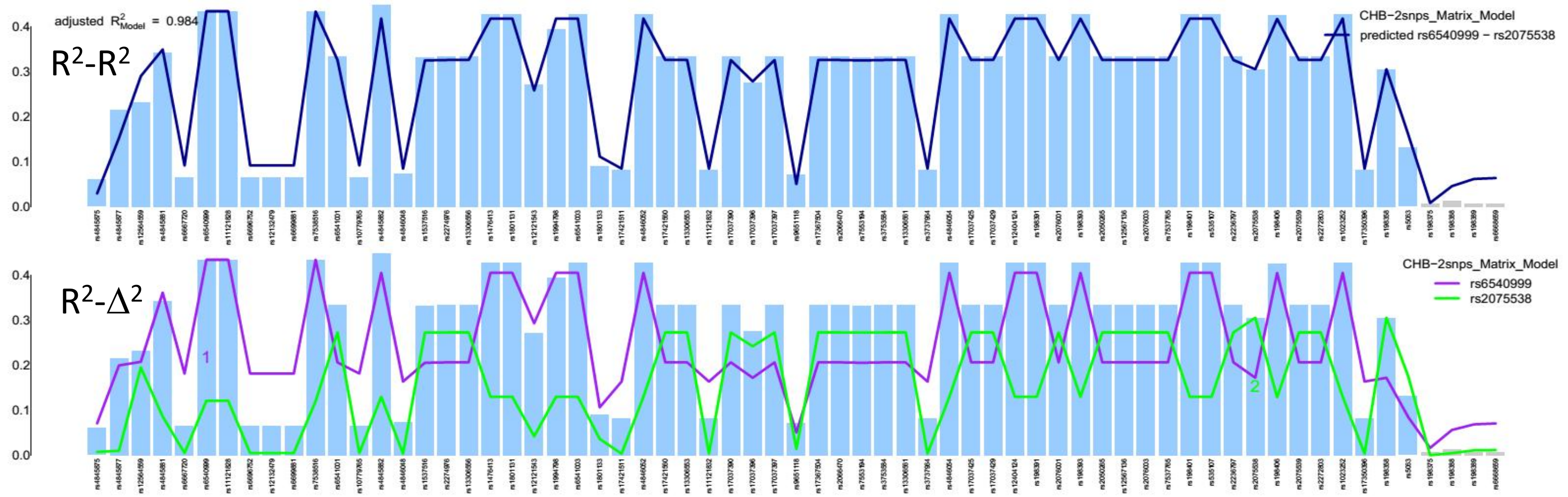

D. CEU-LCLs

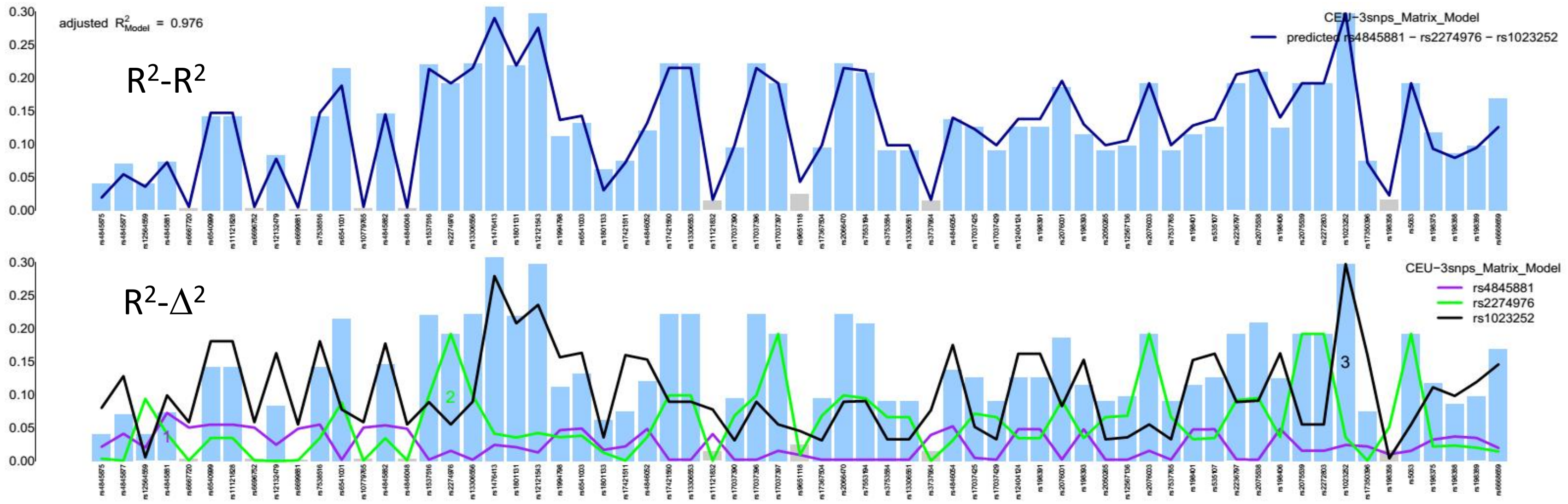

E. Top-3 models

JPT-LCLs

iSNP1	iSNP2	NRMSE	R2.adj	R2.T	R2B	R2C	R2D
rs17421511	rs2236797	7.37942	0.962603	0.352495	0.1001	0.2179	NA
rs1476413	rs1801131	7.385434	0.958166	0.365634	0.3473	0.3439	NA
rs1801131	rs5063	7.439891	0.963879	0.346076	0.3439	0.1543	NA

CHB-LCLs

iSNP1	iSNP2	NRMSE	R2.adj	R2.T	R2B	R2C	R2D
rs6540999	rs2075538	6.61331	0.98432	0.46564	0.4342	0.3055	NA
rs7538516	rs2075538	6.65104	0.98425	0.46555	0.4334	0.3055	NA
rs6540999	rs6541001	6.73541	0.98552	0.46299	0.4342	0.3332	NA

CEU-LCLs

iSNP1	iSNP2	iSNP3	NRMSE	R2.adj	R2.T	R2B	R2C	R2D
rs4845881	rs2274976	rs1023252	6.74137	0.97565	0.36431	0.07291	0.1923	0.2978
rs4845881	rs2274976	rs1476413	7.15789	0.9733	0.36419	0.07291	0.1923	0.3076
rs2274976	rs1330655(	rs1212154:	10.1082	0.93372	0.34311	0.1923	0.2224	0.2984

F. Comparison of iSNP families in JPT-, CHB- and CEU-LCL data sets

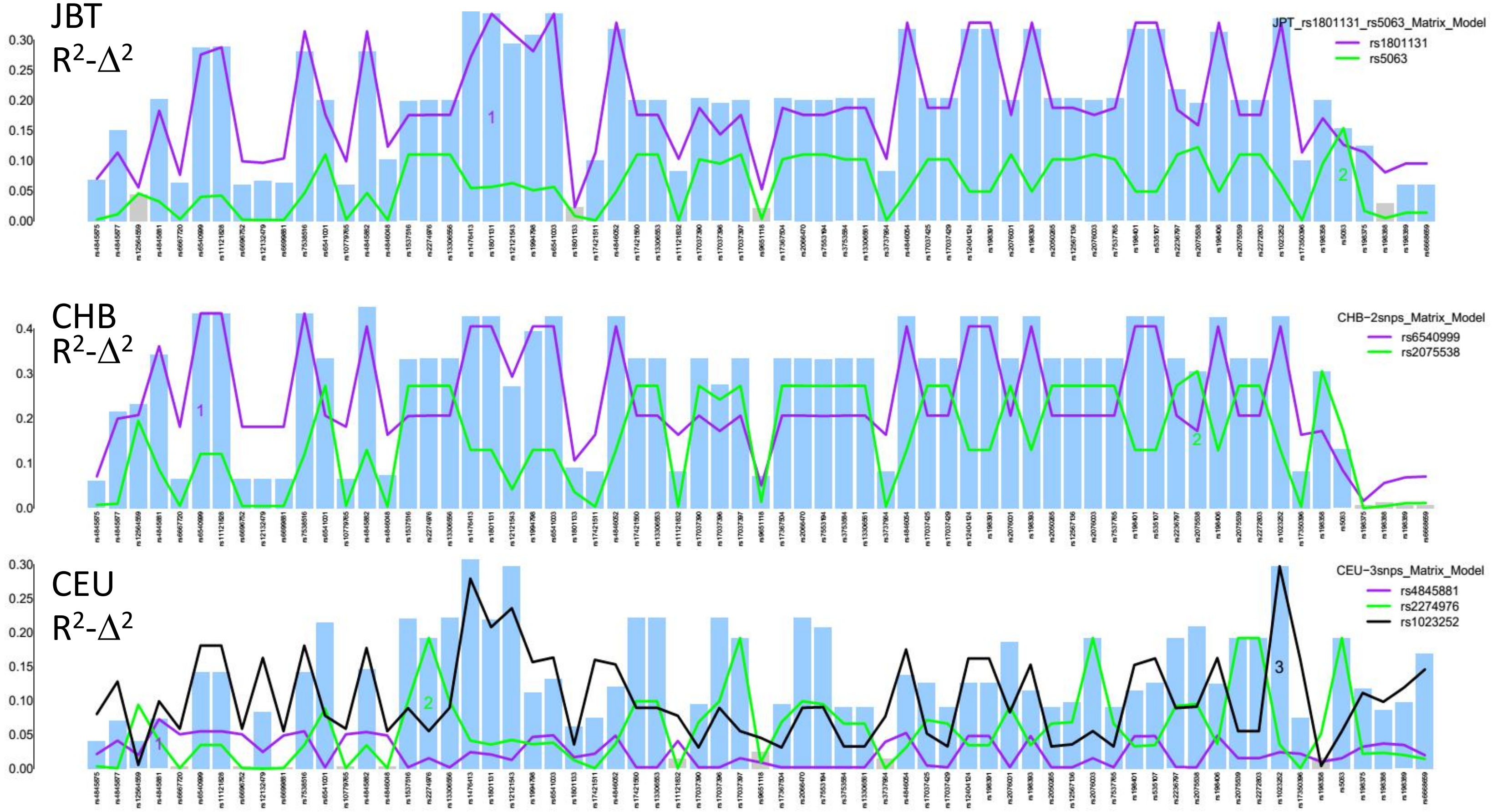

Due to differences in the r^2 LD structure for SNPs in this region of the chromosome, the black and purple iSNP families in the CEU population are contained within single (purple) iSNP families in the JPT and CHB populations. Differences in the relative contributions of the black and purple iSNP families in the CUE population suggest that the "hidden" black iSNP family is also the larger contributor in the JPT and CHB populations.

G. MTHFR-LDlink matrix plots reveal differences in LD structure within the *MTHFR* ROI in Japanese, Chinese and Caucasian populations

JPT

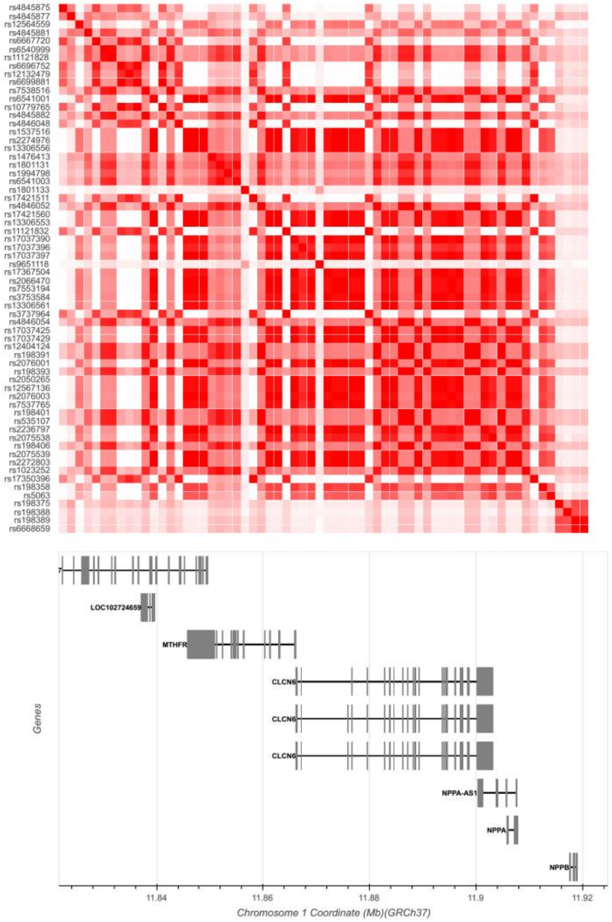

CHB

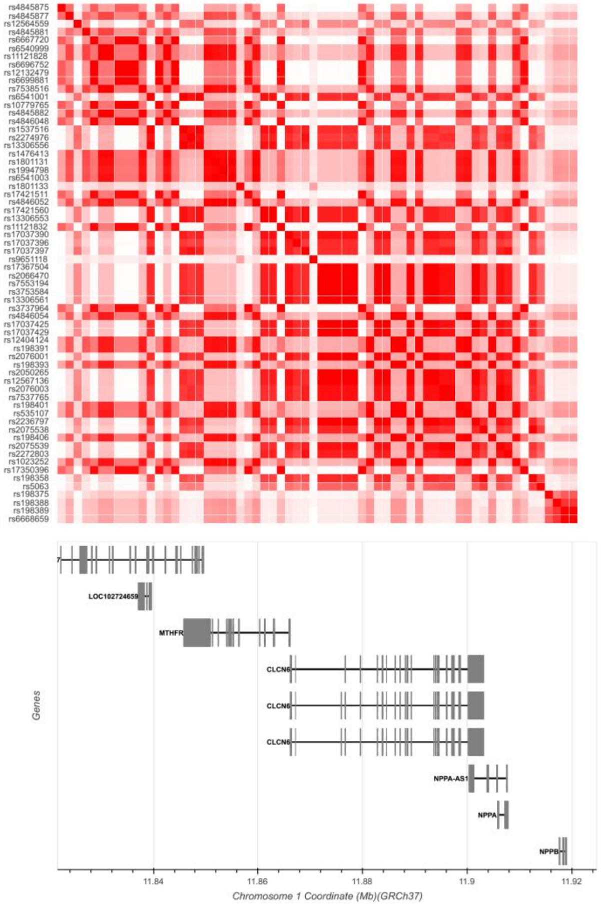

CEU

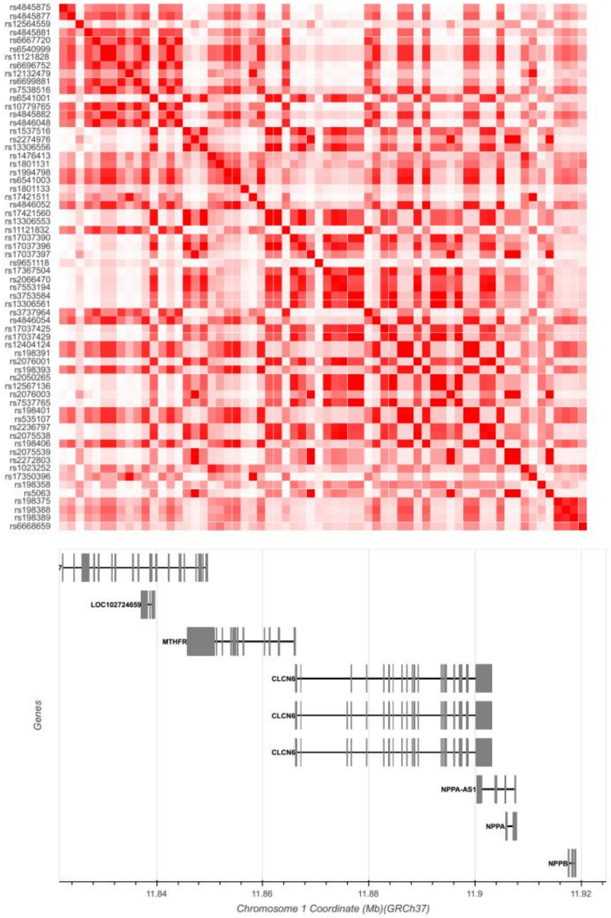

H. iSNP families for JPT, CHB and CEU-LCLs: the third iSNP family in the CEU-LCLs Is contained within the first iSNP families in the JPT- and CHB-LCLs

LCL-JPT	rs1801131	rs5063
	rs1801131	rs2274976
	rs1023252	rs17037397
	rs1476413	rs2076003
	rs12121543	rs2075539
	rs6541003	rs2272803
	rs4846052	rs5063
	rs6668659	rs13306556
	rs1994798	rs17421560
	rs198406	rs13306553
	rs12404124	rs17037396
	rs198391	rs2066470
	rs198393	rs1537516
	rs535107	rs2075538
	rs198401	rs7553194
	rs6540999	rs12564559
	rs11121828	rs2236797
	rs7538516	rs2076001
	rs4845882	rs6541001
	rs4846054	rs17037425
	rs198389	rs12567136
	rs17037390	rs3753584
	rs17367504	rs13306561
	rs198375	rs17037429
	rs12132479	rs2050265
	rs4845877	rs7537765
	rs17421511	rs198358
	rs17350396	rs4845875
	rs4845881	
	rs198388	
	rs9651118	
	rs6699881	
	rs6667720	
	rs6696752	
	rs10779765	
	rs1801133	
	rs4846048	
	rs11121832	
	rs3737964	

LCL-CHB	rs6540999	rs2075538
	rs6540999	rs2236797
	rs11121828	rs7553194
	rs7538516	rs2075538
	rs4845882	rs2076001
	rs4846054	rs13306556
	rs6541003	rs17421560
	rs4846052	rs13306553
	rs198406	rs17037396
	rs12404124	rs2066470
	rs198391	rs1537516
	rs198393	rs6541001
	rs535107	rs17037425
	rs198401	rs12567136
	rs1994798	rs3753584
	rs4845877	rs13306561
	rs4845881	rs17037429
	rs198388*	rs2050265
	rs1023252	rs7537765
	rs1476413	rs17037390
	rs198389	rs17367504
	rs6667720	rs2274976
	rs6696752	rs17037397
	rs10779765	rs2076003
	rs6699881	rs2075539
	rs4846048	rs2272803
	rs198375	rs5063
	rs1801131	rs198358
	rs4845875	rs12564559
	rs11121832	
	rs12121543	
	rs3737964	
	rs6668659	
	rs12132479	
	rs17421511	
	rs17350396	
	rs1801133	
	rs9651118	

LCL-CEU	rs4845881	rs2274976	rs1023252
	rs4845881	rs2274976	rs1023252
	rs6540999	rs17037397	rs1476413
	rs11121828	rs2076003	rs12121543
	rs7538516	rs2075539	rs1801131
	rs4845882	rs2272803	rs12132479
	rs4846054	rs5063	rs17421511
	rs6667720	rs13306556	rs17350396
	rs6696752	rs17421560	rs9651118
	rs10779765	rs13306553	
	rs6541003	rs17037396	
	rs6699881	rs2066470	
	rs4846048	rs1537516	
	rs4846052	rs2075538	
	rs198406	rs7553194	
	rs12404124	rs12564559	
	rs198391	rs2236797	
	rs198393	rs2076001	
	rs535107	rs6541001	
	rs198401	rs17037425	
	rs1994798	rs17037390	
	rs4845877	rs17367504	
	rs11121832	rs12567136	
	rs3737964	rs3753584	
	rs198388	rs13306561	
	rs198389	rs17037429	
	rs198375	rs2050265	
	rs4845875	rs7537765	
	rs6668659	rs198358	
	rs1801133		

I. R^2 values and regulome scores for CEU-LCL iSNP family members (threshold for inclusion: r^2 LD coefficient with respect to iSNP > 0.6)

LCL-CEU-1	rs4845881	RB2	regulome score
	rs6668659	0.1694	6
	rs4845882	0.146	1f (eQTL: MTHFR; monocytes, CEU-LCLs)
	rs6540999	0.1418	6
	rs11121828	0.1418	1f (eQTL: MTHFR; monocytes, CEU-LCLs)
	rs7538516	0.1418	1f (eQTL: MTHFR; CEU-LCLs)
	rs4846054	0.1384	5
	rs6541003	0.1326	1f (eQTL: MTHFR; CEU-LCLs)
	rs12404124	0.1259	6
	rs198391	0.1259	1f (eQTL: MTHFR; monocytes)
	rs535107	0.1259	1f (eQTL: MTHFR; monocytes)
	rs198406	0.1252	1f (eQTL: MTHFR; CEU-LCLs)
	rs4846052	0.1213	1f (eQTL: MTHFR; CEU-LCLs)
	rs198375	0.1176	1f (eQTL: MTOR; CEU-LCLs)
	rs198401	0.1153	6
	rs198393	0.1145	1f (eQTL: MTHFR; CEU-LCLs)
	rs1994798	0.1126	1f (eQTL: MTHFR; CEU-LCLs)
	rs198389	0.09711	1f (eQTL: MTOR; CEU-LCLs)
	rs198388	0.08586	1f (eQTL: MTOR; CEU-LCLs)
	rs4845881	0.07291	4
	rs4845877	0.07044	4
	rs1801133	0.0616	4
	rs4845875	0.04079	3a (PWM: TP73)
	rs3737964	0.01488	4
	rs11121832	0.01417	5
	rs4846048	0.00411	3a (PWM: Gata3/5)
	rs6667720	0.00373	5
	rs6696752	0.00373	5
	rs10779765	0.00373	4
	rs6699881	0.00168	4

rs2274976	RC2	regulome score
rs13306556	0.2224	5
rs17421560	0.2224	5
rs13306553	0.2224	5
rs17037396	0.2224	6
rs2066470	0.2224	4
rs1537516	0.2203	4
rs6541001	0.2146	6
rs2075538	0.2099	1d (eQTL: MTHFR, CLCN6; monocytes; PWM: NRSF)
rs7553194	0.2076	4
rs2274976	0.1923	3a (FOXO3-PWM)
rs17037397	0.1923	4
rs2076003	0.1923	1f (eQTL: MTHFR, CLCN6;monocytes)
rs2075539	0.1923	1f (eQTL: MTHFR, CLCN6;monocytes)
rs2272803	0.1923	2b (MTF-1-footprinting)
rs5063	0.1923	1f (eQTL: MTHFR, CLCN6;monocytes)
rs2236797	0.1915	5
rs2076001	0.1863	6
rs17037425	0.1259	No Data
rs12567136	0.09758	1f (eQTL: MTHFR; monocytes)
rs17037390	0.0953	4
rs17367504	0.0953	4
rs3753584	0.09008	3a (RARA, RARB, RARG, NR2F1, 6-PWM)
rs13306561	0.09008	4
rs17037429	0.09008	6
rs2050265	0.09008	5
rs7537765	0.09008	6
rs12564559	0.03999	5
rs12564560	0.01615	3a

rs1023252	RD2	regulome score
rs1476413	0.3076	1f (eQTL: THFR; CEU-LCLs; CERE)
rs12121543	0.2984	1f (eQTL: MTHFR, CLCN6; CEU-LCLs, monocytes; STAT1-footprinting)
rs1023252	0.2978	1f (eQTL: MTHFR, CLCN6; CEU-LCLs,monocytes, CERE)
rs1801131	0.2193	1f (eQTL: MTHFR, MTOR; CEU-LCLs, monocytes)
rs12132479	0.08369	5
rs17421511	0.07448	4
rs17350396	0.07448	5
rs9651118	0.02536	3a (PWM: GATA3)

	rs4845881	vlookup FCTX fam3 (0.2)		rs2274976	vlookup FCTX fam 2 (0.2)		rs1023252	vlookup FCTX fam 3 (0.2)
	rs4845881	O		rs2274976	O		rs1023252	O
	rs6540999	O		rs17037397	O		rs1476413	O
	rs11121828	O		rs2076003	O		rs12121543	O
	rs7538516	O		rs2075539	O	r2 > 0.6	rs1801131	O
	rs4845882	O		rs2272803	O		rs12132479	O
	rs4846054	O	r2 > 0.6	rs5063	O		rs17421511	O
	rs6667720	O		rs13306556	O		rs17350396	O
	rs6696752	O		rs17421560	O		rs6668659	
	rs10779765	O		rs13306553	O	r2 > 0.4	rs9651118	
	rs6541003	O		rs17037396	O			
	rs6699881	O		rs2066470	O			
	rs4846048	O		rs1537516	O			
	rs4846052	O		rs2075538	O			
	rs198406	O		rs7553194	O			
	rs12404124	O		rs12564559				
	rs198391	O		rs2236797	O			
	rs198393	O		rs2076001	O			
	rs535107	O	r2 > 0.4	rs6541001	O			
	rs198401	O		rs17037425	O			
r2 > 0.6	rs1994798	O		rs17037390	O			
	rs4845877	O		rs17367504	O			
	rs11121832			rs12567136	O			
	rs3737964			rs3753584	O			
	rs198388	O		rs13306561	O			
	rs198389	O		rs17037429	O			
r2 > 0.4	rs198375			rs2050265	O			
	rs4845875	O		rs7537765	O			
	rs1801133	O		rs198358				

rs2066470	vlookup TCTX (0.2)	vlookup TCTX 0.4	vlookup TCTX(0.6)	vlookupCERE (0.4)	vlookup CERE(0.6)	vlookup CEU-LCLs2 (0.2)	vlookup CEU-LCLs2 (0.4)	vlookup CEU-LCLs2 (0.6)
rs17421560	O	O	O	O	O	O	O	
rs3766747	O	O	O	O	O			
rs13306553	O	O	O	O	O	O	O	
rs35464336	O	O	O	O	O			
rs17037396	O	O	O	O	O	O	O	O
rs2066470	O	O	O	O	O	O	O	O
rs3753588	O	O	O	O	O			
rs7553194	O	O	O	O	O	O	O	
rs17367629	O	O	O	O	O			
rs3753582	O	O	O	O	O			

rs12564593	O	O	O	O	O			
rs2076001	O	O	O	O	O	O		
rs2076002	O	O	O	O	O			
rs55994631	O	O	O	O	O			
rs2076004	O	O	O	O	O			
rs41275478	O	O	O	O	O			
rs41275488	O	O	O	O	O			
rs2236797	O	O	O	O	O	O		
rs72640262	O	O	O	O	O			
rs2075538	O	O	O	O	O	O		
rs72640267	O	O	O	O	O			
rs55788159	O	O	O	O	O			
rs114083374	O	O	O	O	O			
rs72638693	O	O	O	O	O			
rs72638696	O	O	O	O	O			
rs113980419	O	O	O	O	O			
rs72638698	O	O	O	O	O			
rs41275456	O	O	O	O	O			
rs72638700	O	O	O	O	O			
rs141567582	O	O	O	O	O			
rs41275458	O	O	O	O	O			
rs6541001	O	O	O	O	O	O		
rs6688187	O	O	O	O	O			

rs12564593	o	o	o	o	o			
rs2076001	o	o	o	o	o	o		
rs2076002	o	o	o	o	o			
rs55994631	o	o	o	o	o			
rs2076004	o	o	o	o	o			
rs41275478	o	o	o	o	o			
rs41275488	o	o	o	o	o			
rs2236797	o	o	o	o	o	o		
rs72640262	o	o	o	o	o			
rs2075538	o	o	o	o	o	o		
rs72640267	o	o	o	o	o			
rs55788159	o	o	o	o	o			
rs114083374	o	o	o	o	o			
rs72638693	o	o	o	o	o			
rs72638696	o	o	o	o	o			
rs113980419	o	o	o	o	o			
rs72638698	o	o	o	o	o			
rs41275456	o	o	o	o	o			
rs72638700	o	o	o	o	o			
rs141567582	o	o	o	o	o			
rs41275458	o	o	o	o	o			
rs6541001	o	o	o	o	o	o		
rs6688187	o	o	o	o	o			
rs3820192	o	o	o	o	o			
rs1537516	o	o	o	o	o	o		
rs1537515	o	o	o	o	o			
rs1537514	o	o	o	o	o			
rs72640221	o	o	o	o	o			
rs13306556	o	o	o	o	o	o		
rs2066462	o	o	o	o	o			
rs72638682	o	o	o	o	o			
rs55728339	o	o	o	o	o			
rs72638683	o	o	o	o	o			
rs72638684	o	o	o	o	o			
rs55929441	o	o	o	o	o			
rs36029635	o	o	o	o	o			
rs55990055	o	o	o	o	o			
rs114951726	o	o	o	o	o			
rs12131667	o	o	o	o	o			
rs17037425	o	o		o		o		
rs12561919	o	o		o				

rs17037388	o	o		o				
rs45553335	o	o		o				
rs17037390	o	o		o		o		
rs17367504	o	o		o		o		
rs3753584	o	o		o		o		
rs13306561	o	o		o		o		
rs17037427	o	o		o				
rs17037429	o	o		o		o		
rs41275472	o	o		o				
rs2050265	o	o		o		o		
rs6699618	o	o		o				
rs6669371	o	o		o				
rs12567119	o	o		o				
rs12567136	o	o		o		o		
rs56153133	o	o		o				
rs7537765	o	o		o		o		
rs2236796	o	o		o				
rs55741089	o	o		o				
rs41275502	o	o		o				
rs61764044	o	o		o				
rs5068	o	o		o				
rs3753586	o							
rs3753585	o							
rs3737965	o							
rs75747410	o							
rs2076003	o					o		
rs77072136	o							
rs41275484	o							
rs79811212	o							
rs41275500	o							
rs2075539	o					o		
rs2272803	o					o		
rs7552330	o							
rs141308438	o							
rs5063	o					o		
rs17037397	o			o		o		
rs145488887	o	o		o				
rs114941496	o	o		o				
rs3737967	o			o				
rs2274976	o			o		o		
rs61757273	o							
rs112521149								

SNP	SNP	0.2 < TCTX rs4845876	0.6 < TCTX rs4845876	0.6 < CERE rs59911871	0.6 < PONS1 rs12135232	0.6 < PONS2 rs3737966	0.6 < PONS3 rs6662190	CEU-LCLs rs4845881 (0.4)	CEU-LCLs rs1023252 (0.4)
rs4845876	rs4845876	O	O						
rs4845877	rs4845877	O	O				O	O	
rs4845878	rs4845878	O	O				O		
rs4845879	rs4845879	O	O				O		
rs10864536	rs10864536	O	O	O					
rs11121825	rs11121825	O	O				O		
rs11121826	rs11121826	O	O				O		
rs59911871	rs59911871	O		O	O				
rs11121827	rs11121827	O				O			
rs61773952	rs61773952	O		O	O				
rs10779764	rs10779764	O				O			
rs61773953	rs61773953	O		O	O				
rs11809568	rs11809568	O				O			
rs6540999	rs6540999	O				O		O	
rs6541000	rs6541000	O				O			
rs12079693	rs12079693	O				O			
rs12076233	rs12076233	O		O	O				
rs10864538	rs10864538	O				O			
rs10864539	rs10864539	O				O			
rs12735869	rs12735869	O				O			
rs12734761	rs12734761	O				O			
rs11121828	rs11121828	O				O		O	
rs6659287	rs6659287	O				O			
rs6659541	rs6659541	O				O			
rs12132479	rs12132479	O		O	O				O
rs7538516	rs7538516	O				O		O	
rs12134663	rs12134663	O		O	O				
rs6697244	rs6697244	O				O			
rs4846043	rs4846043	O				O			
rs41275462	rs41275462	O		O	O				
rs4846045	rs4846045	O				O			
rs1889292	rs1889292	O				O			
rs3737970	rs3737970	O				O			
rs2001584	rs2001584	O				O			
rs2004445	rs2004445	O				O			
rs12119092	rs12119092	O		O	O				
rs12141216	rs12141216	O		O	O				
rs9727993	rs9727993	O	O	O		O			
rs10864542	rs10864542	O				O			
rs61776071	rs61776071	O		O	O				
rs4845882	rs4845882	O				O		O	
rs12135232	rs12135232	O		O	O				
rs10864543	rs10864543	O				O			
rs59375726	rs59375726	O		O	O				
rs11559040	rs11559040	O		O	O				
rs4846049	rs4846049	O							
rs1476413	rs1476413	O							O
rs1801131	rs1801131	O							O
rs17421511	rs17421511	O		O	O				O
rs45449597	rs45449597	O		O	O				
rs45608437	rs45608437	O		O	O				
rs45504202	rs45504202	O		O	O				
rs2066471	rs2066471	O		O	O				
rs3753587	rs3753587	O		O	O				
rs4846054	rs4846054	O				O		O	
rs12123964	rs12123964	O		O	O				
rs35983202	rs35983202	O		O	O				
rs1023252	rs1023252	O							O
rs17350396	rs17350396	O		O	O				O
rs34710782	rs34710782	O		O					

	SNP	SNP	0.2 < TCTX rs4845876	0.6 < TCTX rs4845876	0.6 < CERE rs59911871	0.6 < PONS1 rs12135232	0.6 < PONS2 rs3737966	0.6 < PONS3 rs6662190	CEU-LCLs rs4845881 (0.4)	CEU-LCLs rs1023252 (0.4)
r2 = 0.439	rs4845875	rs4845875	O						O	
	rs6677253	rs6677253	O					O		
	rs6674634	rs6674634	O					O		
	rs6662190	rs6662190	O					O		
	rs6677240	rs6677240	O					O		
	rs10218813	rs10218813	O					O		
	rs6668699	rs6668699	O					O		
	rs6540997	rs6540997	O					O		
	rs4846037	rs4846037	O					O		
	rs4845880	rs4845880	O					O		
	rs4845881	rs4845881	O					O	O	
	rs6667720	rs6667720	O					O	O	
	rs6680385	rs6680385	O					O		
	rs11804825	rs11804825	O					O		
	rs11805138	rs11805138	O					O		
	rs6689912	rs6689912	O					O		
	rs11121829	rs11121829	O					O		
	rs9792894	rs9792894	O					O		
	rs6696752	rs6696752	O					O	O	
	rs6699881	rs6699881	O					O	O	
	rs4846039	rs4846039	O					O		
	rs4846040	rs4846040	O					O		
	rs4846041	rs4846041	O					O		
	rs10779765	rs10779765	O					O	O	
	rs10864540	rs10864540	O					O		
	rs10864541	rs10864541	O					O		
	rs1889293	rs1889293	O					O		
	rs4846047	rs4846047	O					O		
	rs2151655	rs2151655	O					O		
	rs4846048	rs4846048	O					O	O	
	rs3737966	rs3737966	O				O			
	rs1994798	rs1994798	O				O		O	
	rs6541003	rs6541003	O				O		O	
	rs7526128	rs7526128	O				O			
	rs6541005	rs6541005	O				O			
	rs4846052	rs4846052	O				O		O	
	rs6658684	rs6658684	O				O			
	rs12406667	rs12406667	O				O			
	rs12404124	rs12404124	O				O		O	
	rs198391	rs198391	O				O		O	
	rs198392	rs198392	O				O			
	rs198393	rs198393	O				O		O	
	rs198397	rs198397	O				O			
	rs198399	rs198399	O				O			
	rs198401	rs198401	O				O		O	
	rs535107	rs535107	O				O		O	
	rs503040	rs503040	O				O			
	rs198406	rs198406	O				O		O	
	rs198408	rs198408	O				O			
	rs198369	rs198369	O				O			
	rs6668352	rs6668352	O							
	rs198379	rs198379	O							
	rs549596	rs549596								
	rs198388	rs198388	O						O	
	rs198389	rs198389	O						O	
	rs3753581	rs3753581	O							
	rs12406089	rs12406089	O							
	rs12402363	rs12402363	O							
r2 > 0.2	rs12406383	rs12406383	O							

Supplementary File 8. Tables S1 and S2

Table S1. Summary of *MTHFR* iSNP families identified in 4BrainR and LCL data sets

Data set	FCTX iSNP family 1 (red)	FCTX iSNP family2 (green)	FCTX iSNP family 3 (black)	LCL iSNP family 4 (purple)
4BrainR-450-FCTX	O	O	O	
4BrainR-450-TCTX	O	O	O	
4BrainR-450-CERE	O	O	O	
4BrainR-450-PONS			O	
4BrainR-150-FCTX	O	O	O	
4BrainR-150-TCTX	O	O	O	
4BrainR-150-CERE	O	O	O	
4BrainR-150-PONS			O	
LCLs-65-JPT		O	O	O
LCLs-65-CHB		O	O	O
LCLs-65-CEU		O	O	O

*Note: each iSNP in the PONS 3-iSNP model is a member of FCTX iSNP family 3

Table S2. SNP ID numbers and properties of *MTHFR* iSNPs identified in in 4BrainR and LCL data sets

Data set	Model number[1]	iSNPs in selected model (SNP_B, SNP_C, SNP_D)[2]	NRMSE (model)	Adjusted R^2 (model)	Total R^2 (model)	$R^2(SNP_B)$	$R^2(SNP_C)$	$R^2(SNP_D)$
4BrainR-450								
FCTX	1	rs198368, rs2066470, rs10864536	9.478352	0.973063	0.226887	0.1209	0.1006	0.06841
TCTX	1	rs78329225, rs114951726, rs4845876	8.126258	0.960135	0.304054	0.1625	0.07001	0.1641
CERE	1	rs198402, rs72638693, rs59911871	5.288372	0.950737	0.248605	0.0893	0.09388	0.06102
PONS	1	rs6662190, rs3737966, rs12135232	6.327761	0.925576	0.24273	0.03244	0.09138	0.1925

4BrainR-139								
FCTX	1	rs198368, rs2066470, rs10864536	16.103681	0.935674	0.226887	0.1209	0.1006	0.06841
TCTX	1	rs55990971, rs145488887,rs6659541	12.230959	0.954846	0.310831	0.1731	0.07597	0.03908
CERE	1	rs198402, rs9727993, rs17376426	10.621742	0.910688	0.283969	0.0893	0.1613	0.08171
PONS	1	rs6662190, rs3737966, rs12135232	7.844431	0.943187	0.24273	0.03244	0.09138	0.1925
LCL-65[3]								
JPT	3	rs1801131, rs5063	7.439891	0.963879	0.346076	0.3439	0.1543	-
CHB	1	rs6540999, rs2075538	6.613307	0.984323	0.465636	0.4342	**0.3055**	-
CEU	1	rs4845881, rs2274976, rs1023252	6.741374	0.975647	0.364305	0.07291	0.1923	0.2978

[1]Models are ordered in increasing size of NRMSE. The JPT-LCL #3 model was chosen to better illustrate the conservation of the "green" iSNP family in the R^2-Δ^2 plot.

[2]SNP rs numbers are color-coded according to membership in FCTX iSNP family 1(red), iSNP family 2 (green) and iSNP family 3 (black).

[3]The "purple" family SNPs in CEU-LCLs comprises primarily FCTX "black" family SNPs; iv) the "purple" family SNPs in JPT- and CHB-LCLs also FCTX "black" family SNPs, including both the purple and black family SNPs the identified in CEU-LCLs.

Supplementary File 9. Expression of *CHI3L2* mRNA in CEU-LCLs and 4BrainR-TCTX data sets

A.

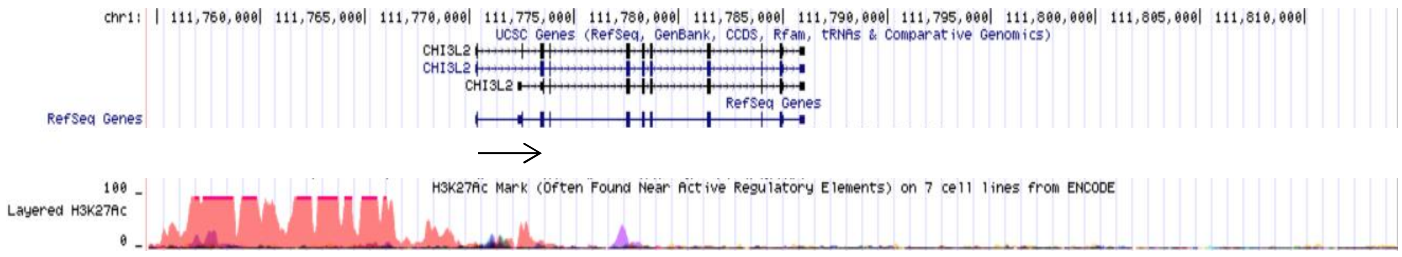

B.

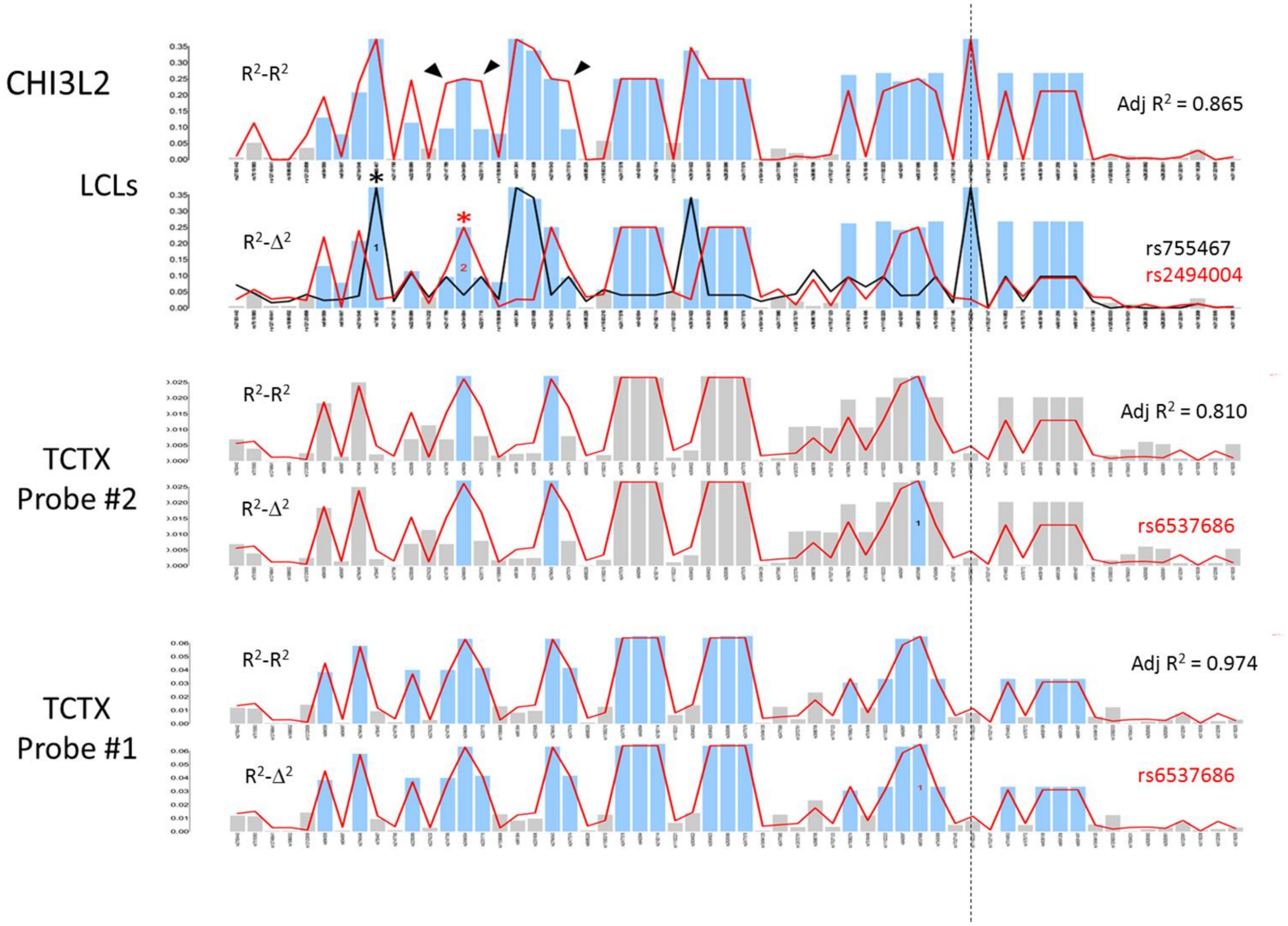

C

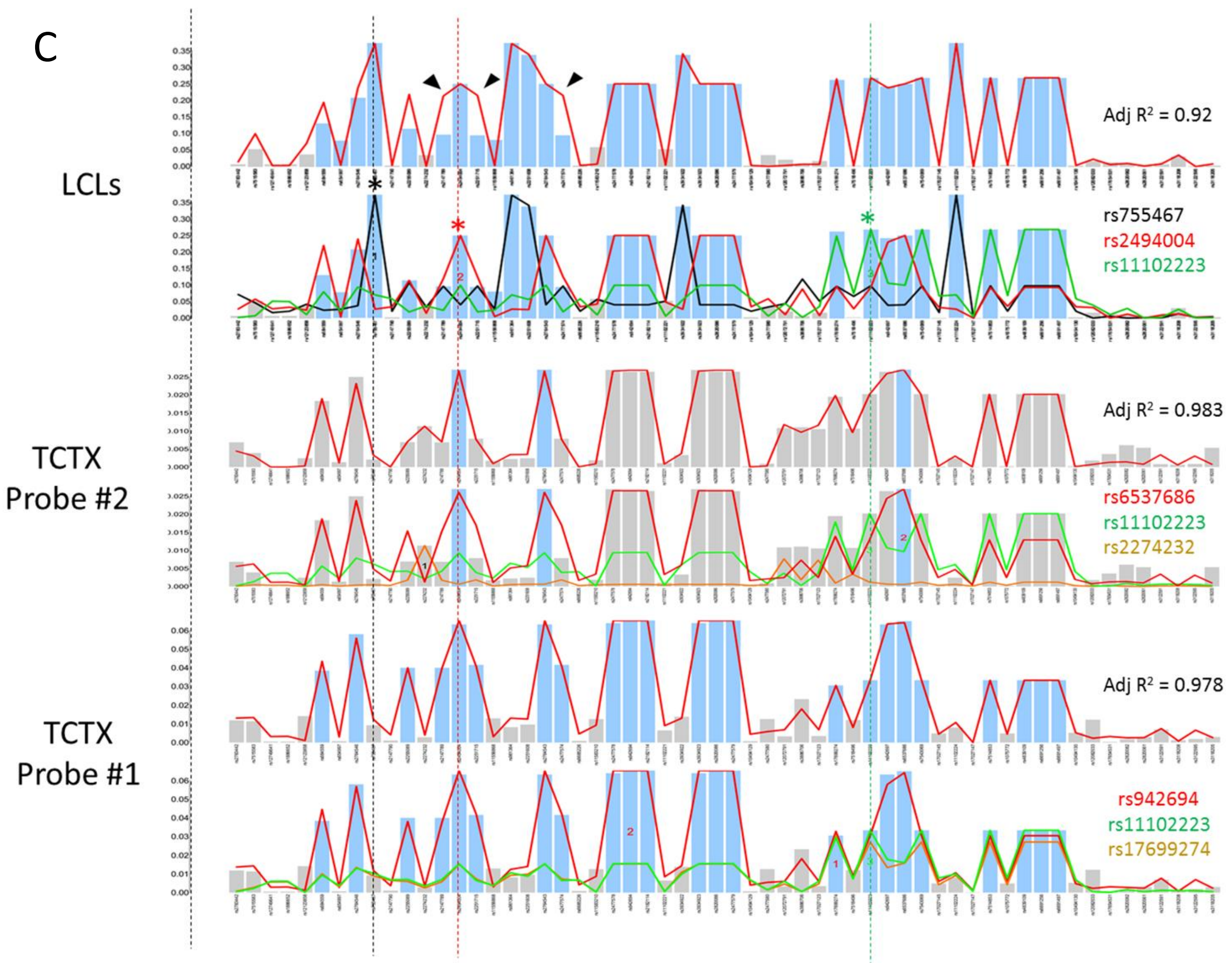
LCLs
Adj R² = 0.92
rs755467
rs2494004
rs11102223
TCTX
Probe #2
Adj R² = 0.983
rs6537686
rs11102223
rs2274232
TCTX
Probe #1
Adj R² = 0.978
rs942694
rs11102223
rs17699274

D

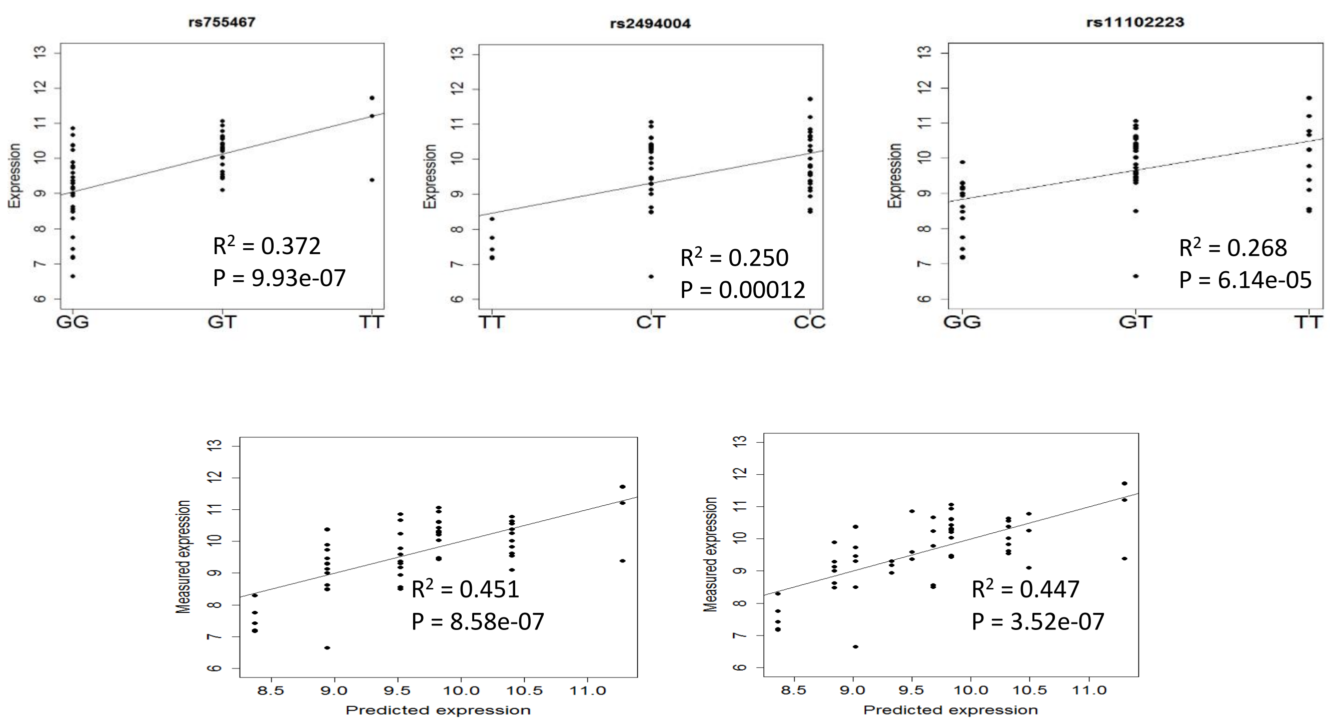
rs755467
Expression
R² = 0.372
P = 9.93e-07
GG
GT
TT
rs2494004
Expression
R² = 0.250
P = 0.00012
TT
CT
CC
rs11102223
Expression
R² = 0.268
P = 6.14e-05
GG
GT
TT
Measured expression
R² = 0.451
P = 8.58e-07
Predicted expression
Measured expression
R² = 0.447
P = 3.52e-07
Predicted expression

Legend

A. Screenshot from the Santa Cruz Genome Browser (Chr1:111,754,616 – 111,814,475; GRCh37/hg19) showing the positions and exon/intron structures of *CHI3L2* (*Chitinase 3 like 2*) and locations of histone III lysine 27 acetylation (H3K27Ac) detected in multiple human cell lines, including LCLs [red: GM12878, a B-lymphocyte-derived lymphoblastoid cell line (lcl)]. Arrow indicates the direction of transcription (5'-to-3'). **B and C**. Upper R^2-R^2 plots show correlations between "measured" (grey and blue bars) and calculated (red lines) values of R^2_A for 58 SNPs within the *CHI3L2* locus for two-index SNP (**B**) and three-index SNP (**C**) models. Lower R^2-Δ^2 plots show values of Δ^2 for SNPs within this locus with respect to two (**B**) or three (**C**) index SNPs, scaled to the heights of the blue bars of the index SNPs. Color-coded asterisks (*) identify index SNPs (1) rs755467 (black), (2) rs2494004 (red) and (3) rs11102223 (green) and lines of the same colors plot the value of Δ^2 for the SNPs in this gene region with respect to the individual index SNPs. Arrowheads identify SNPs for which the two- and three-SNP models yielded less than optimal fits to the experimental data.

Results

To validate our results obtained with simulated mRNA expression/genotype datasets, we used our matrix equation method to analyze of *CHI3L2* mRNA expression in lymphoblastoid cell lines. We chose this gene because it had been analyzed and discussed in several published studies [1][2][3][4][5], and the presence of at least regulatory variant in the promoter region had been experimentally verified. As described in Online Methods, mRNA expression and genotype data for 54 independent LCLs of Caucasian origin were downloaded from the Gene Expression Omnibus (GEO) and dbGAP, respectively. After removal of low frequency SNPs (MAF < 0.01), *58* SNPs within the *CHI3L2* locus at Chr1p13.3 (A) were retained for analysis. As shown in panel B, screening for the best two-iSNP model identified rs755467 and rs2494004 as the top choices (adjusted R^2_{model}= 0.8646). rs755467 has previously been identified as a *CHI3L2* eQTL and possible regulatory variant [1].

Screening for the best three-iSNPs model identified rs755467, rs2494004 and rs11102223 as the top choices. The three-iSNP model was slightly improved compared to the two-iSNP model, with an adjusted R_{Model}^2 = 0.9210). As shown in panel D, linear regression analysis of mRNA expression vs SNP genotypes revealed that rs755467, rs2494004 and rs11102223 individually account for 37.2%, 25% and 26.8% of the variance in *CHI3L2* mRNA expression, respectively, while rs755467 and rs2494004 together account for 45.1% and rs755467, rs2494004 and rs11102223 together 44.7% of the variance of *CHI2L2* mRNA expression.

References

[1] V.G. Cheung, R.S. Spielman, K.G. Ewens, T.M. Weber, M. Morley, J.T. Burdick, Mapping determinants of human gene expression by regional and genome-wide association, Nature. (2005). doi:10.1038/nature04244.

[2] J. Wessel, N.J. Schork, Generalized Genomic Distance–Based Regression Methodology for Multilocus Association Analysis, Am. J. Hum. Genet. (2006). doi:10.1086/508346.

[3] G.K. Chen, J.S. Witte, Enriching the Analysis of Genomewide Association Studies with Hierarchical Modeling, Am. J. Hum. Genet. (2007). doi:10.1086/519794.

[4] N. Malo, O. Libiger, N.J. Schork, Accommodating Linkage Disequilibrium in Genetic-Association Analyses via Ridge Regression, Am. J. Hum. Genet. (2008). doi:10.1016/j.ajhg.2007.10.012.

[5] F. Hormozdiari, E. Kostem, E.Y. Kang, B. Pasaniuc, E. Eskin, Identifying causal variants at loci with multiple signals of association, Genetics. (2014). doi:10.1534/genetics.114.167908.

Supplementary File 10.
DGCR8 mRNA expression in 4BrainR and BC data sets

A

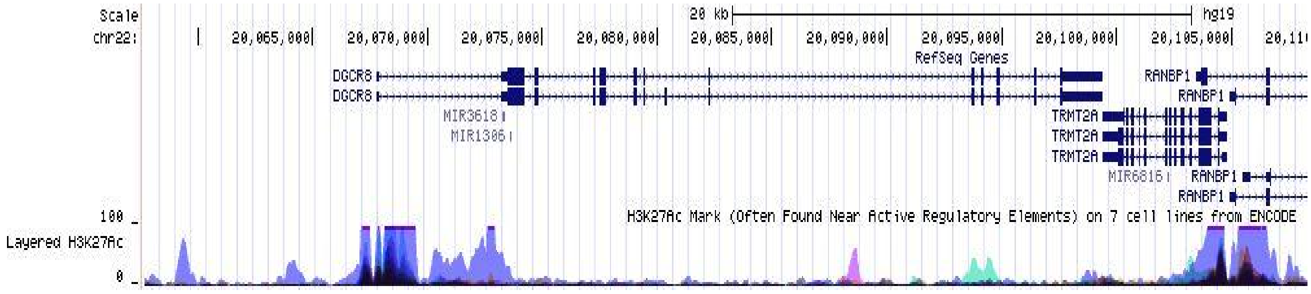

Scale
chr22:
20 kb
hg19
20,065,000
20,070,000
20,075,000
20,080,000
20,085,000
20,090,000
20,095,000
20,100,000
20,105,000
RefSeq Genes
DGCR8
DGCR8
MIR3618
MIR1306
RANBP1
RANBP1
TRMT2A
TRMT2A
TRMT2A
MIR6816
RANBP1
RANBP1
100
Layered H3K27Ac
0
H3K27Ac Mark (Often Found Near Active Regulatory Elements) on 7 cell lines from ENCODE

B

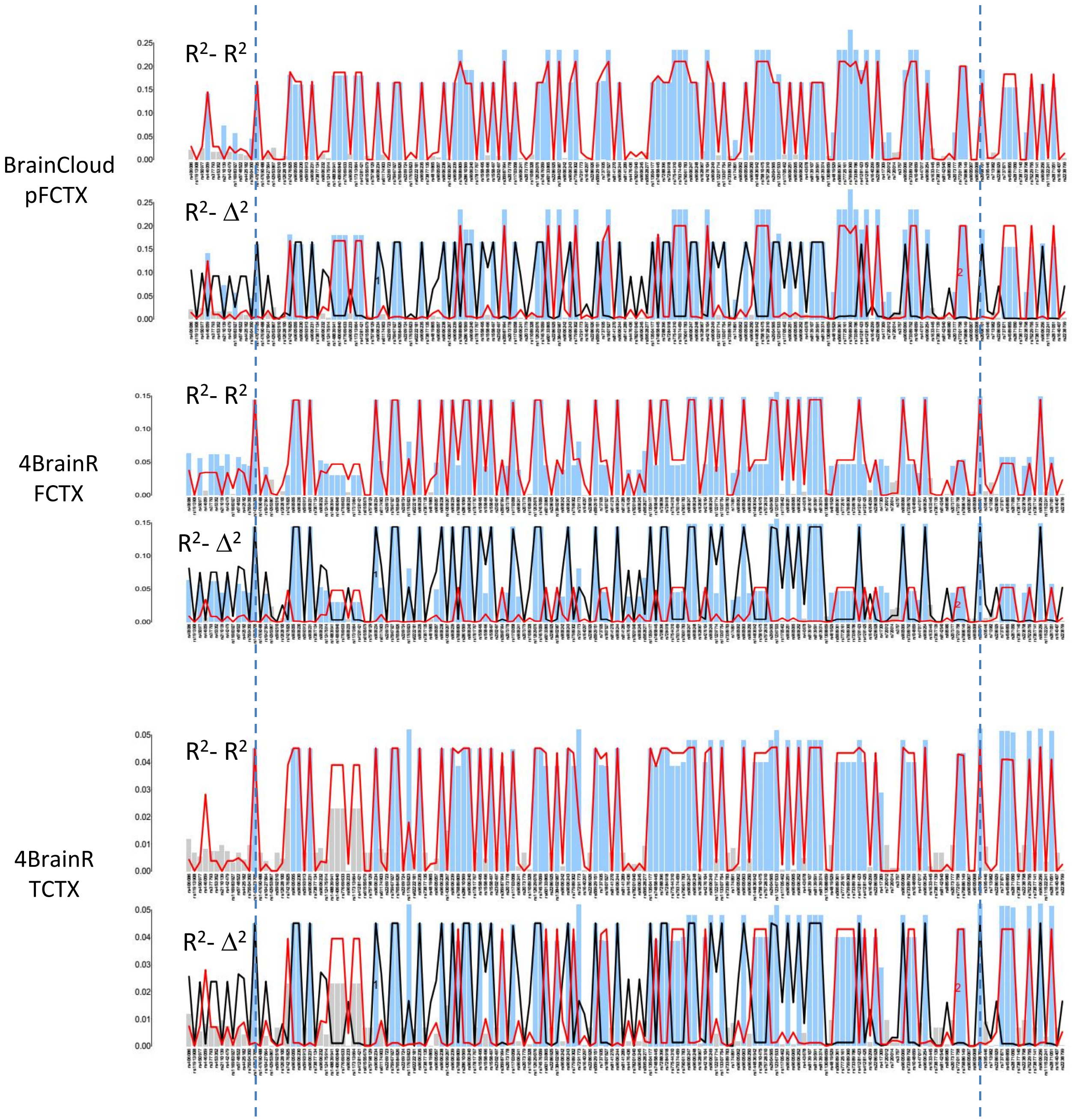

BrainCloud
pFCTX
R2- R2
R2- Δ2
4BrainR
FCTX
R2- R2
R2- Δ2
4BrainR
TCTX
R2- R2
R2- Δ2

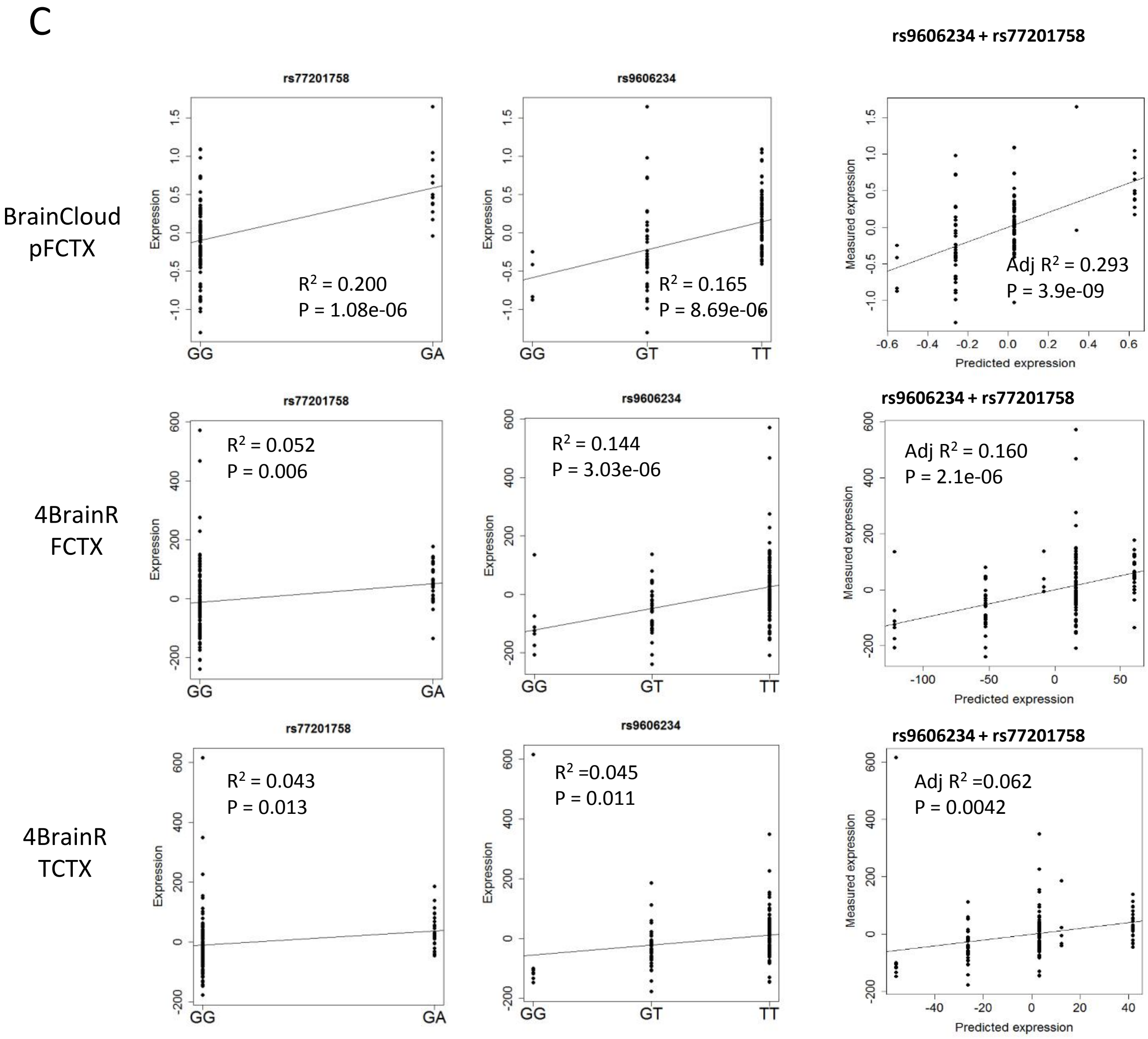
C
BrainCloud pFCTX
4BrainR FCTX
4BrainR TCTX
rs77201758
rs9606234
rs9606234 + rs77201758
Expression
Measured expression
Predicted expression
GG
GA
GT
TT
R² = 0.200
P = 1.08e-06
R² = 0.165
P = 8.69e-06
Adj R² = 0.293
P = 3.9e-09
R² = 0.052
P = 0.006
R² = 0.144
P = 3.03e-06
Adj R² = 0.160
P = 2.1e-06
R² = 0.043
P = 0.013
R² =0.045
P = 0.011
Adj R² =0.062
P = 0.0042

Legend

A.

Two-iSNP models for DGCR8 mRNA expression in human brain. Matrix equation-based analysis of measured coefficients of determination (R^2) values obtained from linear regression analysis of *DGCR8* mRNA expression in human brain vs genotypes of SNPs within the *DGRC8* (*DiGeorge Critical Region-8*) locus at chromosome 22q11.2. (mRNA and genotype data from the BrainCloud (BC) [1] and 4BrainR [2] studies.)

B. Two-iSNP models for *DBCR8* mRNA expression in human pre-frontal cortex (pFCTX), frontal cortex (FCTX) and temporal cortex (TCTX) based on index SNPS (iSNPs) identified by the matrix equation analysis of coefficient of determination (R^2) values for SNPs in the region of the *DGCR8* gene at chromosome 22q11.2. The "R^2-R^2" graph within each pair of graphs are plots of "measured" R^2 values (blue bars) vs "predicted" R^2 values (red line). The "R^2-Δ^2" graph (aka SNP "family plot") within each pair are plots of "measured" R^2 (blue bars) values vs. Δ^2 values calculated for each SNP with respect to each of two iSNPs (red and black lines), scaled to the height of the measured R^2 value for the corresponding iSNP. Dashed vertical lines identify representative "black family" SNPs that replicate among the three datasets. Red family iSNP = rs77201758; black family iSNP = rs9606234. Measures of goodness of fit for two-SNP models shown in R^2-R^2 plots): R^2_{model} = 0.9555; P < 2.2e-16 (BrainCloud pFCTX); R^2_{model} = 0.9210; P < 2.2e-16 (4BainR FCTX); R^2_{model} = 0.8876; P < 2.2e-16 (4BrainR TCTX).

C. Linear regression analysis of DGCR8 mRNA expression versus genotype for iSNPs rs9606234 and rs77201758 analyzed separately (left and middle), and together (right). Adj R^2 = adjusted R^2

Linear regression analysis of DGCR8 mRNA expression in the BrainCLoud and 4BrainR data sets versus genotypes for iSNPs identified by the matrix-based coefficient of multiple determination method.

References

[1] C. Colantuoni, B.K. Lipska, T. Ye, T.M. Hyde, R. Tao, J.T. Leek, E.A. Colantuoni, A.G. Elkahloun, M.M. Herman, D.R. Weinberger, J.E. Kleinman, Temporal dynamics and genetic control of transcription in the human prefrontal cortex, Nature. (2011). doi:10.1038/nature10524.

[2] J.R. Gibbs, M.P. van der Brug, D.G. Hernandez, B.J. Traynor, M.A. Nalls, S.L. Lai, S. Arepalli, A. Dillman, I.P. Rafferty, J. Troncoso, R. Johnson, H.R. Zielke, L. Ferrucci, D.L. Longo, M.R. Cookson, A.B. Singleton, Abundant quantitative trait loci exist for DNA methylation and gene expression in Human Brain, PLoS Genet. (2010). doi:10.1371/journal.pgen.1000952.

Supplementary File 11.
GSTM3-GSTM5 mRNA expression in 4BrainR and BC data sets

1. Two-iSNP models derived from matrix equation-based analysis of measured coefficients of determination (R^2) values obtained from linear regression analysis of *GSTM3* and *GSTM5* (*Glutathione S-transferase mu-1/5*) mRNA expression in human brain. (Data from BrainCloud [1] and 4BrainR [2] databases.)

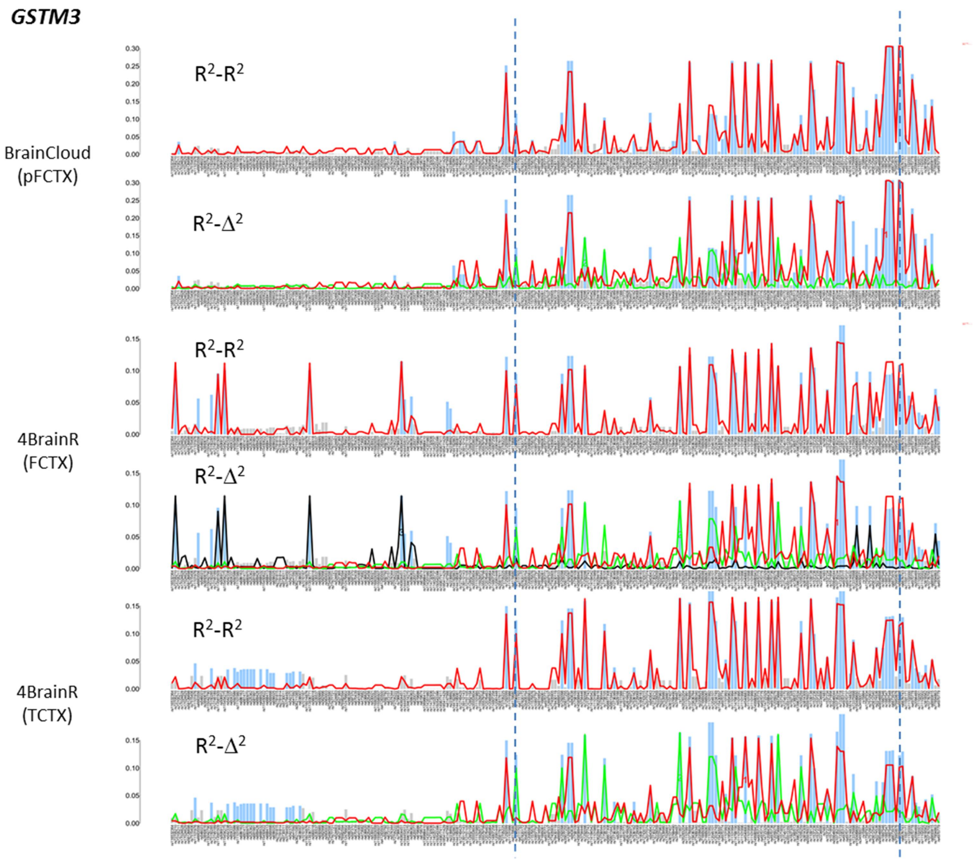

Figure S10.1 Two-iSNP models for *GSTM3* mRNA expression in human pre-frontal cortex (pFCTX), frontal cortex (FCTX) and temporal cortex (TCTX) based on index SNPS (iSNPs) identified by matrix equation analyis of coefficient of determination (R^2) values for SNPs in the region of the *GSTM3* and *GSTM5* genes at chromosome 1. The"R^2-R^2" graphs are plots of "measured" R^2 values (blue and grey bars) versus calculated R^2 values (red line). The "R^2-Δ^2" graphs (aka iSNP "family plot") are plots of "measured" R^2 values (blue bars and grey) vs Δ^2 values calculated for each SNP with respect to each of two iSNPs (red and black lines), scaled to the height of the measured R^2 value for the corresponding iSNP. Dashed vertical lines identify representative "red" and "green" SNPs that replicate among the three datasets.

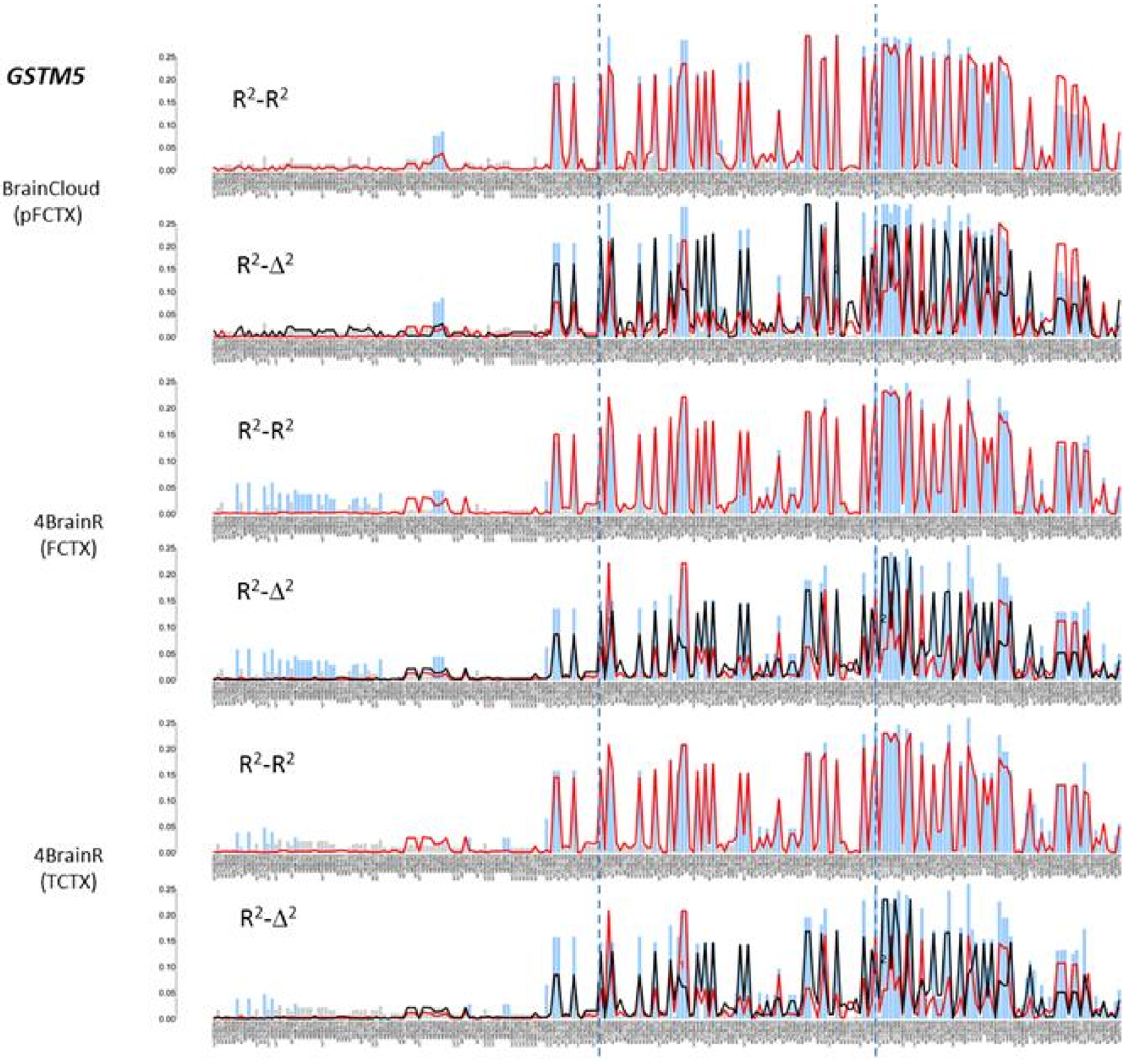

Figure S10.2 Two-iSNP models for *GSTM5* mRNA expression in pFCTX, FCTX, and TCTX data sets based on index SNPS (iSNPs) identified by the matrix equation analyis of coefficient of determination (R^2) values for SNPs in the region of the *GSTM3* gene at chromosome 1. Dashed vertical lines identify representative "black" and "red" family SNPs that replicate among the three datasets.

3. Comparisons of *GSTM3* and *GSTM5* iSNP families in pFCTX, FCTX, TCTX, CERE and PONS data sets.

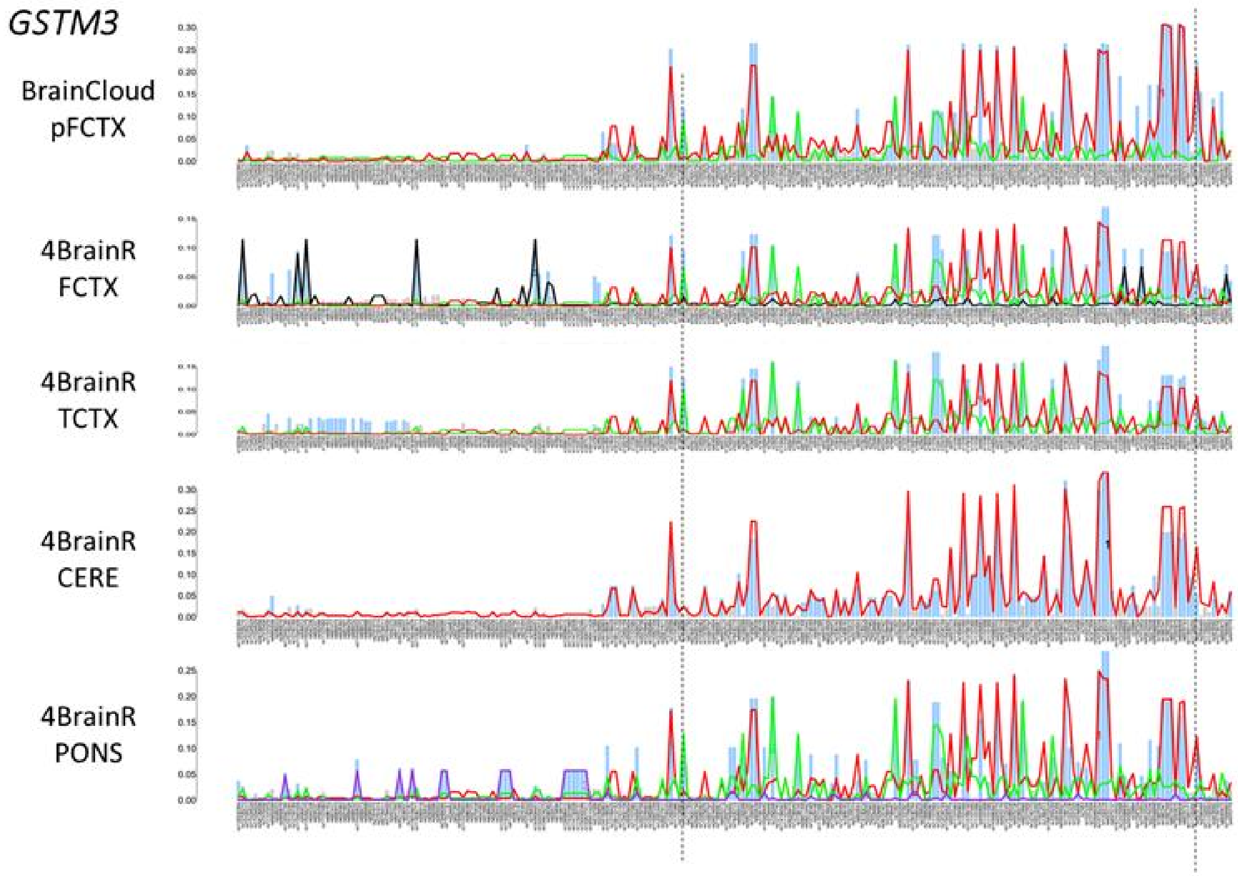

Figure S10.3 Alignment of R^2-Δ^2 plots reveals conservation of "red" family and "green" family SNPs in pFCTX, FCTX, TCTX, and PONS data sets. By contrast, only the red SNP family is observed in mRNA isolated from the cerebellum (CERE).

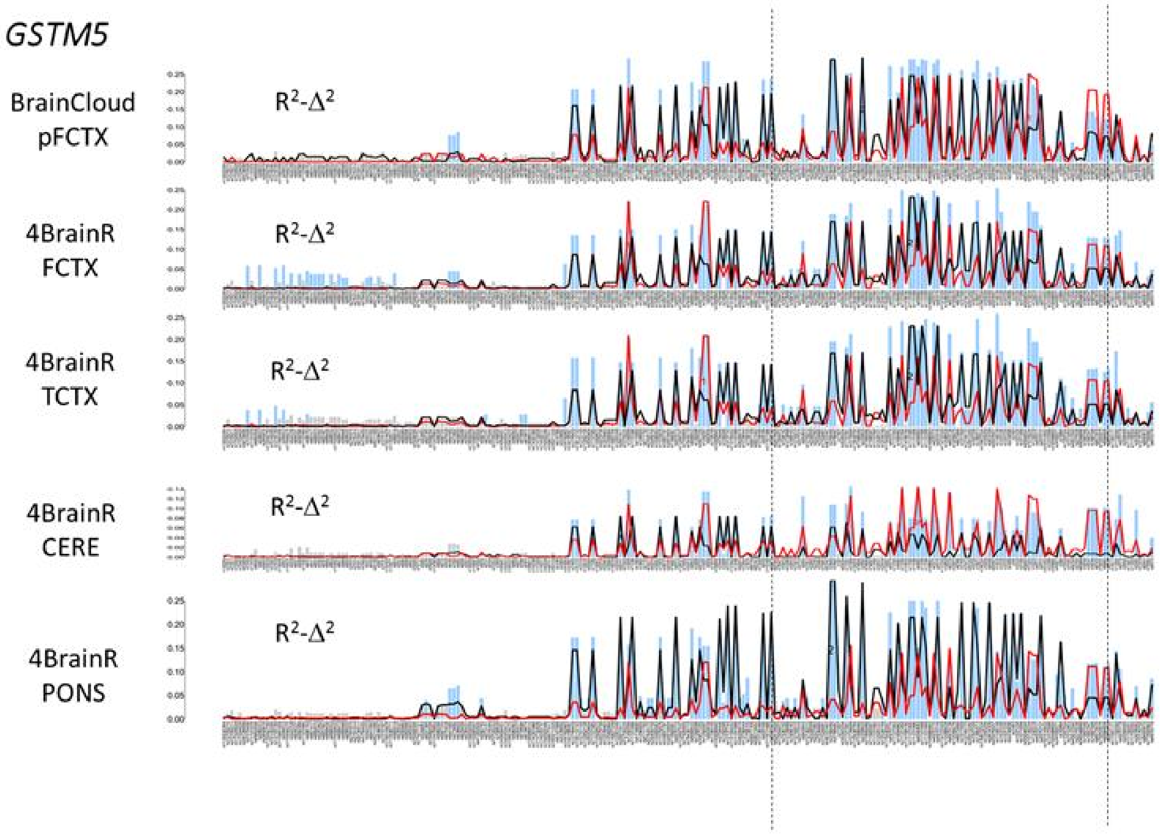

Figure S10.4 Alignment of R^2-Δ^2 plots reveals conservation of “red” and “black” family SNPs in pFCTX, FCTX, TCTX, CERE and pons.

4. Comparison of *GSTM3* versus *GSTM5* iSNP families in pFCTX, FCTX and TCTX

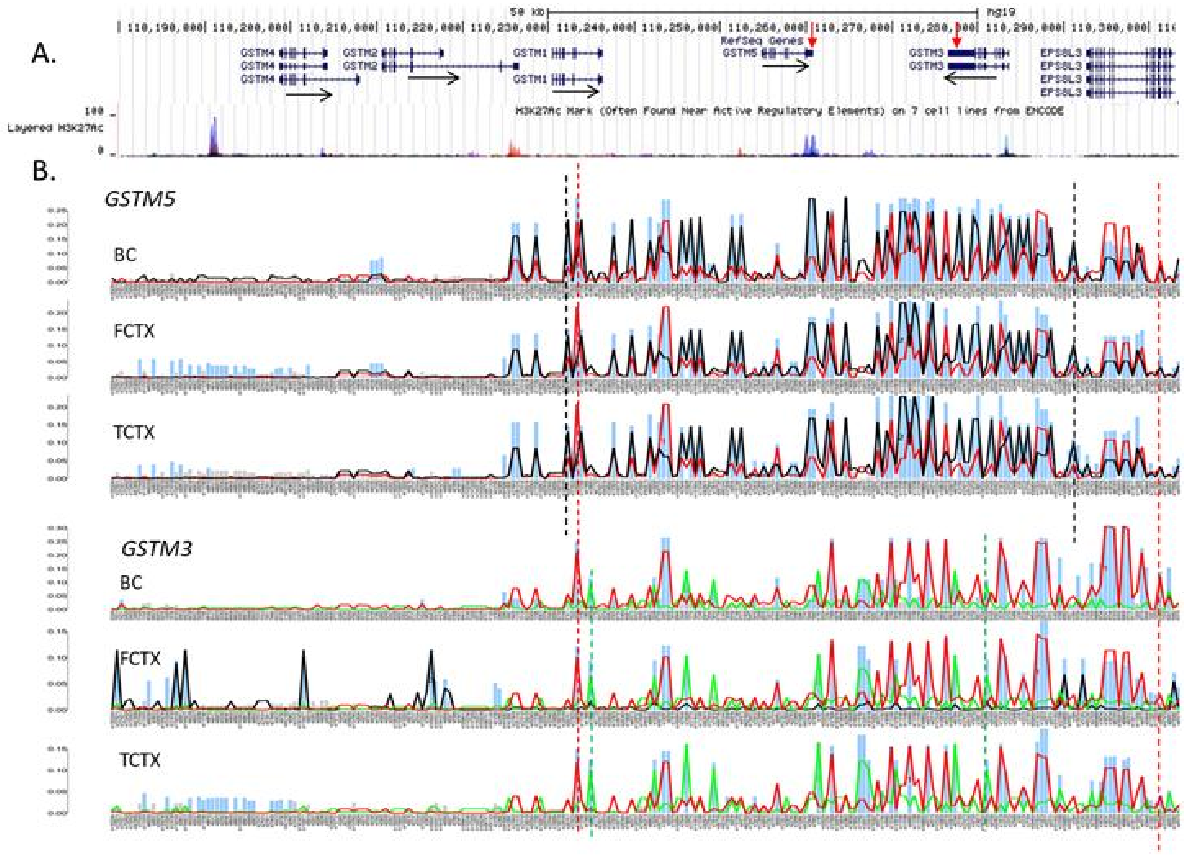

Figure S10.5 Alignment of R^2-Δ^2 plots reveal conservation of "red" family SNPs in *GSTM5* and *GSTM3*. By contrast "black" family SNPs and "green" family SNPs are conserved separately for *GSTM5* and *GSTM3* mRNA expression, respectively. The red arrows indicate the locations of the array hybridization probes used to quantify levels of *GSTM3* and *GSTM5* mRNA in the BC and 4BrainR studies.

Table S10.1 *GSTM3* and *GSTM5* iSNPs

Gene	Data set	Tissue	Family 1 iSNP	Family 2 iSNP	Family 3 iSNP
GSTM3	BC	pFCTX	rs1887547	rs1292096	-
"	4BrainR	FCTX	rs10735234	rs11807	rs140604451
"	"	TCTX	rs4540683	"	-
"	"	CERE	rs4970777	-	-
"	"	PONS	rs10735234	rs1292096	rs149677411*
GSTM5	BC	pFCTX	rs10735234	rs2209825	-
"	4BrainR	FCTX	rs1292099	rs11101995	-
"	"	TCTX	rs1292097	"	-
"	"	CERE	rs4540683	rs35817611	-
"	"	PONS	rs10776699	rs3768490	-

References

[1] C. Colantuoni, B.K. Lipska, T. Ye, T.M. Hyde, R. Tao, J.T. Leek, E.A. Colantuoni, A.G. Elkahloun, M.M. Herman, D.R. Weinberger, J.E. Kleinman, Temporal dynamics and genetic control of transcription in the human prefrontal cortex, Nature. (2011). doi:10.1038/nature10524.

[2] J.R. Gibbs, M.P. van der Brug, D.G. Hernandez, B.J. Traynor, M.A. Nalls, S.L. Lai, S. P: statistical significance of slope of regression line., A. Dillman, I.P. Rafferty, J. Troncoso, R. Johnson, H.R. Zielke, L. Ferrucci, D.L. Longo, M.R. Cookson, A.B. Singleton, Abundant quantitative trait loci exist for DNA methylation and gene expression in Human Brain, PLoS Genet. (2010). doi:10.1371/journal.pgen.1000952.
